# Supplementary material for: General practitioners’ decision-making process to prescribe pain medicines for low back pain: a qualitative study
Source: BMJ Open. 2023 Oct 29;13(10):e074380. doi: 10.1136/bmjopen-2023-074380 (PMC10619041; doi:10.1136/bmjopen-2023-074380)
Supplement: Supplementary data [file bmjopen-2023-074380supp001.pdf]

General practitioners' decision-making process to prescribe pain medicines for low back pain: a qualitative study

Contents

Supplementary file 1. Vignettes 2
Supplementary file 2. Interview guide. 5

Supplementary file 1. Vignettes

Prompt: I will now present to you three clinical vignettes describing different low back pain presentations. After each vignette I am going to ask you a series of questions. Please draw on your prior experience to answer the questions about how you would manage these back pain patients. There are no right or wrong answers. We are interested in honest opinions, not idealised ones (for example, what you should do). Remember that your data will be anonymised.

VIGNETTE 1

- Patient info: Female patient, 57 years old, BMI 33 (height: 1.60m; weight: 85kg)
- Complaint: atraumatic onset low back pain that started 2 weeks ago. Average pain 5/10; worse in the evening/at night with pain 7/10.
- History:
    - Low back pain for 20+ years (ongoing mild pain 2/10) with occasional moderate flares (pain up to 5/10) that typically resolve in a few days. Previous MRIs (last one 3 years ago) showed the following: At L5/S1 there is vertebral endplate bone marrow oedema. The distinction between the nucleus and annulus is lost with a with loss of disc height.
    - She has sought care for back pain a few times before, but typically self-manages the pain with simple painkillers (Paracetamol and occasionally ibuprofen as needed). She is seeking care for this episode because it’s taking longer to resolve, and the pain is more intense in the evening/at night
    - Patient is having difficulty sleeping – she wakes up because of pain when turning in bed.
    - Patient is sedentary and is the primary carer of her 88-year-old mother who is bed-bound due to a stroke 12 months ago.
    - Comorbidities: Obesity, GORD, high cholesterol (daily atorvastatin), medial compartment R knee osteoarthritis (also treats with simple analgesics as needed).
- Physical examination:
    - No evidence of radicular symptoms: straight leg raise, sensation, reflexes, and lower limb strength are normal. No signs of infection or midline tenderness in the lumbar spine. Tenderness over the paraspinal muscles is present.
    - Movements in every direction are slightly more painful (6/10)

VIGNETTE 2

- Patient info: 45-year-old male, BMI 26 (height: 1.83m; weight: 87kg)

- Complaint: Acute onset low back pain with shooting stabbing pain radiating down the back of the right leg to the foot and associated with pins and needles in the same area. This commenced 8 weeks ago after trying to lift a heavy box while visiting family in rural NSW. Main complaint is leg pain (5/10), but he also has pain in the lower back (3/10). Sitting for >1h aggravates leg pain (7/10), sit-to-standing and vice-versa are quite painful (7/10).

- History:
    - No history of low back pain or any other musculoskeletal condition prior to this episode. Patient is physically active, swims and runs twice a week. Sitting for more than 1 hour aggravates pain and is severely limiting his ability to work. He has not taken any time off work.
    - Patient went to the local general practitioner the day after pain onset who prescribed codeine 30mg QID for 1 week. No improvement was noted. No significant side effects were noted either besides constipation.
    - Patient has not reported any significant sleep disturbance

- Physical examination:
    - Pain follows a dermatomal distribution (S1), with altered sensation to light touch. Muscle strength and reflexes are preserved.
    - No signs of infection or midline tenderness in the lumbar spine.
    - Straight leg raise positive at ~30deg; crossed straight leg raise test is negative
    - Flexion and extension movements are limited and increase leg pain (7/10). Rotations and inclinations do not change symptoms.
    - Cauda equina syndrome and Other red flags were excluded

VIGNETTE 3

- Patient info: Female patient, 52 years old, BMI 30 (height: 1.63m; weight: 79kg)

- Complaint: Insidious onset low back pain radiating to the L thigh that started 6 months ago. Patient cannot recall an event that triggered the pain. Pain is bilateral in the lower back and radiates to the L posterior thigh with the same average intensity (5/10). Standing up/walking for too long (>1h) increases pain (6-7/10). Sitting/lying down reduces pain. She does not have trouble sleeping.

- History:
  - No history of low back pain or any other musculoskeletal condition prior to this episode. Patient is married with 3 children (youngest is 20 years-old),
  - Patient has recently been diagnosed with type II diabetes (she takes metformin daily and has been going to the gym 3 times/week for about 45 minutes where she does low-moderate intensity resistance training since the diagnosis – 2 years ago. Her sugar levels are currently controlled – around 5mmol/L before meals. Kidney function is normal). She was diagnosed with mild depression two years ago and has been supported by a psychologist with training in cognitive behavioural therapy. No other pharmacological treatments are used for depression.
  - She uses over-the-counter analgesics (Paracetamol and Ibuprofen) regularly on an as-needed basis to manage the pain. She has seen a physiotherapist (core strengthening exercises) and an acupuncturist but has not responded to either treatment.

- Physical examination:
  - No abnormalities on neurological exam (sensation, strength, and reflexes are all normal).
  - No signs of infection or midline tenderness in the lumbar spine.
  - Straight leg raise and crossed Straight leg raise negative.
  - Lumbar spine movements are limited mostly by lack of flexibility. End-range lumbar extension increases pain momentarily (7/10), which reduces as soon as patient returns to neutral position.

Supplementary file 2. Interview guide.

Questions - Choosing between different types of pharmacological treatments

Prompt: Answer the following questions based on the patient vignette you just read. Using the information from the vignette, I would like you to think aloud your clinical reasoning.

- Would you typically prescribe a pain medicine for the patient described in the vignette?

- Could you elaborate on which pain medicines you would prescribe and why you would prescribe them *[remind the participant of the medicines they mentioned]*?

Prompt participant to talk about their rationale to prescribe/recommend certain medicines; what kind of thought process they follow, sources of information that they use to make their decision:

- *Clinical presentation (e.g. affected sleep in vignette 1; neuropathic pain features in vignette 2; depression in vignette 3, etc.)*
- *Severity of symptoms*
- *Known efficacy from trials and systematic reviews*
- *Guideline recommendation*
- *Safety/side effects*
- *Previous use by patient*
- *Previous success with other patients*
- *Demographic factors*
- *Mechanisms of action of the drug*
- *Intuition*

Questions – influence of factors not mentioned by the participant

- How do aspects like (eg age, gender, comorbidities, description of pain, etc.) factor in your decision to prescribe 'ABC' *[the pain medicines mentioned by the participant]*?

Questions - Alternatives

- What about pain medicines 'XYZ'? Would you prescribe any of these for the vignette? *[mention pain medicines that the participant did not mention eg strong opioids [oxycodone endone], weak opioids [codeine], gabapentin, pregabalin, amitriptyline, oral steroids, etc.)]*

- Are there any circumstances where you would prescribe XYZ instead or/in addition to ABC for the patient in this vignette?

Questions – external factors influencing decision-making

- Would your decision-making change if you were pressed for time? *[eg do they report using any heuristic/rules of thumb when they are time-limited?]*
- Would your decision-making change depending on patients' opinions on the treatments you typically prescribe?
