# Supplementary material for: General practitioners’ decision-making process to prescribe pain medicines for low back pain: a qualitative study
Source: BMJ Open. 2023 Oct 29;13(10):e074380. doi: 10.1136/bmjopen-2023-074380 (PMC10619041; doi:10.1136/bmjopen-2023-074380)
Supplement: Author's manuscript [file bmjopen-2023-074380.draft_revisions.pdf]

BMJ Open

BMJ Open is committed to open peer review. As part of this commitment we make the peer review history of every article we publish publicly available.

When an article is published we post the peer reviewers' comments and the authors' responses online. We also post the versions of the paper that were used during peer review. These are the versions that the peer review comments apply to.

The versions of the paper that follow are the versions that were submitted during the peer review process. They are not the versions of record or the final published versions. They should not be cited or distributed as the published version of this manuscript.

BMJ Open is an open access journal and the full, final, typeset and author-corrected version of record of the manuscript is available on our site with no access controls, subscription charges or pay-per-view fees (http://bmjopen.bmj.com).

If you have any questions on BMJ Open's open peer review process please email info.bmjopen@bmj.com

BMJ Open

General practitioners' decision-making process to prescribe pain medicines for low back pain: a qualitative study

Journal:	*BMJ Open*
Manuscript ID	bmjopen-2023-074380
Article Type:	Original research
Date Submitted by the Author:	04-Apr-2023

Complete List of Authors:	Ferreira, Giovanni; University of Sydney School of Public Health, Institute for Musculoskeletal Health<br>Zadro, Joshua; University of Sydney, Institute for Musculoskeletal Health, School of Public Health<br>Jones, Caitlin; The University of Sydney Institute for Musculoskeletal Health, School of Public Health<br>Ayre, Julie; The University of Sydney, Sydney Health Literacy Lab<br>Lin, Christine; University of Sydney School of Public Health, Institute for Musculoskeletal Health<br>Richards, Bethan; The University of Sydney Institute for Musculoskeletal Health<br>Needs, Christopher; Institute of Rheumatology & Orthopaedics, Rheumatology Department<br>Abdel Shaheed, Christina; University of Sydney School of Public Health, Institute for Musculoskeletal Health<br>McLachlan, Andrew; University of Sydney, Faculty of Pharmacy<br>Day, Richard; St Vincent's Hospital, Department of Clinical Pharmacology & Toxicology; University of New South Wales, St Vincent's Clinical School, Faculty of Medicine<br>Maher, Christopher; University of Sydney, Institute for Musculoskeletal Health, Sydney School of Public Health, Faculty of Medicine and Health

Keywords:	Decision Making, Back pain < ORTHOPAEDIC & TRAUMA SURGERY, QUALITATIVE RESEARCH

SCHOLARONE™
Manuscripts

I, the Submitting Author has the right to grant and does grant on behalf of all authors of the Work (as defined in the below author licence), an exclusive licence and/or a non-exclusive licence for contributions from authors who are: i) UK Crown employees; ii) where BMJ has agreed a CC-BY licence shall apply, and/or iii) in accordance with the terms applicable for US Federal Government officers or employees acting as part of their official duties; on a worldwide, perpetual, irrevocable, royalty-free basis to BMJ Publishing Group Ltd ("BMJ") its licensees and where the relevant Journal is co-owned by BMJ to the co-owners of the Journal, to publish the Work in this journal and any other BMJ products and to exploit all rights, as set out in our licence.

The Submitting Author accepts and understands that any supply made under these terms is made by BMJ to the Submitting Author unless you are acting as an employee on behalf of your employer or a postgraduate student of an affiliated institution which is paying any applicable article publishing charge ("APC") for Open Access articles. Where the Submitting Author wishes to make the Work available on an Open Access basis (and intends to pay the relevant APC), the terms of reuse of such Open Access shall be governed by a Creative Commons licence – details of these licences and which Creative Commons licence will apply to this Work are set out in our licence referred to above.

Other than as permitted in any relevant BMJ Author's Self Archiving Policies, I confirm this Work has not been accepted for publication elsewhere, is not being considered for publication elsewhere and does not duplicate material already published. I confirm all authors consent to publication of this Work and authorise the granting of this licence.

For peer review only

General practitioners' decision-making process to prescribe pain medicines for low back pain: a qualitative study

Giovanni E Ferreira[1,2], Joshua R Zadro[1,2], Caitlin Jones[1,2], Julie Ayre[1,2], Chung-Wei Christine Lin[1,2], Bethan Richards[2,4], Christopher Needs[4], Christina Abdel Shaheed[1,2], Andrew McLachlan[5], Richard Day[6], Chris G Maher[1,2]

1. Sydney Musculoskeletal Health, Faculty of Medicine and Health, The University of Sydney
2. Institute for Musculoskeletal Health, The University of Sydney and Sydney Local Health District
3. Sydney Health Literacy Lab, School of Public Health, Faculty of Medicine and Health, The University of Sydney, Sydney, Australia
4. Department of Rheumatology, Royal Prince Alfred Hospital, Sydney, Australia
5. Sydney Pharmacy School, Faculty of Medicine and Health, The University of Sydney, Sydney, Australia
6. St Vincent's Clinical School, Department of Clinical Pharmacology & Toxicology, University of New South Wales, Sydney, Australia

For peer review only

Abstract word count: 245
Manuscript word count: 3955

Address all correspondence to:

Giovanni E. Ferreira
School of Public Health, Faculty of Medicine and Health, The University of Sydney, Sydney, New South Wales, Australia
PO Box M179, Missenden Road, Camperdown | NSW | 2050, Australia
E-mail: giovanni.ferreira@sydney.edu.au

For peer review only - http://bmjopen.bmj.com/site/about/guidelines.xhtml

ABSTRACT

Background: Pain medicines are widely prescribed by general practitioners (GPs) when managing people with low back pain (LBP), but little is known about what drives decisions to prescribe these medicines.

Objectives: The aim of this study was to investigate what influences GPs' decision to prescribe pain medicines for LBP.

Design: Qualitative study with in-depth interviews.

Setting: Australian primary care.

Participants: We interviewed 25 GPs practising in Australia experienced in managing LBP (mean [SD] age 53.4 [9.1] years, mean [SD] years of experience: 24.6 [9.3], 36% female). GPs were provided three vignettes describing common LBP presentations (acute exacerbation of chronic LBP, subacute sciatica, and chronic LBP) and were asked to think aloud how they would manage the cases described in the vignettes.

Data analysis: We summarised GP's choices of pain medicines for each vignette using content analysis and used framework analysis to investigate factors that affected GP's decision-making.

Results: GPs more commonly prescribed opioid analgesics. Anticonvulsants and antidepressants were also commonly prescribed depending on the presentation describe in the vignette. GP participants made decisions about what pain medicines to prescribe for LBP largely based on previous experiences, including their own personal experiences of LBP, rather than guidelines. The choice of pain medicine was influenced by a range of clinical factors, more commonly the patient's pathoanatomical diagnosis. While many adhered to principles of judicious use of pain medicines, polypharmacy scenarios were also common. Concerns about drug seeking behaviour, adverse effects, stigma around opioid analgesics, and pressure from regulators also shaped their decision-making process.

Conclusions: We identified several aspects of decision-making that help explain the current profile of pain medicines prescribed for LBP in by GPs. Themes identified by our study could inform future implementation strategies to improve the quality use of medicines for LBP.

Keywords: decision-making, opioids, low back pain, prescription, sciatica

For peer review only - http://bmjopen.bmj.com/site/about/guidelines.xhtml

Strengths and limitations of the study

- We used clinical vignettes resembling common low back pain cases to provide clinicians with a concrete starting point where they could base their decisions on
- Voices and perspectives of younger general practitioners or general practitioners working in rural and remote areas may have been underrepresented.

For peer review only

For peer review only - http://bmjopen.bmj.com/site/about/guidelines.xhtml

INTRODUCTION

Low back pain (LBP) affects over 4 million Australians every year and pain medicines are widely used in its management.[1] Australian data shows that 2 in every 3 patients who seek primary care take, or are prescribed, at least one pain medicine.[2] Examples include over-the-counter analgesics (e.g. paracetamol, and non-steroidal anti-inflammatories (NSAIDs)) and prescription medicines such as opioid analgesics, gabapentinoids, antidepressants, and muscle relaxants.[3]

There is a mismatch between the pharmacological therapies recommended in clinical practice guidelines for LBP and what is prescribed or recommended to patients in primary care. For example, opioid analgesics are one of the most commonly prescribed medicines for people presenting with LBP across various settings and their use has increased 39% in the last decade.[3] This is despite guidelines discouraging their use for LBP when pain is not severe or as first-line care before trialling other treatments.[3,4] Similarly, gabapentinoids are ineffective and potentially harmful for the management of LBP; however their use is increasing.[4-6] On the other hand, the use of pharmacological therapies that are recommended as first line care such as NSAIDs has reduced 33% from 2004 to 2014.[3] In the United States, antidepressants are prescribed to about a quarter of patients with LBP.[7] In Portugal, 1 in 7 people with chronic LBP report using antidepressants and benzodiazepines for pain management.[8] However, LBP guidelines recommend against both medicines.[4]

In Australia, LBP is the sixth most commonly managed condition overall and the most common musculoskeletal condition managed by general practitioners (GPs).[9] A UK-based study provided some insight into aspects that shape the decision-making process of health professionals, including GPs. That study described how clinicians select treatments based upon knowing the individual patient, the patient's LBP, and common treatment options.[10] However, this previous study did not explore the decision-making processes that influence a GP's decision to prescribe pain medicines for LBP in-depth, particularly in relation to the processes that underpin the choice for different pain medicines. Therefore, the aim of this qualitative study was to investigate what influences GPs' decision-making process for prescribing pain medicines for people with LBP.

METHODS

Design

This qualitative study was approved by XXXXX Human Research Ethics Committee (approval number: 2022/170) and is reported per the COREQ statement.[11]

Participant selection and recruitment

We recruited 25 GPs registered to practise in Australia who reported having provided care for at least five patients with LBP with or without sciatica in the last 12 months. We defined sciatica as a back problem with radiating pain below the knee with or without neurological symptoms (e.g. reduced muscle strength, sensation, or reflexes).[12]

For peer review only - http://bmjopen.bmj.com/site/about/guidelines.xhtml

GPs were recruited via a market research company (TKW Research Group, Australia) with a large database of healthcare professionals across Australia and New Zealand. Health professionals opt into this database and therefore have already indicated a willingness to participate in research. None of the participants had a prior relationship with the study investigators.

Interviews and data collection

We conducted in-depth, one-on-one semi-structured interviews. Interviews were conducted by videoconference between May and June 2022, audio recorded, and transcribed verbatim. A researcher with a background in occupational therapy and experience in qualitative interviews and pain management research conducted the interviews.

An interview guide and three clinical vignettes to guide the interviews were developed by the lead author, a physiotherapist with experience in LBP research and pain management, and two experienced rheumatologists who provide care for patients with LBP (supplementary file 1). The case vignettes were designed to represent three common LBP presentations: a patient with an acute exacerbation of chronic LBP, a patient with subacute sciatica (pain duration between 6-12 weeks), and a patient with chronic non-specific LBP (pain for longer than 12 weeks) with no sciatica. Collectively, the vignettes were modelled to describe a range of factors that could play a role in a doctors' decision-making process to manage LBP (Table 1). GPs were asked to think aloud how they would manage the cases described in the vignettes.[13] For each clinical decision made by the participant, the researcher facilitating the interview asked participants to elaborate on a point, or to obtain clarification if needed (Supplementary file 2).

Analysis

Interview data were analysed using two approaches. We used content analysis to create a summary of GP's choices of pain medicines prescribed for each vignette. Content analysis combines both qualitative and quantitative methods, allowing both the content and frequency of categories to be reported.[14,15] Two researchers initially reviewed and familiarised themselves with the transcripts, and coded all pain medicines mentioned by GPs using the following framework: we classified pain medicines as those that would be prescribed as first, second, third, or fourth-line care, those that GPs would conditionally prescribe (e.g. depending on a patient characteristic; a GP would prescribe one type of opioid analgesic but would have concerns about another type), and those that GPs were against prescribing. The two researchers then met to compare, discuss, and reach consensus on the codes.

We used framework analysis to investigate factors that influence GP's decision-making process.[16] Two researchers coded 10 interview transcripts inductively, developed a library of codes in an iterative process, decided on a coding framework (comprising the themes) and applied it to all interviews. Codes, sub-themes, and themes were reviewed by us, and changes were made as stronger patterns linking codes to themes emerged from the data. Both researchers have a background in physiotherapy and have published on the quality use of pain medicines.[17-19]

For peer review only - http://bmjopen.bmj.com/site/about/guidelines.xhtml

Patient and public involvement

It was not appropriate or possible to involve patients or the public in the design, or conduct, or reporting, or dissemination plans of our research.

RESULTS

We interviewed 25 GPs from April to June, 2022. Most were males (64%) practising in metropolitan areas across six states of Australia. Their mean (SD) age was 53.4 (9.1), and they had a mean of 24.5 (9.3) years of practice as GPs. All but three worked full-time in private practices (Table 2)

Content analysis

Paracetamol and NSAIDs were commonly for the cases outlined in all three vignettes. However, opioid analgesics were the pain medicines most commonly prescribed or conditionally prescribed for the acute exacerbation of chronic LBP and sciatica vignettes, and antidepressants for the chronic LBP vignette. Codeine was the most commonly prescribed opioid analgesic for the acute exacerbation vignette, whereas tramadol was the most common choice for the sciatica vignette. Concerns over the safety of opioids, particularly codeine, were commonly reported by GPs across all vignettes. Those concerns were often specific to a type of opioid, as many who expressed concerns over one type of opioid would still prescribe a different opioid analgesic or conditionally prescribe the same opioid. For example, this was observed in the interviews of 11 (44%) GPs in the acute exacerbation vignette (Table 3).

Anticonvulsants were the medicine class with the highest number of GPs indicating they would not prescribe for the acute exacerbation vignette: nine (36%) were against prescribing them because the patient did not have signs of neuropathic pain that would justify their use. In contrast, 16 (64%) of GPs recommended an anticonvulsant for the sciatica vignette, mostly pregabalin. GPs prescribed antidepressants for all three vignettes, more commonly for the chronic LBP vignette (n=15, 63%). Of those, most chose duloxetine (n=10, 42%) and/or amitriptyline (n=7, 29%). GPs prescribed antidepressants for different reasons for each vignette: co-morbid depression influenced decision-making for the chronic LBP vignette, whereas neuropathic pain was the key reason leading to antidepressants being prescribed for the sciatica vignette.

Factors influencing GP's decision-making to prescribe pain medicines for LBP

We identified 5 key themes that influenced GP's decision-making. Themes, subthemes, and supporting quotes are presented in table 4.

Theme 1: Prioritising personal information sources

Subtheme 1 - Previous experiences: Past clinical and personal experiences were recognised by most GPs as the most important source of information to help them make decisions. Some acknowledged that accumulated clinical experience contributed to the formulation of shortcuts that guided decision-making. Meaningful past experiences were often centred

For peer review only - http://bmjopen.bmj.com/site/about/guidelines.xhtml

around success using medicines with patients, but also personal success when they experienced pain themselves.

GPs considered the patient's previous experience with treatments an important source of information to assist with their decision-making. Giving patients options and discussing those options with them was often mentioned. However, in those circumstances, patient preferences and values appeared to be the key drivers of decision-making. Discussion about the evidence-base for each option was less prominent.

Subtheme 2 - Guidelines are unhelpful: Many GPs reported that guidelines were not helpful to their practice. Guideline recommendations were noted to be particularly unhelpful when patients required more than minimal intervention such as advice and simple analgesics. Recommended interventions such as advice and education about the benign nature of most LBP and advice to stay active were seen as problematic as it could be hard to convince patients in pain of their usefulness. One GP noted that the problem with guidelines was not necessarily their recommendations, but the time required to implement them in a context of GPs pressured for time.

Subtheme 3 - Clinical training: While the applicability of guidelines was questioned, continuing education activities run by specialists and opinion leaders were highly valued. By comparison, training programs were rarely mentioned; only one GP with five years of experience mentioned the role of medical school and GP registrar training in addressing the current overuse use of opioids for LBP management.

Theme 2: Strong reliance on a biomedical model to guide prescription

Subtheme 1 - Pathoanatomical diagnosis: There was a strong sense amongst participating GPs that a pathoanatomical diagnosis was key to inform the choice of pain medicine to be prescribed. This was more pronounced for the chronic LBP vignette where, according to GPs, there was a more uncertain clinical presentation due to the description and duration of symptoms as well as comorbidities. Many GPs reported they would have referred the patient for imaging, and many considered the number and nature of imaging findings a key indicator of severity of the problem, which they linked to the prescription of stronger analgesics.

Subtheme 2 - Sleep patterns: Sleep influenced decision-making across the three vignettes, even though the patient in the chronic LBP vignette had no night pain or sleep disturbance. Improving sleep was an important treatment goal in the acute exacerbation vignette. For some, improving sleep was seen as the primary goal of treatment, and often led GPs to prescribe an opioid analgesics or antidepressants, or both.

Subtheme 3 - Pain severity: There were opposing views on the role of pain severity as a factor that influenced decision-making. While some were happy to consider stronger analgesics when pain was more severe, for others pain severity would not prompt immediate escalation to stronger analgesics. GPs were more inclined to consider escalating to stronger analgesics when discussing the acute exacerbation vignette and less inclined to do so in the chronic LBP vignette (where the average pain intensity was described as the same: 5 out of 10). For some GPs who would not consider escalating to stronger analgesics, their initial

For peer review only - http://bmjopen.bmj.com/site/about/guidelines.xhtml

choice of treatment was typically a weak opioid analgesic such as codeine. Those preferring codeine mentioned being more used to the prescribing it than other opioid analgesics.

Subtheme 4 – Comorbidities: Comorbidities described in the vignettes were noted by most and influenced decision-making for some. Losing weight was often mentioned as a treatment goal in all three vignettes and led some GPs to preferring non-pharmacological approaches first or avoiding pharmacological treatments known to cause weight gain (e.g. some antidepressants), despite obesity only being mentioned in the acute exacerbation vignette. The history of mild depression described in the chronic LBP vignette led many GPs to consider an antidepressant. The absence of depression in the subacute sciatica vignette was an important factor for GPs when deciding between different pharmacological options for neuropathic pain symptoms. Many acknowledged the role of antidepressants such as duloxetine and amitriptyline as potentially effective pain medicines for neuropathic symptoms as described in the sciatica vignette, but most preferred anticonvulsants as the patient was young, otherwise healthy, physically active, and did not have chronic pain. In contrast, most GPs preferred to prescribe duloxetine for patients with comorbid depression and chronic pain such as the patient described in chronic LBP vignette.

Theme 3: Social factors as root causes

Subtheme 1 - Pain medicines do not address the root cause of the problem: Many GPs reported that prescribing medicines for pain for the acute exacerbation vignette would not be likely to address the root cause of the problem. Some suggested non-pharmacological treatments such as lifestyle interventions, but most GPs expressed concern about the level of social and family support that the patient had and its potential negative impact on her LBP.

Subtheme 2 - Psychosocial-based prescribing: Some GPs considered the patient's social circumstances when deciding whether to prescribe a pain medicine and what to prescribe. In the acute exacerbation vignette, many thought that the patient's social circumstances were important contributors to her pain but from different perspectives. While some emphasised the physical strain related to her carer role as a contributor to the pain, others recognised the psychological consequences of the patient's social circumstances. Those who acknowledged physical factors preferred recommending non-pharmacological options such as ergonomic devices, whereas those who recognised the psychological toll of the patient's carer role more commonly prescribed opioid analgesics or antidepressants. Other GPs were wary about whether side effects from opioid analgesics would hinder the patient's ability to care for her mother due to potential side effects, such as sedation.

Theme 4: Judicious use of pain medicines

Subtheme 1 - Keep it simple or make it complex? There were opposing views about judicious use of pain medicines. Many GPs followed the principles of the WHO analgesic ladder and avoided polypharmacy, particularly for the acute exacerbation vignette, and, to a lesser extent, the sciatica vignette. For GPs following that approach, there was a perception that the clinical course of both vignettes would be favourable and keeping prescription of pain medicines to a minimum, combined with non-pharmacological measures, would be effective. Chronic pain and the history of depression were seen as barriers to improvement in the

For peer review only - http://bmjopen.bmj.com/site/about/guidelines.xhtml

chronic LBP vignette – and therefore to a perception that the patient with chronic LBP would have been more complex to manage.

Other GPs reported that they preferred prescribing multiple pain medicines in combination, at least in the short-term, and most commonly in the sciatica and chronic LBP vignettes. In the sciatica vignette, this was driven by two factors: the notion that there was a well-established mechanism of injury (as opposed to a degenerative process with no known trigger as in the acute exacerbation vignette), and the perception that the clinical presentation was more serious as the patient had radicular symptoms. Both factors led GPs to consider stronger pain medicines such as opioid analgesics and anticonvulsants. In the chronic LBP vignette, polypharmacy often included the prescription of simple analgesics with antidepressants, most commonly duloxetine, which was driven mainly by the history of mild depression.

Theme 5: Concerns about prescription

Subtheme 1 - Drug seeking behaviour and risk of long-term misuse: A key concern for many GPs was drug seeking behaviour. GPs used multiple strategies to identify drug seeking behaviours, such as a review of medical history and intuition. Some were reluctant to prescribe opioid analgesics to patients who were not their regular patients. Some GPs mentioned being able to identify certain stereotypes more likely to be drug seekers. Although opioids were commonly considered by GPs across the three vignettes, most had concerns about their potential for misuse and addiction, particularly in relation to the chronic LBP vignette. Chronic pain and a history of depression were seen as risk factors to long-term misuse and addiction to opioid analgesics.

Subtheme 2 - Side effects of prescription pain medicines: Similarly, most concerns about side effects were related to opioid analgesics. Many GPs expressed concerns about the safety profile of specific opioid analgesics, most commonly codeine, and made decisions about which opioid analgesic to use based on their knowledge about the safety profile of opioid analgesics. In contrast, very few GPs expressed concerns about other pain medicines with potential for misuse and abuse, such as pregabalin.

Subtheme 3 - Stigma around prescription: Some GPs were aware of the stigma around prescribing pain medicines such as antidepressants. Although frequently mentioned as a treatment option for the sciatica and chronic LBP vignettes, some had concerns that prescribing antidepressants had potential for misinterpretation by patients – for example, that they were implying that the patient's pain was psychological. Lack of time during a regular consultation was seen as important barrier for prescribing antidepressants. There was a perception that longer consultations would be required to explain why the medication was being prescribed to mitigate any concerns from patients.

Subtheme 4 - Pressure from regulators: Some GPs were concerned about being pressured by regulators to not prescribe pain medicines such as opioid analgesics. Their concerns included the potential consequences of undertreating pain. No GP mentioned that pressure from regulators had changed their prescribing behaviour.

DISCUSSION

For peer review only - http://bmjopen.bmj.com/site/about/guidelines.xhtml

The GPs who participated in this study made decisions about what pain medicines to prescribe for LBP largely based on previous experiences, including their own clinical and personal experience of LBP management, not guidelines. The choice of pain medicine was influenced by a range of clinical factors, more commonly the patient's pathoanatomical diagnosis. While many adhered to principles of pain medicines prescribing, polypharmacy scenarios were also common. Concerns about drug seeking behaviour, side effects, stigma around prescription, but not pressure from regulators, also shaped GPs' decision-making process.

Antidepressants were considered by GPs in our study in two instances: where they thought depression was potentially linked to the patient's symptoms (acute exacerbation and chronic LBP vignettes) or as a pain medicine to treat neuropathic pain (sciatica vignette). Although there is some evidence that antidepressants may be effective for those with chronic pain and comorbid depression and neuropathic pain,[19] the role of antidepressants for chronic LBP is limited and the evidence for sciatica is inconclusive.[17] Some GPs voiced concerns about stigma around prescribing antidepressants for pain that was not observed for any other pain medicine in our study. Stigma around the use of antidepressants is a well-recognised phenomenon in the mental health field, where studies have shown that people using antidepressants for depression have a perception that their condition is more severe, that they are weak or unable to cope with their problems, and that they are under the effects of a drug.[20] We have shown that GPs in our study had the perception that patients may not receive the idea of being prescribed an antidepressant for their pain well. Future studies could investigate the extent of stigma associated with use of antidepressants for pain from the patients' perspective.

While our GPs were aware of the harms and potential for misuse and abuse of opioid analgesics, fewer mentioned concerns with other prescription pain medicines such as anticonvulsants, which also have potential for misuse, addiction, and death.[21] The apparent lack of awareness about the harms of these medicines appears to also be common amongst the public.[22] In contrast, public health entities and government regulatory agencies have recognised the concerning potential harms of these medicines and have taken action to reduce them. Such concerns have led regulatory agencies in the UK and Australia to up schedule and to add Boxed Warnings to the Product Information and Consumer Medicine Information for pregabalin, respectively.[21,23]

Time constraints in general practice have long been linked to poor adherence to guidelines, inappropriate prescription, and provision of worse care for chronic disease.[24-26] Furthermore, it has been shown that meeting guideline recommendations in primary care requires an unrealistic amount of time that GPs do not have.[27] In line with these findings, there was a perception amongst some GPs in our study that the time required to implement guideline recommendations into practice, or to discuss prescription of certain pain medicines (e.g. antidepressants), was an important barrier to their prescription. Several studies have pointed out that GPs perceive it as nearly impossible to meet all guideline recommendations as there are too many recommendations and most are complex. For example, implementing and

For peer review only - http://bmjopen.bmj.com/site/about/guidelines.xhtml

documenting all recommendations from guidelines for common problems seen in general practice would take up to 27 hours per day.[28] LBP guidelines involve a complex series of steps from triage to management strategies, and may also take considerable time to be implemented [29] – although this has not been investigated yet.

Our findings provide insights for future research aiming to improve the quality use of medicines for LBP in general practice. For example, GPs in our study rarely made decisions based on guidelines or evidence. Rather, they strongly relied on previous personal experience with pain medications, and interactions with patients, colleagues, and opinion leaders. Our observations are in line with previous work that has shown that GPs make decisions mainly based on interaction with others rather than endorsed guidelines – a process that has been referred to in the literature as '*mindlines'* (as opposed to 'guidelines').[30]

There are opportunities to develop educational interventions to address some of the factors that influenced decision-making that do not rely on guideline dissemination and consider the resources and sources of information most valued by GPs. One such factor is the inaccurate belief that certain pathoanatomic diagnoses on their own should guide the decision to prescribe certain pain medicines. Pathoanatomic findings are often incidental and have been shown to be mostly unrelated to the development, or severity of symptoms.[31] Interventions could also be developed to increase GPs' awareness of their own biases and heuristics as identified by our study.

The Australian Commission on Safety and Quality in Health Care has recently launched a Clinical Care Standard for LBP which aims to improve the early assessment, management, review and appropriate referral of patients with LBP. One of the core messages of the clinical care standard is around the quality use of medicines for LBP. Specifically, they recommend against anticonvulsants, benzodiazepines, and antidepressants, and that clinicians only consider opioid analgesics for carefully selected patients at the lowest dose for the shortest duration possible.[32] Strategies to implement the Clinical Care Standard may need to account for the perception amongst GPs that following guidelines may lead to patients being undertreated. Our study showed that GPs value opinion leaders as a source of information, which suggests that implementation strategies to improve the quality use of medicines for LBP could involve opinion leaders. Opinion leaders used alone or in combination with other interventions has been shown to improve compliance with evidence-based care.[33]

A strength of our study is the use of vignettes to explore GPs' decision-making process, a widely used methodology for that purpose.[34] Vignettes provided a concrete starting point so that GPs in our study could base their decisions on a detailed patient case, rather than answering abstract questions about attitudes and perceptions. However, the decision-making processes shown in our study were anchored to the information provided in the vignettes and may not be generalisable to all LBP cases. [35] Our sample was composed mostly of very experienced GPs practising in metropolitan areas. Views of more experienced GPs may differ from those in earlier stages of their careers. For example, the youngest GP in our sample mentioned the role of University and GP registrar training in teaching GPs about the lack of effectiveness and harms of opioids for LBP. That view was not shared by any other

For peer review only - http://bmjopen.bmj.com/site/about/guidelines.xhtml

experienced GP, which could indicate recent changes in teaching curricula. GPs practising in rural areas are more likely to prescribe opioid analgesics for LBP.[3] However, our sample lacked GPs practising in those areas, limiting our ability to contrast potential differences in drivers of decision-making between those areas.

CONCLUSIONS

We identified several aspects of decision-making that help explain the current profile of pain medicines prescribed for LBP in by GPs. Themes identified by our study could inform future implementation strategies to improve the quality use of medicines for LBP.

CONTRIBUTORS

All authors designed the study protocol. GF, BR, CN developed the clinical vignettes. CJ conducted interviews. GF and JZ analysed the data. JA provided guidance on data analysis and interpretation. All other authors revised it critically for important intellectual content. All authors approved the final version of the article. All authors had access to all the data in the study and can take responsibility for the integrity of the data and the accuracy of the data analysis. GF is guarantor.

TRANSPARENCY

The lead author (GF) affirms that this manuscript is an honest, accurate, and transparent account of the study being reported; that no important aspects of the study have been omitted; and that any discrepancies from the study as planned (and, if relevant, registered) have been explained.

FUNDING

This study was partially funded by a seed grant from the Australian New Zealand Musculoskeletal Clinical Trials Network (ANZMUSC). GEF, JZ, JA, C-WC L, and CM are supported by National Health and Medical Research Council (NHMRC) fellowships (APP2009808, APP1194105, APP2017278, APP1193939, and APP11094283, respectively). CM is also supported by Centre for Research Excellence grants (APP1134856, APP1171459, APP2006545).

COMPETING INTERESTS DECLARATION

The Sydney Pharmacy School receives funding from GlaxoSmithKline for a postgraduate scholarship supervised by AM. CM has received research grants from various government and not for profit agencies. Flexeze provided heat wraps at no cost for the SHaPED trial for which CM, BR, and CN are investigators. Other authors have no interests to declare.

ETHICAL APPROVAL

The University of Sydney Human Research Ethics Committee approved this research (approval number: 2022/170).

DATA AVAILABILITY

Data are available upon request.

DATA SOURCE

Data collected by the investigators.

LICENSE STATEMENT

The corresponding author (GF) has the right to grant and does grant on behalf of all authors of the Work (as defined in the below author licence), an exclusive licence and/or a non-exclusive licence for contributions from authors who are: i) UK Crown employees; ii) where BMJ has agreed a CC-BY

For peer review only - http://bmjopen.bmj.com/site/about/guidelines.xhtml

licence shall apply, and/or iii) in accordance with the terms applicable for US Federal Government officers or employees acting as part of their official duties; on a worldwide, perpetual, irrevocable, royalty-free basis to BMJ Publishing Group Ltd ("BMJ") its licensees and where the relevant Journal is co-owned by BMJ to the co-owners of the Journal, to publish the Work in BMJ Quality & Safety and any other BMJ products and to exploit all rights, as set out in our licence.

The corresponding author (GF) accepts and understands that any supply made under these terms is made by BMJ to the Submitting Author unless you are acting as an employee on behalf of your employer or a postgraduate student of an affiliated institution which is paying any applicable article publishing charge ("APC") for Open Access articles. Where the Submitting Author wishes to make the Work available on an Open Access basis (and intends to pay the relevant APC), the terms of reuse of such Open Access shall be governed by a Creative Commons licence – details of these licences and which Creative Commons licence will apply to this Work are set out in our licence referred to above.

REFERENCES

1. Australian. *Back problems*. 2020. https://www.aihw.gov.au/reports/chronic-musculoskeletal-conditions/back-problems
2. Williams CM, Maher CG, Hancock MJ, et al. Low back pain and best practice care: A survey of general practice physicians. *Arch Intern Med*. Feb 8 2010;170(3):271-7. doi:10.1001/archinternmed.2009.507
3. Mathieson S, Valenti L, Maher CG, et al. Worsening trends in analgesics recommended for spinal pain in primary care. *Eur Spine J*. May 2018;27(5):1136-1145. doi:10.1007/s00586-017-5178-4
4. NICE. Low back pain and sciatica in over 16s: assessment and management. https://www.nice.org.uk/guidance/ng59
5. Enke O, New HA, New CH, et al. Anticonvulsants in the treatment of low back pain and lumbar radicular pain: a systematic review and meta-analysis. *Cmaj*. Jul 3 2018;190(26):E786-e793. doi:10.1503/cmaj.171333
6. Mathieson S, Maher CG, McLachlan AJ, et al. Trial of Pregabalin for Acute and Chronic Sciatica. *New England Journal of Medicine*. 2017/03/23 2017;376(12):1111-1120. doi:10.1056/NEJMoa1614292
7. Ivanova JI, Birnbaum HG, Schiller M, Kantor E, Johnstone BM, Swindle RW. Real-world practice patterns, health-care utilization, and costs in patients with low back pain: the long road to guideline-concordant care. *Spine J*. Jul 2011;11(7):622-32. doi:10.1016/j.spinee.2011.03.017
8. Gouveia N, Rodrigues A, Ramiro S, et al. The Use of Analgesic and Other Pain-Relief Drugs to Manage Chronic Low Back Pain: Results from a National Survey. https://doi.org/10.1111/papr.12455. *Pain Practice*. 2017/03/01 2017;17(3):353-365. doi:https://doi.org/10.1111/papr.12455
9. Britt H MG, Bayram C, Henderson J, Valenti L, Harrison C, Pan Y, Charles J,, Pollack AJ CT, Gordon J, Wong C. *A decade of Australian general practice activity 2006–07 to 2015–16.* General practice series no 41. Sydney University Press, 2016; 2016.
10. Bishop FL, Dima AL, Ngui J, et al. "Lovely Pie in the Sky Plans": A Qualitative Study of Clinicians' Perspectives on Guidelines for Managing Low Back Pain in Primary Care in England. *Spine (Phila Pa 1976)*. Dec 2015;40(23):1842-50. doi:10.1097/brs.0000000000001215
11. Tong A, Sainsbury P, Craig J. Consolidated criteria for reporting qualitative research (COREQ): a 32-item checklist for interviews and focus groups. *Int J Qual Health Care*. Dec 2007;19(6):349-57. doi:10.1093/intqhc/mzm042
12. Jensen RK, Kongsted A, Kjaer P, Koes B. Diagnosis and treatment of sciatica. *BMJ*. 2019;367:l6273. doi:10.1136/bmj.l6273
13. Lundgren-Laine H, Salantera S. Think-aloud technique and protocol analysis in clinical decision-making research. *Qual Health Res*. Apr 2010;20(4):565-75. doi:10.1177/1049732309354278
14. O'Keeffe M, Michaleff ZA, Harris IA, et al. Public and patient perceptions of diagnostic labels for non-specific low back pain: a content analysis. *European Spine Journal*. 2022/12/01 2022;31(12):3627-3639. doi:10.1007/s00586-022-07365-x

For peer review only - http://bmjopen.bmj.com/site/about/guidelines.xhtml

15. Ferreira GE, Zadro J, O'Keeffe M, et al. A content analysis of online information about the benefits and harms of spine surgery. *Braz J Phys Ther*. Mar-Apr 2022;26(2):100398. doi:10.1016/j.bjpt.2022.100398
16. Gale NK, Heath G, Cameron E, Rashid S, Redwood S. Using the framework method for the analysis of qualitative data in multi-disciplinary health research. *BMC Medical Research Methodology*. 2013/09/18 2013;13(1):117. doi:10.1186/1471-2288-13-117
17. Ferreira GE, McLachlan AJ, Lin CC, et al. Efficacy and safety of antidepressants for the treatment of back pain and osteoarthritis: systematic review and meta-analysis. *BMJ*. Jan 20 2021;372:m4825. doi:10.1136/bmj.m4825
18. Ferreira GE, Machado GC, Abdel Shaheed C, et al. Management of low back pain in Australian emergency departments. *BMJ Qual Saf*. Oct 2019;28(10):826-834. doi:10.1136/bmjqs-2019-009383
19. Ferreira GE, Abdel-Shaheed C, Underwood M, et al. Efficacy, safety, and tolerability of antidepressants for pain in adults: overview of systematic reviews. *BMJ*. 2023;380:e072415. doi:10.1136/bmj-2022-072415
20. Interian A, Martinez IE, Guarnaccia PJ, Vega WA, Escobar JI. A qualitative analysis of the perception of stigma among Latinos receiving antidepressants. *Psychiatr Serv*. Dec 2007;58(12):1591-4. doi:10.1176/ps.2007.58.12.1591
21. Mathieson S, Lin CC, Underwood M, Eldabe S. Pregabalin and gabapentin for pain. *BMJ*. Apr 28 2020;369:m1315. doi:10.1136/bmj.m1315
22. Mathieson S, O'Keeffe M, Traeger AC, Ferreira GE, Abdel Shaheed C. Content and sentiment analysis of gabapentinoid-related tweets: An infodemiology study. https://doi.org/10.1111/dar.13590. *Drug and Alcohol Review*. 2022/12/20 2022;n/a(n/a)doi:https://doi.org/10.1111/dar.13590
23. Therapeutic. Therapeutic Goods Administration. Pregabalin and gabapentin. Safety advisory - enhanced warnings relating to abuse and dependence. https://www.tga.gov.au/news/safety-alerts/pregabalin-and-gabapentin
24. Tamblyn R, Berkson L, Dauphinee WD, et al. Unnecessary prescribing of NSAIDs and the management of NSAID-related gastropathy in medical practice. *Ann Intern Med*. Sep 15 1997;127(6):429-38. doi:10.7326/0003-4819-127-6-199709150-00003
25. Campbell SM, Hann M, Hacker J, et al. Identifying predictors of high quality care in English general practice: observational study. *BMJ*. 2001;323(7316):784. doi:10.1136/bmj.323.7316.784
26. Tsiga E, Panagopoulou E, Sevdalis N, Montgomery A, Benos A. The influence of time pressure on adherence to guidelines in primary care: an experimental study. *BMJ Open*. 2013;3(4):e002700. doi:10.1136/bmjopen-2013-002700
27. Privett N, Guerrier S. Estimation of the Time Needed to Deliver the 2020 USPSTF Preventive Care Recommendations in Primary Care. *Am J Public Health*. Jan 2021;111(1):145-149. doi:10.2105/ajph.2020.305967
28. Porter J, Boyd C, Skandari MR, Laiteerapong N. Revisiting the Time Needed to Provide Adult Primary Care. *J Gen Intern Med*. Jan 2023;38(1):147-155. doi:10.1007/s11606-022-07707-x
29. Bardin LD, King P, Maher CG. Diagnostic triage for low back pain: a practical approach for primary care. *Med J Aust*. Apr 3 2017;206(6):268-273. doi:10.5694/mja16.00828
30. Gabbay J, le May A. Evidence based guidelines or collectively constructed "mindlines?" Ethnographic study of knowledge management in primary care. *Bmj*. Oct 30 2004;329(7473):1013. doi:10.1136/bmj.329.7473.1013
31. Kasch R, Truthmann J, Hancock MJ, et al. Association of Lumbar MRI Findings with Current and Future Back Pain in a Population-based Cohort Study. *Spine*. 2022;47(3)
32. Australian Commission on Safety and Quality in Health Care.

Low Back Pain Clinical Care Standard. Sydney: ACSQHC; 2022.
33. Flodgren G, O'Brien MA, Parmelli E, Grimshaw JM. Local opinion leaders: effects on professional practice and healthcare outcomes. *Cochrane Database Syst Rev*. Jun 24 2019;6(6):Cd000125. doi:10.1002/14651858.CD000125.pub5

For peer review only - http://bmjopen.bmj.com/site/about/guidelines.xhtml

34. Evans SC, Roberts MC, Keeley JW, et al. Vignette methodologies for studying clinicians' decision-making: Validity, utility, and application in ICD-11 field studies. *Int J Clin Health Psychol*. May-Aug 2015;15(2):160-170. doi:10.1016/j.ijchp.2014.12.001
35. Alexander CS, Becker HJ. The Use of Vignettes in Survey Research. *Public Opinion Quarterly*. 1978;42(1):93-104. doi:10.1086/268432

Table 1. Features of each clinical vignette.

	Vignette 1	Vignette 2	Vignette 3
**Patient characteristics**			
Female	X		X
Male		X	
Middle-aged	X	X	X
Physical activity			
No physical activity	X		
Insufficient physical activity			X
Physically active		X	
**Social aspects**			
Children			X
Primary carer for older person	X		
**Type of low back pain**			
Atraumatic back pain	X	X	X
Back pain initiated after a well-defined event		X	
Back pain with a clear radicular component		X	
Back pain with referred leg pain			X
**Pain characteristics**			
Night pain	X	X	
Sleep disturbance	X		
Back pain exacerbated by movements/postures		X	X
Acute-on-chronic pain	X		
Sub-acute (pain between 6 and 12 weeks)		X	
Chronic (pain > 12 weeks)			X
Movement restriction	X	X	
Limitations to work		X	
**Comorbidities**			
Obesity	X		
Gastro-oesophageal reflux disease	X		
High cholesterol	X		
Other musculoskeletal conditions	X		
Diabetes			X
Depression (mild)			X
**Healthcare use**			
General practitioner for low back pain		X	
Physiotherapy for low back pain			X
Acupuncture for low back pain			X
Psychologist for mild depression			X
Previous imaging	X		
Previous/current use of pain medicines	X	X	X
Paracetamol, NSAIDs	X		X

For peer review only - http://bmjopen.bmj.com/site/about/guidelines.xhtml

Opioids		X	

For peer review only

For peer review only - http://bmjopen.bmj.com/site/about/guidelines.xhtml

Table 2. Characteristics of participants

Age, mean (SD)	53.4 (9.1)
Sex, n (%)	
Female	9 (36)
Male	16 (64)
State/territory	
New South Wales	9 (36)
Queensland	6 (24)
Victoria	5 (20)
South Australia	2 (8)
Western Australia	2 (8)
Tasmania	1 (4)
Remoteness	
Metropolitan	22 (88)
Rural	3 (12)
Number of patients with low back pain seen per year, n (%)	
5-25	2 (8)
26-50	4 (16)
51-100	3 (12)
>100	16 (64)
Years of experience, n (SD)	24.6 (9.3)
Weekly clinical workload (hours), mean (SD)	38 (7.7)

For peer review only

For peer review only - http://bmjopen.bmj.com/site/about/guidelines.xhtml

Table 3. Frequency of pain medicines that GPs would prescribe, would conditionally prescribe, were against prescribing, or that were not mentioned for each vignette.

	Paracetamol	NSAIDs	Opioids	Antidepressants	Anticonvulsants	Oral steroids	Benzodiazepines
**Vignette 1 (n = 25)**							
Would prescribe	13	17	8	6	1	0	0
Would conditionally prescribe	0	3	14	3	0	1	3
Against prescribing	0	2	2	6	9	1	1
Not mentioned	12	3	1	10	15	23	21
**Vignette 2 (n = 25)**							
Would prescribe	8	16	7	6	11	3	0
Would conditionally prescribe	0	0	12	7	5	2	0
Against prescribing	0	2	6	3	5	3	1
Not mentioned	17	7	0	9	4	17	24
**Vignette 3 (n = 24)***							
Would prescribe	8	8	8	10	5	0	1
Would conditionally prescribe	1	1	3	5	2	0	0
Against prescribing	0	2	4	0	1	2	0
Not mentioned	15	13	9	9	16	22	23

GP, General practitioner; NSAIDs, non-steroidal anti-inflammatories
*Data from 24 GPs shown. The interview with one GP discussing vignette 3 was not recorded due to technical issues.

For peer review only - http://bmjopen.bmj.com/site/about/guidelines.xhtml

Table 4. Themes, subthemes, and supporting quotes.

Themes	Subthemes	Supporting quotes
Prioritising personal information sources	Previous experience	• As an experienced GP of just over 20 years, you have scripts in your mind that bring you to a sort of automatic pilot approach. (GP #20, male, 21 years of experience)<br>• I'd probably just give her the options of weather she wants some anti-inflammatory, something with codeine, and antidepressant. (GP #3, female, 23 years of experience)<br>• I've had radicular pain myself, and the only thing that fixed me was Lyrica. I actually had little faith in Lyrica before I used it, but nothing else touched my pain. A lot of my approach to radicular pain is based on my own experience. (GP #1, female, 30 years of experience)
	Guidelines are unhelpful	• Sometimes I find guidelines are not actually practical. If they say 'oh tell the patient they need to keep fit and tell them that pain is not a bad sign and that they should keep active and a little bit of pain is ok', it can be hard to convince patients of that. (GP #1, female, 30 years of experience)
	Clinical training	• Probably what I've heard at specialist meetings when the specialists have spoken. I went to one last night and the neurosurgeon was basically saying you try and avoid going on the path of long-acting opioids, and you can use a quick acting opioid. (GP #6, female, 35 years of experience).<br>• Part of it is what we what we've learned through GP registrar training and medical school, where there's a strong emphasis on not using opioid painkillers in for the management of back pain (GP #17, male, 5 years of experience)
Strong reliance on a biomedical model to guide prescription	Pathoanatomical diagnosis	• When we do the imaging, it's going to support our treatment more. If you find one of two nerves there affecting him, we can start him on pregabalin 25 mg (GP #23, female, 28 years of experience)<br>• If we found multiple levels of a minor disc bulge or something, I would be happier to give her something stronger, but if the MRI just showed that she had a disc bulge at one level and everything else looks fine, there's no nerve impingement, I don't think that would warrant it. I'd be happy to try amitriptyline than other medications as a first line. (GP #9, female, 25 years of experience).
	Sleep patterns	• I'd be happy to give her some panadeine [NB: codeine + paracetamol] straight away because it's keeping her awake at night, and that's always a bit of a problem. She wakes up because of the pain… I mean you don't want people waking up with pain, so give her a bit of mild pain relief. (GP #22, male, 39 years of experience)
	Pain severity	• No because she might still respond to what I've decided to use, it might just be temporary that she's eight or nine [NB: pain out of 10] (GP #4, female, 30 years of experience)
	Comorbidities	• There are some other antidepressants like Cymbalta which can act as a neuropathic agent as well as an antidepressant. So there are antidepressants which we can use which help patients manage their depression as well as well as their pain. (GP #19, male, 30 years of experience)
Social factors as root causes	pain medicines do not address the root cause of the problem	• We don't want her having a fall with her mum and we don't want her experiencing side effects that may affect her ability to care for her mother. (GP #7, male, 18 years of experience)
	Psychosocial-based prescribing	• Since she's getting relief with paracetamol and ibuprofen, I think the next step is to find if there are any associated issues in terms of looking after her mum. Can we get some home help… refer mum to aged care services? I mean she might be able to get NDIS help [NB: government support] as well (GP #11, male, 20 years of experience)

For peer review only - http://bmjopen.bmj.com/site/about/guidelines.xhtml

Judicious use of pain medicines	Keep it simple or make it complex?	• They get flare ups here and there and I think you don't jump up and down as long as you know exactly the origin of the pain and you know you excluded that there is more to it than just what's presenting at this stage, and I think let's just keep it simple. And you can always go back and increase the medication or order further investigations if things don't subside, but I don't hit them harder on the 1st presentation. (GP #15, male, 30 years of experience)<br><br>• I tend to like to use a smaller dose of a lot of things rather than a bigger dose of one or two things. So I'd write a little plan and it would have a sliding scale so it would start off with the Panadol three times a day plus the Nurofen. That's the foundations of the house, and then I would add into that tramadol sustained release 150 to 100 twice a day, and then I would usually add in amitriptyline 10 to 20 milligrams before bed. (GP #25, female, 30 years of experience)
Concerns about prescription	drug seeking behaviour and risk of long-term misuse	• It's a chronic, insidious, ongoing bit of depression. And it just typically… It's just super clean. She gets started on endone and then get stuck on endone and that becomes their whole life. And they just slowly over a course of years dose-escalate. And they feel better. Because of the euphoria associated with the oxycodone. Terrible. (GP #16, male, 34 years of clinical experience)
	Side effects of prescription pain medicines	• Opioids induce hyperalgesia, which comes with a lot of opioids such as codeine, which is a poor analgesic. I don't use that either. I basically use 3 analgesics in my practice: tramadol, buprenorphine, and tapentadol. (GP #19, Male, 30 years of experience)
	Stigma around prescription	• It could be done, but it would involve a very lengthy consultation and we as GPs don't have half an hour to 45 minutes to discuss something a little bit more wayward. (GP #7, male, 18 years of experience)
	Pressure from regulators	• The guidelines at the moment for non-opioid medication are clear as far as we can all see. I don't know how much that'll go the wrong way and we'll end up not treating anybody pains eventually. We get sued for not treating anybody pain and the pain and suffering and next thing you know the guidelines will change, you know? But you can't win, can you? (GP #14, male, 40 years of experience)<br><br>• Department of Health want us not to give pain medicine at all for low back pain but sometimes it's not realistic. Sometimes we need to help the patient even though I mean, potentially they can be additive of course, but, realistically, the patient is in so much pain, they can't move. This is cruel. Not giving opioid medicines, at least for a few days. I'm not the type of person that gives everybody strong pain medicines, but I think there is a role in some patients. (GP #18, male, 27 years of experience)

For peer review only - http://bmjopen.bmj.com/site/about/guidelines.xhtml

For peer review only

General practitioners' decision-making process to prescribe pain medicines for low back pain: a qualitative study

Contents

Supplementary file 1. Vignettes..2
Supplementary file 2. Interview guide...5

For peer review only

Supplementary file 1. Vignettes

Prompt: I will now present to you three clinical vignettes describing different low back pain presentations. After each vignette I am going to ask you a series of questions. Please draw on your prior experience to answer the questions about how you would manage these back pain patients. There are no right or wrong answers. We are interested in honest opinions, not idealised ones (for example, what you should do). Remember that your data will be anonymised.

VIGNETTE 1

- Patient info: Female patient, 57 years old, BMI 33 (height: 1.60m; weight: 85kg)
- Complaint: atraumatic onset low back pain that started 2 weeks ago. Average pain 5/10; worse in the evening/at night with pain 7/10.
- History:
  - Low back pain for 20+ years (ongoing mild pain 2/10) with occasional moderate flares (pain up to 5/10) that typically resolve in a few days. Previous MRIs (last one 3 years ago) showed the following: At L5/S1 there is vertebral endplate bone marrow oedema. The distinction between the nucleus and annulus is lost with a with loss of disc height.
  - She has sought care for back pain a few times before, but typically self-manages the pain with simple painkillers (Paracetamol and occasionally ibuprofen as needed). She is seeking care for this episode because it's taking longer to resolve, and the pain is more intense in the evening/at night
  - Patient is having difficulty sleeping – she wakes up because of pain when turning in bed.
  - Patient is sedentary and is the primary carer of her 88-year-old mother who is bed-bound due to a stroke 12 months ago.
  - Comorbidities: Obesity, GORD, high cholesterol (daily atorvastatin), medial compartment R knee osteoarthritis (also treats with simple analgesics as needed).
- Physical examination:
  - No evidence of radicular symptoms: straight leg raise, sensation, reflexes, and lower limb strength are normal. No signs of infection or midline tenderness in the lumbar spine. Tenderness over the paraspinal muscles is present.
  - Movements in every direction are slightly more painful (6/10)

For peer review only

VIGNETTE 2

- Patient info: 45-year-old male, BMI 26 (height: 1.83m; weight: 87kg)

- Complaint: Acute onset low back pain with shooting stabbing pain radiating down the back of the right leg to the foot and associated with pins and needles in the same area. This commenced 8 weeks ago after trying to lift a heavy box while visiting family in rural NSW. Main complaint is leg pain (5/10), but he also has pain in the lower back (3/10). Sitting for >1h aggravates leg pain (7/10), sit-to-standing and vice-versa are quite painful (7/10).

- History:

  - No history of low back pain or any other musculoskeletal condition prior to this episode. Patient is physically active, swims and runs twice a week. Sitting for more than 1 hour aggravates pain and is severely limiting his ability to work. He has not taken any time off work.
  - Patient went to the local general practitioner the day after pain onset who prescribed codeine 30mg QID for 1 week. No improvement was noted. No significant side effects were noted either besides constipation.
  - Patient has not reported any significant sleep disturbance

- Physical examination:
  - Pain follows a dermatomal distribution (S1), with altered sensation to light touch. Muscle strength and reflexes are preserved.
  - No signs of infection or midline tenderness in the lumbar spine.
  - Straight leg raise positive at ~30deg; crossed straight leg raise test is negative
  - Flexion and extension movements are limited and increase leg pain (7/10). Rotations and inclinations do not change symptoms.
  - Cauda equina syndrome and Other red flags were excluded

For peer review only

VIGNETTE 3

- Patient info: Female patient, 52 years old, BMI 30 (height: 1.63m; weight: 79kg)

- Complaint: Insidious onset low back pain radiating to the L thigh that started 6 months ago. Patient cannot recall an event that triggered the pain. Pain is bilateral in the lower back and radiates to the L posterior thigh with the same average intensity (5/10). Standing up/walking for too long (>1h) increases pain (6-7/10). Sitting/lying down reduces pain. She does not have trouble sleeping.

- History:
  - No history of low back pain or any other musculoskeletal condition prior to this episode. Patient is married with 3 children (youngest is 20 years-old),
  - Patient has recently been diagnosed with type II diabetes (she takes metformin daily and has been going to the gym 3 times/week for about 45 minutes where she does low-moderate intensity resistance training since the diagnosis – 2 years ago. Her sugar levels are currently controlled – around 5mmol/L before meals. Kidney function is normal). She was diagnosed with mild depression two years ago and has been supported by a psychologist with training in cognitive behavioural therapy. No other pharmacological treatments are used for depression.
  - She uses over-the-counter analgesics (Paracetamol and Ibuprofen) regularly on an as-needed basis to manage the pain. She has seen a physiotherapist (core strengthening exercises) and an acupuncturist but has not responded to either treatment.

- Physical examination:
  - No abnormalities on neurological exam (sensation, strength, and reflexes are all normal).
  - No signs of infection or midline tenderness in the lumbar spine.
  - Straight leg raise and crossed Straight leg raise negative.
  - Lumbar spine movements are limited mostly by lack of flexibility. End-range lumbar extension increases pain momentarily (7/10), which reduces as soon as patient returns to neutral position.

For peer review only

Supplementary file 2. Interview guide.

Questions - Choosing between different types of pharmacological treatments

Prompt: Answer the following questions based on the patient vignette you just read. Using the information from the vignette, I would like you to think aloud your clinical reasoning.

- Would you typically prescribe a pain medicine for the patient described in the vignette?

- Could you elaborate on which pain medicines you would prescribe and why you would prescribe them *[remind the participant of the medicines they mentioned]*?

Prompt participant to talk about their rationale to prescribe/recommend certain medicines; what kind of thought process they follow, sources of information that they use to make their decision:

- *Clinical presentation (e.g. affected sleep in vignette 1; neuropathic pain features in vignette 2; depression in vignette 3, etc.)*
- *Severity of symptoms*
- *Known efficacy from trials and systematic reviews*
- *Guideline recommendation*
- *Safety/side effects*
- *Previous use by patient*
- *Previous success with other patients*
- *Demographic factors*
- *Mechanisms of action of the drug*
- *Intuition*

Questions – influence of factors not mentioned by the participant

- How do aspects like (eg age, gender, comorbidities, description of pain, etc.) factor in your decision to prescribe 'ABC' *[the pain medicines mentioned by the participant]*?

Questions - Alternatives

- What about pain medicines 'XYZ'? Would you prescribe any of these for the vignette? *[mention pain medicines that the participant did not mention eg strong opioids [oxycodone endone], weak opioids [codeine], gabapentin, pregabalin, amitriptyline, oral steroids, etc.)]*

- Are there any circumstances where you would prescribe XYZ instead or/in addition to ABC for the patient in this vignette?

Questions – external factors influencing decision-making

- Would your decision-making change if you were pressed for time? *[eg do they report using any heuristic/rules of thumb when they are time-limited?]*
- Would your decision-making change depending on patients' opinions on the treatments you typically prescribe?

For peer review only

COREQ (COnsolidated criteria for REporting Qualitative research) Checklist

A checklist of items that should be included in reports of qualitative research. You must report the page number in your manuscript where you consider each of the items listed in this checklist. If you have not included this information, either revise your manuscript accordingly before submitting or note N/A.

Topic	Item No.	Guide Questions/Description	Reported on Page No.
**Domain 1: Research team and reflexivity**			
Personal characteristics			
Interviewer/facilitator	1	Which author/s conducted the interview or focus group?	
Credentials	2	What were the researcher's credentials? E.g. PhD, MD	
Occupation	3	What was their occupation at the time of the study?	
Gender	4	Was the researcher male or female?	
Experience and training	5	What experience or training did the researcher have?	
Relationship with participants			
Relationship established	6	Was a relationship established prior to study commencement?	
Participant knowledge of the interviewer	7	What did the participants know about the researcher? e.g. personal goals, reasons for doing the research	
Interviewer characteristics	8	What characteristics were reported about the inter viewer/facilitator? e.g. Bias, assumptions, reasons and interests in the research topic	
**Domain 2: Study design**			
Theoretical framework			
Methodological orientation and Theory	9	What methodological orientation was stated to underpin the study? e.g. grounded theory, discourse analysis, ethnography, phenomenology, content analysis	
Participant selection			
Sampling	10	How were participants selected? e.g. purposive, convenience, consecutive, snowball	
Method of approach	11	How were participants approached? e.g. face-to-face, telephone, mail, email	
Sample size	12	How many participants were in the study?	
Non-participation	13	How many people refused to participate or dropped out? Reasons?	
Setting			
Setting of data collection	14	Where was the data collected? e.g. home, clinic, workplace	
Presence of non-participants	15	Was anyone else present besides the participants and researchers?	
Description of sample	16	What are the important characteristics of the sample? e.g. demographic data, date	
Data collection			
Interview guide	17	Were questions, prompts, guides provided by the authors? Was it pilot tested?	
Repeat interviews	18	Were repeat inter views carried out? If yes, how many?	
Audio/visual recording	19	Did the research use audio or visual recording to collect the data?	
Field notes	20	Were field notes made during and/or after the inter view or focus group?	
Duration	21	What was the duration of the inter views or focus group?	
Data saturation	22	Was data saturation discussed?	
Transcripts returned	23	Were transcripts returned to participants for comment and/or	

For peer review only

Topic	Item No.	Guide Questions/Description	Reported on Page No.
		correction?	
**Domain 3: analysis and findings**			
Data analysis			
Number of data coders	24	How many data coders coded the data?	
Description of the coding tree	25	Did authors provide a description of the coding tree?	
Derivation of themes	26	Were themes identified in advance or derived from the data?	
Software	27	What software, if applicable, was used to manage the data?	
Participant checking	28	Did participants provide feedback on the findings?	
Reporting			
Quotations presented	29	Were participant quotations presented to illustrate the themes/findings? Was each quotation identified? e.g. participant number	
Data and findings consistent	30	Was there consistency between the data presented and the findings?	
Clarity of major themes	31	Were major themes clearly presented in the findings?	
Clarity of minor themes	32	Is there a description of diverse cases or discussion of minor themes?	

Developed from: Tong A, Sainsbury P, Craig J. Consolidated criteria for reporting qualitative research (COREQ): a 32-item checklist for interviews and focus groups. *International Journal for Quality in Health Care*. 2007. Volume 19, Number 6: pp. 349 – 357

Once you have completed this checklist, please save a copy and upload it as part of your submission. DO NOT include this checklist as part of the main manuscript document. It must be uploaded as a separate file.

For peer review only

BMJ Open

General practitioners’ decision-making process to prescribe pain medicines for low back pain: a qualitative study

Journal:	*BMJ Open*
Manuscript ID	bmjopen-2023-074380.R1
Article Type:	Original research
Date Submitted by the Author:	21-Aug-2023
Complete List of Authors:	Ferreira, Giovanni; University of Sydney School of Public Health, Institute for Musculoskeletal Health<br>Zadro, Joshua; University of Sydney, Institute for Musculoskeletal Health, School of Public Health<br>Jones, Caitlin; The University of Sydney Institute for Musculoskeletal Health, School of Public Health<br>Ayre, Julie; The University of Sydney, Sydney Health Literacy Lab<br>Lin, Christine; University of Sydney School of Public Health, Institute for Musculoskeletal Health<br>Richards, Bethan; The University of Sydney Institute for Musculoskeletal Health<br>Needs, Christopher; Institute of Rheumatology & Orthopaedics, Rheumatology Department<br>Abdel Shaheed, Christina; University of Sydney School of Public Health, Institute for Musculoskeletal Health<br>McLachlan, Andrew; University of Sydney, Faculty of Pharmacy<br>Day, Richard; St Vincent's Hospital, Department of Clinical Pharmacology & Toxicology; University of New South Wales, St Vincent's Clinical School, Faculty of Medicine<br>Maher, Christopher; University of Sydney, Institute for Musculoskeletal Health, Sydney School of Public Health, Faculty of Medicine and Health
**Primary Subject Heading**:	General practice / Family practice
Secondary Subject Heading:	Qualitative research
Keywords:	Decision Making, Back pain < ORTHOPAEDIC & TRAUMA SURGERY, QUALITATIVE RESEARCH

SCHOLARONE™
Manuscripts

I, the Submitting Author has the right to grant and does grant on behalf of all authors of the Work (as defined in the below author licence), an exclusive licence and/or a non-exclusive licence for contributions from authors who are: i) UK Crown employees; ii) where BMJ has agreed a CC-BY licence shall apply, and/or iii) in accordance with the terms applicable for US Federal Government officers or employees acting as part of their official duties; on a worldwide, perpetual, irrevocable, royalty-free basis to BMJ Publishing Group Ltd ("BMJ") its licensees and where the relevant Journal is co-owned by BMJ to the co-owners of the Journal, to publish the Work in this journal and any other BMJ products and to exploit all rights, as set out in our licence.

The Submitting Author accepts and understands that any supply made under these terms is made by BMJ to the Submitting Author unless you are acting as an employee on behalf of your employer or a postgraduate student of an affiliated institution which is paying any applicable article publishing charge ("APC") for Open Access articles. Where the Submitting Author wishes to make the Work available on an Open Access basis (and intends to pay the relevant APC), the terms of reuse of such Open Access shall be governed by a Creative Commons licence – details of these licences and which Creative Commons licence will apply to this Work are set out in our licence referred to above.

Other than as permitted in any relevant BMJ Author's Self Archiving Policies, I confirm this Work has not been accepted for publication elsewhere, is not being considered for publication elsewhere and does not duplicate material already published. I confirm all authors consent to publication of this Work and authorise the granting of this licence.

For peer review only

General practitioners' decision-making process to prescribe pain medicines for low back pain: a qualitative study

Giovanni E Ferreira[1,2], Joshua R Zadro[1,2], Caitlin Jones[1,2], Julie Ayre[1,2], Chung-Wei Christine Lin[1,2], Bethan Richards[2,4], Christopher Needs[4], Christina Abdel Shaheed[1,2], Andrew McLachlan[5], Richard Day[6], Chris G Maher[1,2]

1. Sydney Musculoskeletal Health, Faculty of Medicine and Health, The University of Sydney
2. Institute for Musculoskeletal Health, The University of Sydney and Sydney Local Health District
3. Sydney Health Literacy Lab, School of Public Health, Faculty of Medicine and Health, The University of Sydney, Sydney, Australia
4. Department of Rheumatology, Royal Prince Alfred Hospital, Sydney, Australia
5. Sydney Pharmacy School, Faculty of Medicine and Health, The University of Sydney, Sydney, Australia
6. St Vincent's Clinical School, Department of Clinical Pharmacology & Toxicology, University of New South Wales, Sydney, Australia

For peer review only

Abstract word count: 291
Manuscript word count: 3989

Address all correspondence to:

Giovanni E. Ferreira
School of Public Health, Faculty of Medicine and Health, The University of Sydney, Sydney, New South Wales, Australia
PO Box M179, Missenden Road, Camperdown | NSW | 2050, Australia
E-mail: giovanni.ferreira@sydney.edu.au

For peer review only - http://bmjopen.bmj.com/site/about/guidelines.xhtml

ABSTRACT

Background: Pain medicines are widely prescribed by general practitioners (GPs) when managing people with low back pain (LBP), but little is known about what drives decisions to prescribe these medicines.

Objectives: The aim of this study was to investigate what influences GPs' decision to prescribe pain medicines for LBP.

Design: Qualitative study with in-depth interviews.

Setting: Australian primary care.

Participants: We interviewed 25 GPs practising in Australia experienced in managing LBP (mean [SD] age 53.4 [9.1] years, mean [SD] years of experience: 24.6 [9.3], 36% female). GPs were provided three vignettes describing common LBP presentations (acute exacerbation of chronic LBP, subacute sciatica, and chronic LBP) and were asked to think aloud how they would manage the cases described in the vignettes.

Data analysis: We summarised GP's choices of pain medicines for each vignette using content analysis and used framework analysis to investigate factors that affected GP's decision-making.

Results: GPs more commonly prescribed opioid analgesics. Anticonvulsants and antidepressants were also commonly prescribed depending on the presentation described in the vignette. GP participants made decisions about what pain medicines to prescribe for LBP largely based on previous experiences, including their own personal experiences of LBP, rather than guidelines. The choice of pain medicine was influenced by a range of clinical factors, more commonly the patient's pathoanatomical diagnosis. While many adhered to principles of judicious use of pain medicines, polypharmacy scenarios were also common. Concerns about drug seeking behaviour, adverse effects, stigma around opioid analgesics, and pressure from regulators also shaped their decision-making process.

Conclusions: We identified several aspects of decision-making that help explain the current profile of pain medicines prescribed for LBP by GPs. Themes identified by our study could inform future implementation strategies to improve the quality use of medicines for LBP.

Keywords: decision-making, opioids, low back pain, prescription, sciatica

For peer review only - http://bmjopen.bmj.com/site/about/guidelines.xhtml

Strengths and limitations of the study

- We used clinical vignettes resembling common low back pain cases to provide clinicians with a concrete starting point where they could base their decisions on
- Voices and perspectives of younger general practitioners or general practitioners working in rural and remote areas may have been underrepresented.

For peer review only

INTRODUCTION

Low back pain (LBP) affects over 4 million Australians every year and pain medicines are widely used in its management.(1) Australian data shows that 2 in every 3 patients who seek primary care are prescribed or recommended at least one pain medicine.(2) Examples include over-the-counter analgesics (e.g. paracetamol, and non-steroidal anti-inflammatories (NSAIDs)) and prescription medicines such as opioid analgesics, gabapentinoids, antidepressants, and muscle relaxants.(3)

There is a mismatch between the pharmacological therapies recommended in clinical practice guidelines for LBP and what is prescribed or recommended to patients in primary care. For example, opioid analgesics are one of the most commonly prescribed medicines for people presenting with LBP across various settings and their use has increased 39% in the last decade.(3) This is despite guidelines discouraging their use for LBP when pain is not severe or as first-line care before trialling other treatments.(3, 4) Similarly, gabapentinoids are ineffective and potentially harmful for the management of LBP; however their use is increasing.(4-6) On the other hand, the use of pharmacological therapies that are recommended as first line care such as NSAIDs has reduced 33% from 2004 to 2014.(3) In the United States, antidepressants are prescribed to about a quarter of patients with LBP.(7) In Portugal, 1 in 7 people with chronic LBP report using antidepressants and benzodiazepines for pain management.(8) However, LBP guidelines recommend against both medicines.(4)

In Australia, LBP is the sixth most commonly managed condition overall and the most common musculoskeletal condition managed by general practitioners (GPs).(9) A UK-based study provided some insight into aspects that shape the decision-making process of health professionals, including GPs. That study described how clinicians select treatments based upon knowing the individual patient, the patient's LBP, and common treatment options.(10) However, this previous study did not explore the decision-making processes that influence a GP's decision to prescribe pain medicines for LBP in-depth, particularly in relation to the processes that underpin the choice for different pain medicines. Therefore, the aim of this qualitative study was to investigate what influences GPs' decision-making process for prescribing pain medicines for people with LBP.

METHODS

Design

This qualitative study was approved by XXXXX Human Research Ethics Committee (approval number: 2022/170) and is reported per the COREQ statement.(11)

Participant selection and recruitment

We recruited 25 GPs registered to practise in Australia who reported having provided care for at least five patients with LBP with or without sciatica in the last 12 months. Four or less is what our team thought would be representative of little to no experience in managing LBP. We defined sciatica as a back problem with radiating pain below the knee with or without neurological symptoms (e.g. reduced muscle strength, sensation, or reflexes).(12)

For peer review only - http://bmjopen.bmj.com/site/about/guidelines.xhtml

GPs were recruited via a market research company (TKW Research Group, Australia) with a large database of healthcare professionals across Australia and New Zealand. Health professionals opt into this database and therefore have already indicated a willingness to participate in research. None of the participants had a prior relationship with the study investigators. The company sent out emails to GPs in their database and scheduled interviews with those who expressed interest in the study.

Interviews and data collection

We conducted in-depth, one-on-one semi-structured interviews. Interviews were conducted by videoconference between May and June 2022, audio recorded, and transcribed verbatim. A researcher with a background in occupational therapy and experience in qualitative interviews and pain management research conducted the interviews.

An interview guide and three clinical vignettes to guide the interviews were developed by the lead author, a physiotherapist with experience in LBP research and pain management, and two experienced rheumatologists who provide care for patients with LBP (supplementary file 1). The vignettes were designed to represent three common LBP presentations: a patient with an acute exacerbation of chronic LBP, a patient with subacute sciatica (pain duration between 6-12 weeks), and a patient with chronic non-specific LBP (pain for longer than 12 weeks). Collectively, the vignettes were modelled to describe a range of factors that could play a role in a doctors' decision-making process to manage LBP (Table 1). GPs were asked to think aloud how they would manage the cases described in the vignettes.(13) For each clinical decision made by the participant, the researcher facilitating the interview asked participants to elaborate on a point, or to obtain clarification if needed (Supplementary file 2).

Analysis

Interview data were analysed using two approaches. We used content analysis to create a summary of GP's choices of pain medicines prescribed for each vignette. Content analysis combines both qualitative and quantitative methods, allowing both the content and frequency of categories to be reported.(14, 15) Two researchers initially reviewed and familiarised themselves with the transcripts, and coded all pain medicines using the following framework: medicines that would be prescribed as first, second, third, or fourth-line care, medicines that GPs would conditionally prescribe (e.g. depending on a patient characteristic), and those that GPs were against prescribing.

We used framework analysis to investigate factors that influence GP's decision-making process.(16) Two researchers coded 10 interview transcripts inductively, developed a library of codes in an iterative process, decided on a coding framework (comprising the themes) and applied it to all interviews. Codes, sub-themes, and themes were reviewed by us, and changes were made as stronger patterns linking codes to themes emerged from the data.(17) Both researchers have a background in physiotherapy and have published on the quality use of pain medicines.(18-20) Data saturation was reached (i.e. no new themes emerged).

Patient and public involvement

For peer review only - http://bmjopen.bmj.com/site/about/guidelines.xhtml

It was not appropriate or possible to involve patients or the public in the design, or conduct, or reporting, or dissemination plans of our research.

RESULTS

We interviewed 25 GPs from April to June, 2022. Most were males (64%) practising in metropolitan areas across six states of Australia. Their mean (SD) age was 53.4 (9.1), and they had a mean of 24.5 (9.3) years of practice as GPs. All but three worked full-time in private practices (Table 2). The recoding of one interview was mistakenly interrupted just before vignette #3 started and therefore there are only data from 24 GPs for that vignette.

Content analysis

Paracetamol and NSAIDs were commonly prescribed for all three vignettes. However, opioid analgesics were the pain medicines most prescribed or conditionally prescribed for the acute exacerbation of chronic LBP and sciatica vignettes, and antidepressants for the chronic LBP vignette. Codeine was the most prescribed opioid analgesic for the acute exacerbation vignette, whereas tramadol was the most common choice for the sciatica vignette. Concerns over the safety of opioids, particularly codeine, were commonly reported by GPs across all vignettes. Those concerns were often specific to a type of opioid, as many who expressed concerns over one type of opioid would still prescribe a different opioid analgesic or conditionally prescribe the same opioid. For example, this was observed in the interviews of 11 (44%) GPs in the acute exacerbation vignette (Table 3).

Anticonvulsants were the medicine class with the highest number of GPs indicating they would not prescribe for the acute exacerbation vignette: nine (36%) were against prescribing them because the patient did not have signs of neuropathic pain that would justify their use. In contrast, 16 (64%) of GPs recommended an anticonvulsant for the sciatica vignette, mostly pregabalin. GPs prescribed antidepressants for all three vignettes, more commonly for the chronic LBP vignette (n=15, 63%). Of those, most chose duloxetine (n=10, 42%) and/or amitriptyline (n=7, 29%). GPs prescribed antidepressants for different reasons for each vignette: co-morbid depression influenced decision-making for the chronic LBP vignette, whereas neuropathic pain was the key reason leading to antidepressants being prescribed for the sciatica vignette.

Factors influencing GP's decision-making to prescribe pain medicines for LBP

We identified 5 key themes that influenced GP's decision-making. Themes, subthemes, and supporting quotes are presented in table 4.

Theme 1: Prioritising personal information sources

Subtheme 1 - Previous experiences: Past clinical and personal experiences were recognised by most GPs as the most important source of information to help them make decisions. Some acknowledged that accumulated clinical experience contributed to the formulation of shortcuts that guided decision-making. Meaningful past experiences were often centred

For peer review only - http://bmjopen.bmj.com/site/about/guidelines.xhtml

around success using medicines with patients, but also personal success when they experienced pain themselves.

GPs considered patients' previous treatment experience an important source of information to assist with their decision-making. Giving patients options and discussing those options with them was often mentioned. However, in those circumstances, patient preferences and values appeared to be the key drivers of decision-making. Discussion about the evidence-base for each option was less prominent.

Subtheme 2 - Guidelines are unhelpful: Many GPs reported that guidelines were not helpful. Guidelines were noted to be particularly unhelpful when patients required more than minimal intervention such as advice and simple analgesics. Recommended interventions such as advice to stay active and education about the benign nature of most LBP were seen as problematic as it could be hard to convince patients in pain of their usefulness. One GP noted that the problem with guidelines was not necessarily their recommendations, but the time required to implement them in a context of GPs pressured for time. Clinical practice guidelines for LBP were not mentioned. Only the *Therapeutic Guidelines(21)* were mentioned by a limited number of GPs.

Subtheme 3 - Clinical training: While the applicability of guidelines was questioned, continuing education activities run by specialists and opinion leaders were highly valued. By comparison, training programs were rarely mentioned; only one GP with five years of experience mentioned the role of medical school and GP registrar training in addressing the current overuse use of opioids for LBP management.

Theme 2: Strong reliance on a biomedical model to guide prescription

Subtheme 1 - Pathoanatomical diagnosis: There was a strong sense amongst GPs that a pathoanatomical diagnosis was key to inform the choice of pain medicine to be prescribed. This was more pronounced for the chronic LBP vignette where, according to GPs, there was a more uncertain clinical presentation due to the description and duration of symptoms as well as comorbidities. Many GPs reported they would have referred the patient for imaging, and many considered the number and nature of imaging findings a key indicator of severity of the problem, which they linked to the prescription of stronger analgesics.

Subtheme 2 - Sleep patterns: Sleep influenced decision-making across the three vignettes, even though the patient in the chronic LBP vignette had no night pain or sleep disturbance. Improving sleep was an important treatment goal in the acute exacerbation vignette. For some, improving sleep was seen as the primary goal of treatment, and often led GPs to prescribe an opioid analgesics or antidepressants, or both. Advice on sleep hygiene was not mentioned.

Subtheme 3 - Pain severity: There were opposing views on the role of pain severity as a factor that influenced decision-making. While some were happy to consider stronger analgesics when pain was more severe, for others pain severity would not prompt immediate escalation to stronger analgesics. GPs were more inclined to consider escalating to stronger analgesics when discussing the acute exacerbation vignette and less inclined to do so in the

For peer review only - http://bmjopen.bmj.com/site/about/guidelines.xhtml

chronic LBP vignette (the average pain intensity was the same in both). For some GPs who would not consider escalating to stronger analgesics, their initial choice of treatment was typically a weak opioid analgesic such as codeine. Those preferring codeine mentioned being more used to the prescribing it than other opioid analgesics.

Subtheme 4 – Comorbidities: Comorbidities described in the vignettes were noted by most and influenced decision-making for some. Losing weight was often mentioned as a treatment goal in all three vignettes and led some GPs to preferring non-pharmacological approaches first or avoiding pharmacological treatments known to cause weight gain (e.g. antidepressants), despite obesity only being mentioned in the acute exacerbation vignette. The history of mild depression described in the chronic LBP vignette led many GPs to consider an antidepressant. The absence of depression in the subacute sciatica vignette was an important factor for GPs when deciding between different pharmacological options for neuropathic pain symptoms. Many acknowledged the role of antidepressants (eg duloxetine, amitriptyline) as potentially effective for symptoms described in the sciatica vignette, but most preferred anticonvulsants as the patient was young, otherwise healthy, physically active, and did not have chronic pain. In contrast, most GPs preferred to prescribe duloxetine for patients with comorbid depression and chronic pain such as the patient described in chronic LBP vignette.

Theme 3: Social factors as root causes

Subtheme 1 - Pain medicines do not address the root cause of the problem: Many GPs reported that prescribing medicines for pain for the acute exacerbation vignette would not be likely to address the root cause of the problem. Some suggested non-pharmacological treatments such as lifestyle interventions, but most GPs expressed concern about the level of social and family support that the patient had and its potential negative impact on her LBP.

Subtheme 2 - Psychosocial-based prescribing: Some GPs considered the patient’s social circumstances when deciding whether to prescribe a pain medicine and what to prescribe. In the acute exacerbation vignette, many thought that the patient’s social circumstances were important contributors to her pain but from different perspectives. While some emphasised the physical strain related to her carer role as a contributor to the pain, others recognised the psychological consequences of the patient’s social circumstances. Those who acknowledged physical factors preferred recommending non-pharmacological options such as ergonomic devices, whereas those who recognised the psychological toll of the patient’s carer role more commonly prescribed opioid analgesics or antidepressants. Other GPs were wary about whether side effects from opioid analgesics would hinder the patient’s ability to care for her mother due to potential side effects, such as sedation.

Theme 4: Judicious use of pain medicines

Subtheme 1 - Keep it simple or make it complex? There were opposing views about judicious use of pain medicines. Many GPs followed the principles of the WHO analgesic ladder and avoided polypharmacy, particularly for the acute exacerbation vignette, and, to a lesser extent, the sciatica vignette. For GPs following that approach, there was a perception that the clinical course of both vignettes would be favourable and keeping prescription of pain

For peer review only - http://bmjopen.bmj.com/site/about/guidelines.xhtml

medicines to a minimum, combined with non-pharmacological measures, would be effective. Chronic pain and the history of depression were seen as barriers to improvement in the chronic LBP vignette – and therefore to a perception that the patient with chronic LBP would have been more complex to manage.

Other GPs reported that they preferred prescribing multiple pain medicines in combination, at least in the short-term, and most commonly in the sciatica and chronic LBP vignettes. In the sciatica vignette, this was driven by two factors: the notion that there was a well-established mechanism of injury (as opposed to a degenerative process with no known trigger as in the acute exacerbation vignette), and the perception that the clinical presentation was more serious as the patient had radicular symptoms. Both factors led GPs to consider stronger pain medicines such as opioid analgesics and anticonvulsants. In the chronic LBP vignette, polypharmacy often included the prescription of simple analgesics with antidepressants, most commonly duloxetine, which was driven mainly by the history of mild depression.

Theme 5: Concerns about prescription

Subtheme 1 - Drug seeking behaviour and risk of long-term misuse: A key concern for many GPs was drug seeking behaviour. GPs used multiple strategies to identify drug seeking behaviours, such as a review of medical history and intuition. Some were reluctant to prescribe opioid analgesics to patients who were not their regular patients. Some GPs mentioned being able to identify certain stereotypes more likely to be drug seekers. Although opioids were commonly considered by GPs across the three vignettes, most had concerns about their potential for misuse and addiction, particularly in relation to the chronic LBP vignette. Chronic pain and a history of depression were seen as risk factors to long-term misuse and addiction to opioid analgesics.

Subtheme 2 - Side effects of prescription pain medicines: Similarly, most concerns about side effects were related to opioid analgesics. Many GPs expressed concerns about the safety profile of specific opioid analgesics, most commonly codeine, and made decisions about which opioid analgesic to use based on their knowledge about the safety profile of opioid analgesics (e.g., sedation, constipation, nausea). In contrast, very few GPs expressed concerns about other pain medicines with potential for misuse and abuse, such as pregabalin.

Subtheme 3 - Stigma around prescription: Some GPs were aware of the stigma around prescribing pain medicines such as antidepressants. Although frequently mentioned as a treatment option for the sciatica and chronic LBP vignettes, some had concerns that prescribing antidepressants had potential for misinterpretation by patients – for example, that they were implying that the patient's pain was psychological. Lack of time during a regular consultation was seen as important barrier for prescribing antidepressants. There was a perception that longer consultations would be required to explain why the medication was being prescribed to mitigate any concerns from patients.

Subtheme 4 - Pressure from regulators: Some GPs were concerned about being pressured by regulators to not prescribe pain medicines such as opioid analgesics. Their concerns included the potential consequences of undertreating pain. No GP mentioned that pressure from regulators had changed their prescribing behaviour.

For peer review only - http://bmjopen.bmj.com/site/about/guidelines.xhtml

DISCUSSION

GPs in this study made decisions about what pain medicines to prescribe for LBP largely based on previous experiences, including their own clinical and personal experience of LBP management, not guidelines. The choice of pain medicine was influenced by a range of clinical factors, more commonly the patient's pathoanatomical diagnosis. While many adhered to principles of pain medicines prescribing, polypharmacy scenarios were also common. Concerns about drug seeking behaviour, side effects, and stigma around prescription also shaped GPs' decision-making process.

Antidepressants were considered by GPs in our study when they thought depression was linked to the patient's symptoms or as a pain medicine to treat neuropathic pain. Although there is some evidence that antidepressants may be effective for those with chronic pain and comorbid depression and neuropathic pain,(20) the role of antidepressants for chronic LBP is limited and the evidence for sciatica is inconclusive.(18) Some GPs voiced concerns about stigma around prescribing antidepressants for pain that was not observed for any other pain medicine in our study. Stigma around the use of antidepressants is a well-recognised phenomenon in the mental health field, where studies have shown that people using antidepressants for depression have a perception that their condition is more severe and that they are weak or unable to cope with their problems.(22) GPs in our study had the perception that patients may not receive the idea of being prescribed an antidepressant for their pain well.

While our GPs were aware of the harms and potential for misuse and abuse of opioid analgesics, fewer mentioned concerns with other prescription pain medicines such as anticonvulsants, which also have potential for misuse, addiction, and death.(23) The apparent lack of awareness about the harms of these medicines appears to also be common amongst the public.(24) In contrast, public health entities and government regulatory agencies have recognised the concerning potential harms of these medicines and have taken action to reduce them. Such concerns have led regulatory agencies in the UK and Australia to up schedule and to add Boxed Warnings to the Product Information and Consumer Medicine Information for pregabalin, respectively.(23, 25)

Time constraints in general practice are linked to poor adherence to guidelines, inappropriate prescription, and provision of worse care.(26-28) Furthermore, meeting guideline recommendations in primary care requires an unrealistic amount of time that GPs do not have.(29) In line with these findings, there was a perception amongst some GPs that the time required to implement guideline recommendations into practice, or to discuss prescription of certain pain medicines (e.g. antidepressants), was an important barrier to their prescription. Several studies have pointed out that GPs perceive it as nearly impossible to meet all guideline recommendations as there are too many recommendations and most are complex. For example, implementing and documenting all recommendations from guidelines for common problems seen in general practice would take up to 27 hours per day.(30) LBP guidelines involve a complex series of steps from triage to management, and may also take considerable time to be implemented.(31)

For peer review only - http://bmjopen.bmj.com/site/about/guidelines.xhtml

Our findings provide insights for future research to improve the quality use of medicines for LBP. For example, GPs in our study rarely made decisions based on guidelines or evidence. Rather, they strongly relied on previous personal experience with pain medications, and interactions with patients, colleagues, and opinion leaders. Our observations are in line with previous work that has shown that GPs make decisions mainly based on interaction with others rather than endorsed guidelines – a process known as '*mindlines'*.(32)

There are opportunities to develop educational interventions to address some of the factors that influenced decision-making that do not rely on guideline dissemination and consider the resources and sources of information most valued by GPs. One such factor is the inaccurate belief that certain pathoanatomic diagnoses on their own should guide the decision to prescribe certain pain medicines. Pathoanatomic findings are often incidental and have been shown to be mostly unrelated to the development, or severity of symptoms.(33) Interventions could also be developed to increase GPs' awareness of their own biases and heuristics as identified by our study.

The Australian Commission on Safety and Quality in Health Care has recently launched a Clinical Care Standard for LBP to improve care for LBP. One of its core messages is around the quality use of medicines. Specifically, they recommend against anticonvulsants, benzodiazepines, and antidepressants, and that clinicians only consider opioid analgesics for carefully selected patients at the lowest dose for the shortest duration possible.(34) Strategies to implement the Clinical Care Standard may need to account for the perception amongst GPs that following guidelines may lead to patients being undertreated. Our study showed that GPs value opinion leaders as a source of information, which suggests that implementation strategies to improve the quality use of medicines for LBP could involve opinion leaders, an approach that has been shown to improve compliance with evidence-based care.(35)

A strength of our study is the use of vignettes, a widely used methodology for assessing decision-making.(36) Vignettes provided a concrete starting point so that GPs could base their decisions on a detailed patient case, rather than answering abstract questions about attitudes and perceptions. However, the decision-making processes described in our study were anchored to the information provided in the vignettes and may not necessarily be generalisable to all LBP cases or truly reflect objective prescription data.(37) For example, while our study did show that GPs would most commonly prescribe or conditionally prescribe opioids for an acute exacerbation of chronic LBP (vignette 1) or subacute sciatica (vignette 2), which agrees with Australian data on prescription medicines for LBP,[3] our finding that GPs would more commonly prescribe antidepressants for the patient with chronic LBP in vignette 3 differs from existing data. It is unknown whether this difference may be due to the characteristics of the vignette and reflective of practice for patients with the characteristics described in that vignette. Our sample was composed mostly of very experienced GPs practising in metropolitan areas. Their views may differ from those in earlier stages of their careers. For example, the youngest GP in our sample mentioned the role of University and GP training in teaching GPs about the lack of effectiveness and harms of

For peer review only - http://bmjopen.bmj.com/site/about/guidelines.xhtml

opioids for LBP. That view was not shared by any other experienced GP, which could indicate recent changes in teaching curricula. GPs practising in rural areas are more likely to prescribe opioid analgesics for LBP.(3) However, our sample lacked GPs practising in those areas, limiting our ability to contrast potential differences in drivers of decision-making between those areas.

CONCLUSIONS

We identified several aspects of decision-making that help explain the current profile of pain medicines prescribed for LBP in by GPs. Themes identified by our study could inform future implementation strategies to improve the quality use of medicines for LBP.

CONTRIBUTORS

Conceptualisation: GF, JA, CWC, BR, CN, CAS, AM, RD, CM; Methodology: GF, JA; Validation: GF, JZ; Formal analysis: GF, JZ; Investigation: GF, JZ, CJ; Resources: GF; Data curation: GF; Supervision: CM; Project administration: GF; Funding acquisition: GF, CWC, CAS, BR, AM, RD, CM; Writing – Original draft: GF; Writing – Review & editing: JZ, JA, CJ, CWC, BR, CN, CAS, AM, RD, CM

TRANSPARENCY

The lead author (GF) affirms that this manuscript is an honest, accurate, and transparent account of the study being reported; that no important aspects of the study have been omitted; and that any discrepancies from the study as planned (and, if relevant, registered) have been explained.

FUNDING

This study was partially funded by a seed grant from the Australian New Zealand Musculoskeletal Clinical Trials Network (ANZMUSC). GEF, JZ, JA, C-WC L, and CM are supported by National Health and Medical Research Council (NHMRC) fellowships (APP2009808, APP1194105, APP2017278, APP1193939, and APP11094283, respectively). CM is also supported by Centre for Research Excellence grants (APP1134856, APP1171459, APP2006545).

COMPETING INTERESTS DECLARATION

The Sydney Pharmacy School receives funding from GlaxoSmithKline for a postgraduate scholarship supervised by AM. CM has received research grants from various government and not for profit agencies. Flexeze provided heat wraps at no cost for the SHaPED trial for which CM, BR, and CN are investigators. Other authors have no interests to declare.

ETHICAL APPROVAL

The University of Sydney Human Research Ethics Committee approved this research (approval number: 2022/170).

DATA AVAILABILITY

Data are available upon request.

DATA SOURCE

Data collected by the investigators.

LICENSE STATEMENT

For peer review only

The corresponding author (GF) has the right to grant and does grant on behalf of all authors of the Work (as defined in the below author licence), an exclusive licence and/or a non-exclusive licence for contributions from authors who are: i) UK Crown employees; ii) where BMJ has agreed a CC-BY licence shall apply, and/or iii) in accordance with the terms applicable for US Federal Government officers or employees acting as part of their official duties; on a worldwide, perpetual, irrevocable, royalty-free basis to BMJ Publishing Group Ltd ("BMJ") its licensees and where the relevant Journal is co-owned by BMJ to the co-owners of the Journal, to publish the Work in BMJ Quality & Safety and any other BMJ products and to exploit all rights, as set out in our licence.

The corresponding author (GF) accepts and understands that any supply made under these terms is made by BMJ to the Submitting Author unless you are acting as an employee on behalf of your employer or a postgraduate student of an affiliated institution which is paying any applicable article publishing charge ("APC") for Open Access articles. Where the Submitting Author wishes to make the Work available on an Open Access basis (and intends to pay the relevant APC), the terms of reuse of such Open Access shall be governed by a Creative Commons licence – details of these licences and which Creative Commons licence will apply to this Work are set out in our licence referred to above.

REFERENCES

1. Australian Institute of Health and Welfare. Back problems. Canberra: AIHW; 2020.
2. Williams CM, Maher CG, Hancock MJ, McAuley JH, McLachlan AJ, Britt H, et al. Low back pain and best practice care: A survey of general practice physicians. Arch Intern Med. 2010;170(3):271-7.
3. Mathieson S, Valenti L, Maher CG, Britt H, Li Q, McLachlan AJ, et al. Worsening trends in analgesics recommended for spinal pain in primary care. Eur Spine J. 2018;27(5):1136-45.
4. NICE. Low back pain and sciatica in over 16s: assessment and management 2020 [Available from: https://www.nice.org.uk/guidance/ng59.
5. Enke O, New HA, New CH, Mathieson S, McLachlan AJ, Latimer J, et al. Anticonvulsants in the treatment of low back pain and lumbar radicular pain: a systematic review and meta-analysis. Cmaj. 2018;190(26):E786-e93.
6. Mathieson S, Maher CG, McLachlan AJ, Latimer J, Koes BW, Hancock MJ, et al. Trial of Pregabalin for Acute and Chronic Sciatica. New England Journal of Medicine. 2017;376(12):1111-20.
7. Ivanova JI, Birnbaum HG, Schiller M, Kantor E, Johnstone BM, Swindle RW. Real-world practice patterns, health-care utilization, and costs in patients with low back pain: the long road to guideline-concordant care. Spine J. 2011;11(7):622-32.
8. Gouveia N, Rodrigues A, Ramiro S, Eusébio M, Machado PM, Canhão H, et al. The Use of Analgesic and Other Pain-Relief Drugs to Manage Chronic Low Back Pain: Results from a National Survey. Pain Practice. 2017;17(3):353-65.
9. Britt H MG, Bayram C, Henderson J, Valenti L, Harrison C, Pan Y, Charles J,, Pollack AJ CT, Gordon J, Wong C. A decade of Australian general practice activity 2006–07 to 2015–16. Sydney: Sydney University Press, 2016; 2016.
10. Bishop FL, Dima AL, Ngui J, Little P, Moss-Morris R, Foster NE, et al. "Lovely Pie in the Sky Plans": A Qualitative Study of Clinicians' Perspectives on Guidelines for Managing Low Back Pain in Primary Care in England. Spine (Phila Pa 1976). 2015;40(23):1842-50.
11. Tong A, Sainsbury P, Craig J. Consolidated criteria for reporting qualitative research (COREQ): a 32-item checklist for interviews and focus groups. Int J Qual Health Care. 2007;19(6):349-57.
12. Jensen RK, Kongsted A, Kjaer P, Koes B. Diagnosis and treatment of sciatica. BMJ. 2019;367:l6273.
13. Lundgren-Laine H, Salantera S. Think-aloud technique and protocol analysis in clinical decision-making research. Qual Health Res. 2010;20(4):565-75.
14. O'Keeffe M, Michaleff ZA, Harris IA, Buchbinder R, Ferreira GE, Zadro JR, et al. Public and patient perceptions of diagnostic labels for non-specific low back pain: a content analysis. European Spine Journal. 2022;31(12):3627-39.

For peer review only - http://bmjopen.bmj.com/site/about/guidelines.xhtml

15. Ferreira GE, Zadro J, O'Keeffe M, Chen M, Gao Y, Hu Z, et al. A content analysis of online information about the benefits and harms of spine surgery. Braz J Phys Ther. 2022;26(2):100398.
16. Gale NK, Heath G, Cameron E, Rashid S, Redwood S. Using the framework method for the analysis of qualitative data in multi-disciplinary health research. BMC Medical Research Methodology. 2013;13(1):117.
17. Ayre J, McCaffery KJ. Research Note: Thematic analysis in qualitative research. J Physiother. 2022;68(1):76-9.
18. Ferreira GE, McLachlan AJ, Lin CC, Zadro JR, Abdel-Shaheed C, O'Keeffe M, et al. Efficacy and safety of antidepressants for the treatment of back pain and osteoarthritis: systematic review and meta-analysis. BMJ. 2021;372:m4825.
19. Ferreira GE, Machado GC, Abdel Shaheed C, Lin CC, Needs C, Edwards J, et al. Management of low back pain in Australian emergency departments. BMJ Qual Saf. 2019;28(10):826-34.
20. Ferreira GE, Abdel-Shaheed C, Underwood M, Finnerup NB, Day RO, McLachlan A, et al. Efficacy, safety, and tolerability of antidepressants for pain in adults: overview of systematic reviews. BMJ. 2023;380:e072415.
21. Therapeutic Guidelines. Melbourne: Therapeutic Guidelines Limited; [Available from: https://tg.org.au.
22. Interian A, Martinez IE, Guarnaccia PJ, Vega WA, Escobar JI. A qualitative analysis of the perception of stigma among Latinos receiving antidepressants. Psychiatr Serv. 2007;58(12):1591-4.
23. Mathieson S, Lin CC, Underwood M, Eldabe S. Pregabalin and gabapentin for pain. BMJ. 2020;369:m1315.
24. Mathieson S, O'Keeffe M, Traeger AC, Ferreira GE, Abdel Shaheed C. Content and sentiment analysis of gabapentinoid-related tweets: An infodemiology study. Drug and Alcohol Review. 2022;n/a(n/a).
25. Therapeutic Goods Administration. Pregabalin and gabapentin. Safety advisory - enhanced warnings relating to abuse and dependence 2021 [Available from: https://www.tga.gov.au/news/safety-alerts/pregabalin-and-gabapentin.
26. Tamblyn R, Berkson L, Dauphinee WD, Gayton D, Grad R, Huang A, et al. Unnecessary prescribing of NSAIDs and the management of NSAID-related gastropathy in medical practice. Ann Intern Med. 1997;127(6):429-38.
27. Campbell SM, Hann M, Hacker J, Burns C, Oliver D, Thapar A, et al. Identifying predictors of high quality care in English general practice: observational study. BMJ. 2001;323(7316):784.
28. Tsiga E, Panagopoulou E, Sevdalis N, Montgomery A, Benos A. The influence of time pressure on adherence to guidelines in primary care: an experimental study. BMJ Open. 2013;3(4):e002700.
29. Privett N, Guerrier S. Estimation of the Time Needed to Deliver the 2020 USPSTF Preventive Care Recommendations in Primary Care. Am J Public Health. 2021;111(1):145-9.
30. Porter J, Boyd C, Skandari MR, Laiteerapong N. Revisiting the Time Needed to Provide Adult Primary Care. J Gen Intern Med. 2023;38(1):147-55.
31. Bardin LD, King P, Maher CG. Diagnostic triage for low back pain: a practical approach for primary care. Med J Aust. 2017;206(6):268-73.
32. Gabbay J, le May A. Evidence based guidelines or collectively constructed "mindlines?" Ethnographic study of knowledge management in primary care. Bmj. 2004;329(7473):1013.
33. Kasch R, Truthmann J, Hancock MJ, Maher CG, Otto M, Nell C, et al. Association of Lumbar MRI Findings with Current and Future Back Pain in a Population-based Cohort Study. Spine. 2022;47(3).
34. Australian Commission on Safety and Quality in Health Care.Low Back Pain Clinical Care Standard. Sydney: ACSQHC; 2022.
35. Flodgren G, O'Brien MA, Parmelli E, Grimshaw JM. Local opinion leaders: effects on professional practice and healthcare outcomes. Cochrane Database Syst Rev. 2019;6(6):Cd000125.
36. Evans SC, Roberts MC, Keeley JW, Blossom JB, Amaro CM, Garcia AM, et al. Vignette methodologies for studying clinicians' decision-making: Validity, utility, and application in ICD-11 field studies. Int J Clin Health Psychol. 2015;15(2):160-70.
37. Alexander CS, Becker HJ. The Use of Vignettes in Survey Research. Public Opinion Quarterly. 1978;42(1):93-104.

For peer review only - http://bmjopen.bmj.com/site/about/guidelines.xhtml

Table 1. Features of each clinical vignette.

	Vignette 1	Vignette 2	Vignette 3
**Patient characteristics**			
Female	X		X
Male		X	
Middle-aged	X	X	X
Physical activity			
No physical activity	X		
Insufficient physical activity			X
Physically active		X	
**Social aspects**			
Children			X
Primary carer for older person	X		
**Type of low back pain**			
Atraumatic back pain	X	X	X
Back pain initiated after a well-defined event		X	
Back pain with a clear radicular component		X	
Back pain with referred leg pain			X
**Pain characteristics**			
Night pain	X	X	
Sleep disturbance	X		
Back pain exacerbated by movements/postures		X	X
Acute-on-chronic pain	X		
Sub-acute (pain between 6 and 12 weeks)		X	
Chronic (pain > 12 weeks)			X
Movement restriction	X	X	
Limitations to work		X	
**Comorbidities**			
Obesity	X		
Gastro-oesophageal reflux disease	X		
High cholesterol	X		
Other musculoskeletal conditions	X		
Diabetes			X
Depression (mild)			X
**Healthcare use**			
General practitioner for low back pain		X	
Physiotherapy for low back pain			X
Acupuncture for low back pain			X
Psychologist for mild depression			X
Previous imaging	X		
Previous/current use of pain medicines	X	X	X
Paracetamol, NSAIDs	X		X
Opioids		X	

For peer review only - http://bmjopen.bmj.com/site/about/guidelines.xhtml

Table 2. Characteristics of participants (n = 25)

Age, mean (SD)	53.4 (9.1)
Sex, n (%)	
Female	9 (36%)
Male	16 (64%)
State/territory	
New South Wales	9 (36%)
Queensland	6 (24%)
Victoria	5 (20%)
South Australia	2 (8%)
Western Australia	2 (8%)
Tasmania	1 (4%)
Remoteness[1]	
Metropolitan	22 (88%)
Rural/remote	3 (12%)
Number of patients with low back pain seen per year, n (%)	
5-25	2 (8%)
26-50	4 (16%)
51-100	3 (12%)
>100	16 (64%)
Years of experience, n (SD) [min – max]	24.6 (9.3) [5 – 40]
Weekly clinical workload (hours), mean (SD) [min – max]	38 (7.7) [14 – 50]

[1]Defined per the Australian Statistical Geography Standard; GPs working in metropolitan areas are those whose practice is located in a Greater Capital City Statistical Area.

For peer review only

Table 3. Frequency of pain medicines that GPs would prescribe, would conditionally prescribe, were against prescribing, or that were not mentioned for each vignette.

	Paracetamol	NSAIDs	Opioids	Antidepressants	Anticonvulsants	Oral steroids	Benzodiazepines
**Vignette 1 (n = 25)**							
Would prescribe	13	17	8	6	1	0	0
Would conditionally prescribe	0	3	14	3	0	1	3
Against prescribing	0	2	2	6	9	1	1
Not mentioned	12	3	1	10	15	23	21
**Vignette 2 (n = 25)**							
Would prescribe	8	16	7	6	11	3	0
Would conditionally prescribe	0	0	12	7	5	2	0
Against prescribing	0	2	6	3	5	3	1
Not mentioned	17	7	0	9	4	17	24
**Vignette 3 (n = 24)***							
Would prescribe	8	8	8	10	5	0	1
Would conditionally prescribe	1	1	3	5	2	0	0
Against prescribing	0	2	4	0	1	2	0
Not mentioned	15	13	9	9	16	22	23

GP, General practitioner; NSAIDs, non-steroidal anti-inflammatories
*Data from 24 GPs shown. The interview with one GP discussing vignette 3 was not recorded due to technical issues.

For peer review only - http://bmjopen.bmj.com/site/about/guidelines.xhtml

Table 4. Themes, subthemes, and supporting quotes.

Themes	Subthemes	Supporting quotes
Prioritising personal information sources	Previous experience	• As an experienced GP of just over 20 years, you have scripts in your mind that bring you to a sort of automatic pilot approach. (GP #20, male, 21 years of experience)<br>• I'd probably just give her the options of weather she wants some anti-inflammatory, something with codeine, and antidepressant. (GP #3, female, 23 years of experience)<br>• I've had radicular pain myself, and the only thing that fixed me was Lyrica. I actually had little faith in Lyrica before I used it, but nothing else touched my pain. A lot of my approach to radicular pain is based on my own experience. (GP #1, female, 30 years of experience)
	Guidelines are unhelpful	• Sometimes I find guidelines are not actually practical. If they say 'oh tell the patient they need to keep fit and tell them that pain is not a bad sign and that they should keep active and a little bit of pain is ok', it can be hard to convince patients of that. (GP #1, female, 30 years of experience)
	Clinical training	• Probably what I've heard at specialist meetings when the specialists have spoken. I went to one last night and the neurosurgeon was basically saying you try and avoid going on the path of long-acting opioids, and you can use a quick acting opioid. (GP #6, female, 35 years of experience).<br>• Part of it is what we what we've learned through GP registrar training and medical school, where there's a strong emphasis on not using opioid painkillers in for the management of back pain (GP #17, male, 5 years of experience)
Strong reliance on a biomedical model to guide prescription	Pathoanatomical diagnosis	• When we do the imaging, it's going to support our treatment more. If you find one of two nerves there affecting him, we can start him on pregabalin 25 mg (GP #23, female, 28 years of experience)<br>• If we found multiple levels of a minor disc bulge or something, I would be happier to give her something stronger, but if the MRI just showed that she had a disc bulge at one level and everything else looks fine, there's no nerve impingement, I don't think that would warrant it. I'd be happy to try amitriptyline than other medications as a first line. (GP #9, female, 25 years of experience).
	Sleep patterns	• I'd be happy to give her some panadeine [NB: codeine + paracetamol] straight away because it's keeping her awake at night, and that's always a bit of a problem. She wakes up because of the pain… I mean you don't want people waking up with pain, so give her a bit of mild pain relief. (GP #22, male, 39 years of experience)
	Pain severity	• No because she might still respond to what I've decided to use, it might just be temporary that she's eight or nine [NB: pain out of 10] (GP #4, female, 30 years of experience)
	Comorbidities	• There are some other antidepressants like Cymbalta which can act as a neuropathic agent as well as an antidepressant. So there are antidepressants which we can use which help patients manage their depression as well as well as their pain. (GP #19, male, 30 years of experience)
Social factors as root causes	pain medicines do not address the root cause of the problem	• We don't want her having a fall with her mum and we don't want her experiencing side effects that may affect her ability to care for her mother. (GP #7, male, 18 years of experience)
	Psychosocial-based prescribing	• Since she's getting relief with paracetamol and ibuprofen, I think the next step is to find if there are any associated issues in terms of looking after her mum. Can we get some home help… refer mum to aged care services? I mean she might be able to get NDIS help [NB: government support] as well (GP #11, male, 20 years of experience)

For peer review only - http://bmjopen.bmj.com/site/about/guidelines.xhtml

Judicious use of pain medicines	Keep it simple or make it complex?	• They get flare ups here and there and I think you don't jump up and down as long as you know exactly the origin of the pain and you know you excluded that there is more to it than just what's presenting at this stage, and I think let's just keep it simple. And you can always go back and increase the medication or order further investigations if things don't subside, but I don't hit them harder on the 1st presentation. (GP #15, male, 30 years of experience)<br><br>• I tend to like to use a smaller dose of a lot of things rather than a bigger dose of one or two things. So I'd write a little plan and it would have a sliding scale so it would start off with the Panadol three times a day plus the Nurofen. That's the foundations of the house, and then I would add into that tramadol sustained release 150 to 100 twice a day, and then I would usually add in amitriptyline 10 to 20 milligrams before bed. (GP #25, female, 30 years of experience)
Concerns about prescription	drug seeking behaviour and risk of long-term misuse	• It's a chronic, insidious, ongoing bit of depression. And it just typically… It's just super clean. She gets started on endone and then get stuck on endone and that becomes their whole life. And they just slowly over a course of years dose-escalate. And they feel better. Because of the euphoria associated with the oxycodone. Terrible. (GP #16, male, 34 years of clinical experience)
	Side effects of prescription pain medicines	• Opioids induce hyperalgesia, which comes with a lot of opioids such as codeine, which is a poor analgesic. I don't use that either. I basically use 3 analgesics in my practice: tramadol, buprenorphine, and tapentadol. (GP #19, Male, 30 years of experience)
	Stigma around prescription	• It could be done, but it would involve a very lengthy consultation and we as GPs don't have half an hour to 45 minutes to discuss something a little bit more wayward. (GP #7, male, 18 years of experience)
	Pressure from regulators	• The guidelines at the moment for non-opioid medication are clear as far as we can all see. I don't know how much that'll go the wrong way and we'll end up not treating anybody pains eventually. We get sued for not treating anybody pain and the pain and suffering and next thing you know the guidelines will change, you know? But you can't win, can you? (GP #14, male, 40 years of experience)<br><br>• Department of Health want us not to give pain medicine at all for low back pain but sometimes it's not realistic. Sometimes we need to help the patient even though I mean, potentially they can be additive of course, but, realistically, the patient is in so much pain, they can't move. This is cruel. Not giving opioid medicines, at least for a few days. I'm not the type of person that gives everybody strong pain medicines, but I think there is a role in some patients. (GP #18, male, 27 years of experience)

For peer review only

General practitioners' decision-making process to prescribe pain medicines for low back pain: a qualitative study

Contents

Supplementary file 1. Vignettes 2

Supplementary file 2. Interview guide. 5

For peer review only

Supplementary file 1. Vignettes

Prompt: I will now present to you three clinical vignettes describing different low back pain presentations. After each vignette I am going to ask you a series of questions. Please draw on your prior experience to answer the questions about how you would manage these back pain patients. There are no right or wrong answers. We are interested in honest opinions, not idealised ones (for example, what you should do). Remember that your data will be anonymised.

VIGNETTE 1

- Patient info: Female patient, 57 years old, BMI 33 (height: 1.60m; weight: 85kg)
- Complaint: atraumatic onset low back pain that started 2 weeks ago. Average pain 5/10; worse in the evening/at night with pain 7/10.
- History:
    - Low back pain for 20+ years (ongoing mild pain 2/10) with occasional moderate flares (pain up to 5/10) that typically resolve in a few days. Previous MRIs (last one 3 years ago) showed the following: At L5/S1 there is vertebral endplate bone marrow oedema. The distinction between the nucleus and annulus is lost with a with loss of disc height.
    - She has sought care for back pain a few times before, but typically self-manages the pain with simple painkillers (Paracetamol and occasionally ibuprofen as needed). She is seeking care for this episode because it's taking longer to resolve, and the pain is more intense in the evening/at night
    - Patient is having difficulty sleeping – she wakes up because of pain when turning in bed.
    - Patient is sedentary and is the primary carer of her 88-year-old mother who is bed-bound due to a stroke 12 months ago.
    - Comorbidities: Obesity, GORD, high cholesterol (daily atorvastatin), medial compartment R knee osteoarthritis (also treats with simple analgesics as needed).
- Physical examination:
    - No evidence of radicular symptoms: straight leg raise, sensation, reflexes, and lower limb strength are normal. No signs of infection or midline tenderness in the lumbar spine. Tenderness over the paraspinal muscles is present.
    - Movements in every direction are slightly more painful (6/10)

For peer review only

VIGNETTE 2

- Patient info: 45-year-old male, BMI 26 (height: 1.83m; weight: 87kg)

- Complaint: Acute onset low back pain with shooting stabbing pain radiating down the back of the right leg to the foot and associated with pins and needles in the same area. This commenced 8 weeks ago after trying to lift a heavy box while visiting family in rural NSW. Main complaint is leg pain (5/10), but he also has pain in the lower back (3/10). Sitting for >1h aggravates leg pain (7/10), sit-to-standing and vice-versa are quite painful (7/10).

- History:

  - No history of low back pain or any other musculoskeletal condition prior to this episode. Patient is physically active, swims and runs twice a week. Sitting for more than 1 hour aggravates pain and is severely limiting his ability to work. He has not taken any time off work.
  - Patient went to the local general practitioner the day after pain onset who prescribed codeine 30mg QID for 1 week. No improvement was noted. No significant side effects were noted either besides constipation.
  - Patient has not reported any significant sleep disturbance

- Physical examination:
  - Pain follows a dermatomal distribution (S1), with altered sensation to light touch. Muscle strength and reflexes are preserved.
  - No signs of infection or midline tenderness in the lumbar spine.
  - Straight leg raise positive at ~30deg; crossed straight leg raise test is negative
  - Flexion and extension movements are limited and increase leg pain (7/10). Rotations and inclinations do not change symptoms.
  - Cauda equina syndrome and Other red flags were excluded

For peer review only

VIGNETTE 3

- Patient info: Female patient, 52 years old, BMI 30 (height: 1.63m; weight: 79kg)

- Complaint: Insidious onset low back pain radiating to the L thigh that started 6 months ago. Patient cannot recall an event that triggered the pain. Pain is bilateral in the lower back and radiates to the L posterior thigh with the same average intensity (5/10). Standing up/walking for too long (>1h) increases pain (6-7/10). Sitting/lying down reduces pain. She does not have trouble sleeping.

- History:

  - No history of low back pain or any other musculoskeletal condition prior to this episode. Patient is married with 3 children (youngest is 20 years-old),
  - Patient has recently been diagnosed with type II diabetes (she takes metformin daily and has been going to the gym 3 times/week for about 45 minutes where she does low-moderate intensity resistance training since the diagnosis – 2 years ago. Her sugar levels are currently controlled – around 5mmol/L before meals. Kidney function is normal). She was diagnosed with mild depression two years ago and has been supported by a psychologist with training in cognitive behavioural therapy. No other pharmacological treatments are used for depression.
  - She uses over-the-counter analgesics (Paracetamol and Ibuprofen) regularly on an as-needed basis to manage the pain. She has seen a physiotherapist (core strengthening exercises) and an acupuncturist but has not responded to either treatment.

- Physical examination:
  - No abnormalities on neurological exam (sensation, strength, and reflexes are all normal).
  - No signs of infection or midline tenderness in the lumbar spine.
  - Straight leg raise and crossed Straight leg raise negative.
  - Lumbar spine movements are limited mostly by lack of flexibility. End-range lumbar extension increases pain momentarily (7/10), which reduces as soon as patient returns to neutral position.

For peer review only

Supplementary file 2. Interview guide.

Questions - Choosing between different types of pharmacological treatments

Prompt: Answer the following questions based on the patient vignette you just read. Using the information from the vignette, I would like you to think aloud your clinical reasoning.

- Would you typically prescribe a pain medicine for the patient described in the vignette?

- Could you elaborate on which pain medicines you would prescribe and why you would prescribe them *[remind the participant of the medicines they mentioned]*?

Prompt participant to talk about their rationale to prescribe/recommend certain medicines; what kind of thought process they follow, sources of information that they use to make their decision:

- *Clinical presentation (e.g. affected sleep in vignette 1; neuropathic pain features in vignette 2; depression in vignette 3, etc.)*
- *Severity of symptoms*
- *Known efficacy from trials and systematic reviews*
- *Guideline recommendation*
- *Safety/side effects*
- *Previous use by patient*
- *Previous success with other patients*
- *Demographic factors*
- *Mechanisms of action of the drug*
- *Intuition*

Questions – influence of factors not mentioned by the participant

- How do aspects like (eg age, gender, comorbidities, description of pain, etc.) factor in your decision to prescribe 'ABC' *[the pain medicines mentioned by the participant]*?

Questions - Alternatives

- What about pain medicines 'XYZ'? Would you prescribe any of these for the vignette? *[mention pain medicines that the participant did not mention eg strong opioids [oxycodone endone], weak opioids [codeine], gabapentin, pregabalin, amitriptyline, oral steroids, etc.)]*

- Are there any circumstances where you would prescribe XYZ instead or/in addition to ABC for the patient in this vignette?

Questions – external factors influencing decision-making

- Would your decision-making change if you were pressed for time? *[eg do they report using any heuristic/rules of thumb when they are time-limited?]*
- Would your decision-making change depending on patients' opinions on the treatments you typically prescribe?

For peer review only

COREQ (COnsolidated criteria for REporting Qualitative research) Checklist

A checklist of items that should be included in reports of qualitative research. You must report the page number in your manuscript where you consider each of the items listed in this checklist. If you have not included this information, either revise your manuscript accordingly before submitting or note N/A.

Topic	Item No.	Guide Questions/Description	Reported on Page No.
**Domain 1: Research team and reflexivity**			
Personal characteristics			
Interviewer/facilitator	1	Which author/s conducted the interview or focus group?	
Credentials	2	What were the researcher's credentials? E.g. PhD, MD	
Occupation	3	What was their occupation at the time of the study?	
Gender	4	Was the researcher male or female?	
Experience and training	5	What experience or training did the researcher have?	
Relationship with participants			
Relationship established	6	Was a relationship established prior to study commencement?	
Participant knowledge of the interviewer	7	What did the participants know about the researcher? e.g. personal goals, reasons for doing the research	
Interviewer characteristics	8	What characteristics were reported about the inter viewer/facilitator? e.g. Bias, assumptions, reasons and interests in the research topic	
**Domain 2: Study design**			
Theoretical framework			
Methodological orientation and Theory	9	What methodological orientation was stated to underpin the study? e.g. grounded theory, discourse analysis, ethnography, phenomenology, content analysis	
Participant selection			
Sampling	10	How were participants selected? e.g. purposive, convenience, consecutive, snowball	
Method of approach	11	How were participants approached? e.g. face-to-face, telephone, mail, email	
Sample size	12	How many participants were in the study?	
Non-participation	13	How many people refused to participate or dropped out? Reasons?	
Setting			
Setting of data collection	14	Where was the data collected? e.g. home, clinic, workplace	
Presence of non-participants	15	Was anyone else present besides the participants and researchers?	
Description of sample	16	What are the important characteristics of the sample? e.g. demographic data, date	
Data collection			
Interview guide	17	Were questions, prompts, guides provided by the authors? Was it pilot tested?	
Repeat interviews	18	Were repeat inter views carried out? If yes, how many?	
Audio/visual recording	19	Did the research use audio or visual recording to collect the data?	
Field notes	20	Were field notes made during and/or after the inter view or focus group?	
Duration	21	What was the duration of the inter views or focus group?	
Data saturation	22	Was data saturation discussed?	
Transcripts returned	23	Were transcripts returned to participants for comment and/or	

For peer review only

Topic	Item No.	Guide Questions/Description	Reported on Page No.
		correction?	
**Domain 3: analysis and findings**			
Data analysis			
Number of data coders	24	How many data coders coded the data?	
Description of the coding tree	25	Did authors provide a description of the coding tree?	
Derivation of themes	26	Were themes identified in advance or derived from the data?	
Software	27	What software, if applicable, was used to manage the data?	
Participant checking	28	Did participants provide feedback on the findings?	
Reporting			
Quotations presented	29	Were participant quotations presented to illustrate the themes/findings? Was each quotation identified? e.g. participant number	
Data and findings consistent	30	Was there consistency between the data presented and the findings?	
Clarity of major themes	31	Were major themes clearly presented in the findings?	
Clarity of minor themes	32	Is there a description of diverse cases or discussion of minor themes?	

Developed from: Tong A, Sainsbury P, Craig J. Consolidated criteria for reporting qualitative research (COREQ): a 32-item checklist for interviews and focus groups. *International Journal for Quality in Health Care*. 2007. Volume 19, Number 6: pp. 349 – 357

Once you have completed this checklist, please save a copy and upload it as part of your submission. DO NOT include this checklist as part of the main manuscript document. It must be uploaded as a separate file.

For peer review only

BMJ Open

General practitioners' decision-making process to prescribe pain medicines for low back pain: a qualitative study

Journal:	*BMJ Open*
Manuscript ID	bmjopen-2023-074380.R2
Article Type:	Original research
Date Submitted by the Author:	11-Sep-2023
Complete List of Authors:	Ferreira, Giovanni; University of Sydney School of Public Health, Institute for Musculoskeletal Health<br>Zadro, Joshua; University of Sydney, Institute for Musculoskeletal Health, School of Public Health<br>Jones, Caitlin; The University of Sydney Institute for Musculoskeletal Health, School of Public Health<br>Ayre, Julie; The University of Sydney, Sydney Health Literacy Lab<br>Lin, Christine; University of Sydney School of Public Health, Institute for Musculoskeletal Health<br>Richards, Bethan; The University of Sydney Institute for Musculoskeletal Health<br>Needs, Christopher; Institute of Rheumatology & Orthopaedics, Rheumatology Department<br>Abdel Shaheed, Christina; University of Sydney School of Public Health, Institute for Musculoskeletal Health<br>McLachlan, Andrew; University of Sydney, Faculty of Pharmacy<br>Day, Richard; St Vincent's Hospital, Department of Clinical Pharmacology & Toxicology; University of New South Wales, St Vincent's Clinical School, Faculty of Medicine<br>Maher, Christopher; University of Sydney, Institute for Musculoskeletal Health, Sydney School of Public Health, Faculty of Medicine and Health
**Primary Subject Heading**:	General practice / Family practice
Secondary Subject Heading:	Qualitative research
Keywords:	Decision Making, Back pain < ORTHOPAEDIC & TRAUMA SURGERY, QUALITATIVE RESEARCH

SCHOLARONE™
Manuscripts

I, the Submitting Author has the right to grant and does grant on behalf of all authors of the Work (as defined in the below author licence), an exclusive licence and/or a non-exclusive licence for contributions from authors who are: i) UK Crown employees; ii) where BMJ has agreed a CC-BY licence shall apply, and/or iii) in accordance with the terms applicable for US Federal Government officers or employees acting as part of their official duties; on a worldwide, perpetual, irrevocable, royalty-free basis to BMJ Publishing Group Ltd ("BMJ") its licensees and where the relevant Journal is co-owned by BMJ to the co-owners of the Journal, to publish the Work in this journal and any other BMJ products and to exploit all rights, as set out in our licence.

The Submitting Author accepts and understands that any supply made under these terms is made by BMJ to the Submitting Author unless you are acting as an employee on behalf of your employer or a postgraduate student of an affiliated institution which is paying any applicable article publishing charge ("APC") for Open Access articles. Where the Submitting Author wishes to make the Work available on an Open Access basis (and intends to pay the relevant APC), the terms of reuse of such Open Access shall be governed by a Creative Commons licence – details of these licences and which Creative Commons licence will apply to this Work are set out in our licence referred to above.

Other than as permitted in any relevant BMJ Author's Self Archiving Policies, I confirm this Work has not been accepted for publication elsewhere, is not being considered for publication elsewhere and does not duplicate material already published. I confirm all authors consent to publication of this Work and authorise the granting of this licence.

For peer review only

General practitioners' decision-making process to prescribe pain medicines for low back pain: a qualitative study

Giovanni E Ferreira[1,2], Joshua R Zadro[1,2], Caitlin Jones[1,2], Julie Ayre[1,2], Chung-Wei Christine Lin[1,2], Bethan Richards[2,4], Christopher Needs[4], Christina Abdel Shaheed[1,2], Andrew McLachlan[5], Richard Day[6], Chris G Maher[1,2]

1. Sydney Musculoskeletal Health, Faculty of Medicine and Health, The University of Sydney
2. Institute for Musculoskeletal Health, The University of Sydney and Sydney Local Health District
3. Sydney Health Literacy Lab, School of Public Health, Faculty of Medicine and Health, The University of Sydney, Sydney, Australia
4. Department of Rheumatology, Royal Prince Alfred Hospital, Sydney, Australia
5. Sydney Pharmacy School, Faculty of Medicine and Health, The University of Sydney, Sydney, Australia
6. St Vincent's Clinical School, Department of Clinical Pharmacology & Toxicology, University of New South Wales, Sydney, Australia

Abstract word count: 291
Manuscript word count: 3186

Address all correspondence to:

Giovanni E. Ferreira
School of Public Health, Faculty of Medicine and Health, The University of Sydney, Sydney, New South Wales, Australia
PO Box M179, Missenden Road, Camperdown | NSW | 2050, Australia
E-mail: giovanni.ferreira@sydney.edu.au

For peer review only

For peer review only - http://bmjopen.bmj.com/site/about/guidelines.xhtml

ABSTRACT

Background: Pain medicines are widely prescribed by general practitioners (GPs) when managing people with low back pain (LBP), but little is known about what drives decisions to prescribe these medicines.

Objectives: The aim of this study was to investigate what influences GPs' decision to prescribe pain medicines for LBP.

Design: Qualitative study with in-depth interviews.

Setting: Australian primary care.

Participants: We interviewed 25 GPs practising in Australia experienced in managing LBP (mean [SD] age 53.4 [9.1] years, mean [SD] years of experience: 24.6 [9.3], 36% female). GPs were provided three vignettes describing common LBP presentations (acute exacerbation of chronic LBP, subacute sciatica, and chronic LBP) and were asked to think aloud how they would manage the cases described in the vignettes.

Data analysis: We summarised GP's choices of pain medicines for each vignette using content analysis and used framework analysis to investigate factors that affected GP's decision-making.

Results: GPs more commonly prescribed opioid analgesics. Anticonvulsants and antidepressants were also commonly prescribed depending on the presentation described in the vignette. GP participants made decisions about what pain medicines to prescribe for LBP largely based on previous experiences, including their own personal experiences of LBP, rather than guidelines. The choice of pain medicine was influenced by a range of clinical factors, more commonly the patient's pathoanatomical diagnosis. While many adhered to principles of judicious use of pain medicines, polypharmacy scenarios were also common. Concerns about drug seeking behaviour, adverse effects, stigma around opioid analgesics, and pressure from regulators also shaped their decision-making process.

Conclusions: We identified several aspects of decision-making that help explain the current profile of pain medicines prescribed for LBP by GPs. Themes identified by our study could inform future implementation strategies to improve the quality use of medicines for LBP.

Keywords: decision-making, opioids, low back pain, prescription, sciatica

For peer review only - http://bmjopen.bmj.com/site/about/guidelines.xhtml

Strengths and limitations of the study

- We used clinical vignettes resembling common low back pain cases to provide clinicians with a concrete starting point where they could base their decisions on
- Voices and perspectives of younger general practitioners or general practitioners working in rural and remote areas may have been underrepresented.

For peer review only

INTRODUCTION

Low back pain (LBP) affects over 4 million Australians every year and pain medicines are widely used in its management.(1) Australian data shows that 2 in every 3 patients who seek primary care are prescribed or recommended at least one pain medicine.(2) Examples include over-the-counter analgesics (e.g. paracetamol, and non-steroidal anti-inflammatories (NSAIDs)) and prescription medicines such as opioid analgesics, gabapentinoids, antidepressants, and muscle relaxants.(3)

There is a mismatch between the pharmacological therapies recommended in clinical practice guidelines for LBP and what is prescribed or recommended to patients in primary care. For example, opioid analgesics are one of the most commonly prescribed medicines for people presenting with LBP across various settings and their use has increased 39% in the last decade.(3) This is despite guidelines discouraging their use for LBP when pain is not severe or as first-line care before trialling other treatments.(3, 4) Similarly, gabapentinoids are ineffective and potentially harmful for the management of LBP; however their use is increasing.(4-6) On the other hand, the use of pharmacological therapies that are recommended as first line care such as NSAIDs has reduced 33% from 2004 to 2014.(3) In the United States, antidepressants are prescribed to about a quarter of patients with LBP.(7) In Portugal, 1 in 7 people with chronic LBP report using antidepressants and benzodiazepines for pain management.(8) However, LBP guidelines recommend against both medicines.(4)

In Australia, LBP is the sixth most commonly managed condition overall and the most common musculoskeletal condition managed by general practitioners (GPs).(9) A UK-based study provided some insight into aspects that shape the decision-making process of health professionals, including GPs. That study described how clinicians select treatments based upon knowing the individual patient, the patient's LBP, and common treatment options.(10) However, this previous study did not explore the decision-making processes that influence a GP's decision to prescribe pain medicines for LBP in-depth, particularly in relation to the processes that underpin the choice for different pain medicines. Therefore, the aim of this qualitative study was to investigate what influences GPs' decision-making process for prescribing pain medicines for people with LBP.

METHODS

Design

This qualitative study was approved by XXXXX Human Research Ethics Committee (approval number: 2022/170) and is reported per the COREQ statement.(11)

Participant selection and recruitment

We recruited 25 GPs registered to practise in Australia who reported having provided care for at least five patients with LBP with or without sciatica in the last 12 months. Four or less patients is what our team thought would be representative of little to no experience in managing LBP. We defined sciatica as a back problem with radiating pain below the knee with or without neurological symptoms (e.g. reduced muscle strength, sensation, or reflexes).(12)

For peer review only - http://bmjopen.bmj.com/site/about/guidelines.xhtml

GPs were recruited via a market research company (TKW Research Group, Australia) with a large database of healthcare professionals across Australia and New Zealand. Health professionals opt into this database and therefore have already indicated a willingness to participate in research. None of the participants had a prior relationship with the study investigators. The company sent out emails to GPs in their database and scheduled interviews with those who expressed interest in the study.

Interviews and data collection

We conducted in-depth, one-on-one semi-structured interviews. Interviews were conducted by videoconference between May and June 2022, audio recorded, and transcribed verbatim. A researcher with a background in occupational therapy and experience in qualitative interviews and pain management research conducted the interviews.

An interview guide and three clinical vignettes to guide the interviews were developed by the lead author, a physiotherapist with experience in LBP research and pain management, and two experienced rheumatologists who provide care for patients with LBP (supplementary file 1). The vignettes were designed to represent three common LBP presentations: a patient with an acute exacerbation of chronic LBP, a patient with subacute sciatica (pain duration between 6-12 weeks), and a patient with chronic non-specific LBP (pain for longer than 12 weeks). Collectively, the vignettes were modelled to describe a range of factors that could play a role in a doctors' decision-making process to manage LBP (Table 1). GPs were asked to think aloud how they would manage the cases described in the vignettes.(13) For each clinical decision made by the participant, the researcher facilitating the interview asked participants to elaborate on a point, or to obtain clarification if needed (Supplementary file 2).

Analysis

Interview data were analysed using two approaches. We used content analysis to create a summary of GP's choices of pain medicines prescribed for each vignette. Content analysis combines both qualitative and quantitative methods, allowing both the content and frequency of categories to be reported.(14, 15) Two researchers initially reviewed and familiarised themselves with the transcripts, and coded all pain medicines using the following framework: medicines that would be prescribed as first, second, third, or fourth-line care, medicines that GPs would conditionally prescribe (e.g. depending on a patient characteristic), and those that GPs were against prescribing.

We used framework analysis to investigate factors that influence GP's decision-making process.(16) Two researchers coded 10 interview transcripts inductively, developed a library of codes in an iterative process, decided on a coding framework (comprising the themes) and applied it to all interviews. Codes, sub-themes, and themes were reviewed by us, and changes were made as stronger patterns linking codes to themes emerged from the data.(17) Both researchers have a background in physiotherapy and have published on the quality use of pain medicines.(18-20) Data saturation was reached (i.e. no new themes emerged).

Patient and public involvement

For peer review only - http://bmjopen.bmj.com/site/about/guidelines.xhtml

It was not appropriate or possible to involve patients or the public in the design, or conduct, or reporting, or dissemination plans of our research.

RESULTS

We interviewed 25 GPs from April to June, 2022. Most were males (64%) practising in metropolitan areas across six states of Australia. Their mean (SD) age was 53.4 (9.1), and they had a mean of 24.5 (9.3) years of practice as GPs. All but three worked full-time in private practices (Table 2). The recoding of one interview was mistakenly interrupted just before vignette #3 started and therefore there are only data from 24 GPs for that vignette.

Content analysis

Paracetamol and NSAIDs were commonly prescribed for all three vignettes. However, opioid analgesics were the pain medicines most prescribed or conditionally prescribed for the acute exacerbation of chronic LBP and sciatica vignettes, and antidepressants for the chronic LBP vignette. Codeine was the most prescribed opioid analgesic for the acute exacerbation vignette, whereas tramadol was the most common choice for the sciatica vignette. Concerns over the safety of opioids, particularly codeine, were commonly reported by GPs across all vignettes. Those concerns were often specific to a type of opioid, as many who expressed concerns over one type of opioid would still prescribe a different opioid analgesic or conditionally prescribe the same opioid. For example, this was observed in the interviews of 11 (44%) GPs in the acute exacerbation vignette (Table 3).

Anticonvulsants were the medicine class with the highest number of GPs indicating they would not prescribe for the acute exacerbation vignette: nine (36%) were against prescribing them because the patient did not have signs of neuropathic pain that would justify their use. In contrast, 16 (64%) of GPs recommended an anticonvulsant for the sciatica vignette, mostly pregabalin. GPs prescribed antidepressants for all three vignettes, more commonly for the chronic LBP vignette (n=15, 63%). Of those, most chose duloxetine (n=10, 42%) and/or amitriptyline (n=7, 29%). GPs prescribed antidepressants for different reasons for each vignette: co-morbid depression influenced decision-making for the chronic LBP vignette, whereas neuropathic pain was the key reason leading to antidepressants being prescribed for the sciatica vignette.

Factors influencing GP's decision-making to prescribe pain medicines for LBP

We identified 5 key themes that influenced GP's decision-making. Themes, subthemes, and supporting quotes are presented in table 4.

Theme 1: Prioritising personal information sources

Subtheme 1 - Previous experiences: Past clinical and personal experiences were recognised by most GPs as the most important source of information to help them make decisions. Some acknowledged that accumulated clinical experience contributed to the formulation of shortcuts that guided decision-making. Meaningful past experiences were often centred

For peer review only - http://bmjopen.bmj.com/site/about/guidelines.xhtml

around success using medicines with patients, but also personal success when they experienced pain themselves.

GPs considered patients' previous treatment experience an important source of information to assist with their decision-making. Giving patients options and discussing those options with them was often mentioned. However, in those circumstances, patient preferences and values appeared to be the key drivers of decision-making. Discussion about the evidence-base for each option was less prominent.

Subtheme 2 - Guidelines are unhelpful: Many GPs reported that guidelines were not helpful. Guidelines were noted to be particularly unhelpful when patients required more than minimal intervention such as advice and simple analgesics. Recommended interventions such as advice to stay active and education about the benign nature of most LBP were seen as problematic as it could be hard to convince patients in pain of their usefulness. One GP noted that the problem with guidelines was not necessarily their recommendations, but the time required to implement them in a context of GPs pressured for time. Clinical practice guidelines for LBP were not mentioned. Only the *Therapeutic Guidelines(21)* were mentioned by a limited number of GPs.

Subtheme 3 - Clinical training: While the applicability of guidelines was questioned, continuing education activities run by specialists and opinion leaders were highly valued. By comparison, training programs were rarely mentioned; only one GP with five years of experience mentioned the role of medical school and GP registrar training in addressing the current overuse use of opioids for LBP management.

Theme 2: Strong reliance on a biomedical model to guide prescription

Subtheme 1 - Pathoanatomical diagnosis: There was a strong sense amongst GPs that a pathoanatomical diagnosis was key to inform the choice of pain medicine to be prescribed. This was more pronounced for the chronic LBP vignette where, according to GPs, there was a more uncertain clinical presentation due to the description and duration of symptoms as well as comorbidities. Many GPs reported they would have referred the patient for imaging, and many considered the number and nature of imaging findings a key indicator of severity of the problem, which they linked to the prescription of stronger analgesics.

Subtheme 2 - Sleep patterns: Sleep influenced decision-making across the three vignettes, even though the patient in the chronic LBP vignette had no night pain or sleep disturbance. Improving sleep was an important treatment goal in the acute exacerbation vignette. For some, improving sleep was seen as the primary goal of treatment, and often led GPs to prescribe an opioid analgesics or antidepressants, or both. Advice on sleep hygiene was not mentioned.

Subtheme 3 - Pain severity: There were opposing views on the role of pain severity as a factor that influenced decision-making. While some were happy to consider stronger analgesics when pain was more severe, for others pain severity would not prompt immediate escalation to stronger analgesics. GPs were more inclined to consider escalating to stronger analgesics when discussing the acute exacerbation vignette and less inclined to do so in the

For peer review only - http://bmjopen.bmj.com/site/about/guidelines.xhtml

chronic LBP vignette (the average pain intensity was the same in both). For some GPs who would not consider escalating to stronger analgesics, their initial choice of treatment was typically a weak opioid analgesic such as codeine. Those preferring codeine mentioned being more used to the prescribing it than other opioid analgesics.

Subtheme 4 – Comorbidities: Comorbidities described in the vignettes were noted by most and influenced decision-making for some. Losing weight was often mentioned as a treatment goal in all three vignettes and led some GPs to preferring non-pharmacological approaches first or avoiding pharmacological treatments known to cause weight gain (e.g. antidepressants), despite obesity only being mentioned in the acute exacerbation vignette. The history of mild depression described in the chronic LBP vignette led many GPs to consider an antidepressant. The absence of depression in the subacute sciatica vignette was an important factor for GPs when deciding between different pharmacological options for neuropathic pain symptoms. Many acknowledged the role of antidepressants (eg duloxetine, amitriptyline) as potentially effective for symptoms described in the sciatica vignette, but most preferred anticonvulsants as the patient was young, otherwise healthy, physically active, and did not have chronic pain. In contrast, most GPs preferred to prescribe duloxetine for patients with comorbid depression and chronic pain such as the patient described in chronic LBP vignette.

Theme 3: Social factors as root causes

Subtheme 1 - Pain medicines do not address the root cause of the problem: Many GPs reported that prescribing medicines for pain for the acute exacerbation vignette would not be likely to address the root cause of the problem. Some suggested non-pharmacological treatments such as lifestyle interventions, but most GPs expressed concern about the level of social and family support that the patient had and its potential negative impact on her LBP.

Subtheme 2 - Psychosocial-based prescribing: Some GPs considered the patient's social circumstances when deciding whether to prescribe a pain medicine and what to prescribe. In the acute exacerbation vignette, many thought that the patient's social circumstances were important contributors to her pain but from different perspectives. While some emphasised the physical strain related to her carer role as a contributor to the pain, others recognised the psychological consequences of the patient's social circumstances. Those who acknowledged physical factors preferred recommending non-pharmacological options such as ergonomic devices, whereas those who recognised the psychological toll of the patient's carer role more commonly prescribed opioid analgesics or antidepressants. Other GPs were wary about whether side effects from opioid analgesics would hinder the patient's ability to care for her mother due to potential side effects, such as sedation.

Theme 4: Judicious use of pain medicines

Subtheme 1 - Keep it simple or make it complex? There were opposing views about judicious use of pain medicines. Many GPs followed the principles of the WHO analgesic ladder and avoided polypharmacy, particularly for the acute exacerbation vignette, and, to a lesser extent, the sciatica vignette. For GPs following that approach, there was a perception that the clinical course of both vignettes would be favourable and keeping prescription of pain

For peer review only - http://bmjopen.bmj.com/site/about/guidelines.xhtml

medicines to a minimum, combined with non-pharmacological measures, would be effective. Chronic pain and the history of depression were seen as barriers to improvement in the chronic LBP vignette – and therefore to a perception that the patient with chronic LBP would have been more complex to manage.

Other GPs reported that they preferred prescribing multiple pain medicines in combination, at least in the short-term, and most commonly in the sciatica and chronic LBP vignettes. In the sciatica vignette, this was driven by two factors: the notion that there was a well-established mechanism of injury (as opposed to a degenerative process with no known trigger as in the acute exacerbation vignette), and the perception that the clinical presentation was more serious as the patient had radicular symptoms. Both factors led GPs to consider stronger pain medicines such as opioid analgesics and anticonvulsants. In the chronic LBP vignette, polypharmacy often included the prescription of simple analgesics with antidepressants, most commonly duloxetine, which was driven mainly by the history of mild depression.

Theme 5: Concerns about prescription

Subtheme 1 - Drug seeking behaviour and risk of long-term misuse: A key concern for many GPs was drug seeking behaviour. GPs used multiple strategies to identify drug seeking behaviours, such as a review of medical history and intuition. Some were reluctant to prescribe opioid analgesics to patients who were not their regular patients. Some GPs mentioned being able to identify certain stereotypes more likely to be drug seekers. Although opioids were commonly considered by GPs across the three vignettes, most had concerns about their potential for misuse and addiction, particularly in relation to the chronic LBP vignette. Chronic pain and a history of depression were seen as risk factors to long-term misuse and addiction to opioid analgesics.

Subtheme 2 - Side effects of prescription pain medicines: Similarly, most concerns about side effects were related to opioid analgesics. Many GPs expressed concerns about the safety profile of specific opioid analgesics, most commonly codeine, and made decisions about which opioid analgesic to use based on their knowledge about the safety profile of opioid analgesics (e.g., sedation, constipation, nausea). In contrast, very few GPs expressed concerns about other pain medicines with potential for misuse and abuse, such as pregabalin.

Subtheme 3 - Stigma around prescription: Some GPs were aware of the stigma around prescribing pain medicines such as antidepressants. Although frequently mentioned as a treatment option for the sciatica and chronic LBP vignettes, some had concerns that prescribing antidepressants had potential for misinterpretation by patients – for example, that they were implying that the patient's pain was psychological. Lack of time during a regular consultation was seen as important barrier for prescribing antidepressants. There was a perception that longer consultations would be required to explain why the medication was being prescribed to mitigate any concerns from patients.

Subtheme 4 - Pressure from regulators: Some GPs were concerned about being pressured by regulators to not prescribe pain medicines such as opioid analgesics. Their concerns included the potential consequences of undertreating pain. No GP mentioned that pressure from regulators had changed their prescribing behaviour.

For peer review only - http://bmjopen.bmj.com/site/about/guidelines.xhtml

DISCUSSION

GPs in this study made decisions about what pain medicines to prescribe for LBP largely based on previous experiences, including their own clinical and personal experience of LBP management, not guidelines. The choice of pain medicine was influenced by a range of clinical factors, more commonly the patient's pathoanatomical diagnosis. While many adhered to principles of pain medicines prescribing, polypharmacy scenarios were also common. Concerns about drug seeking behaviour, side effects, and stigma around prescription also shaped GPs' decision-making process.

Antidepressants were considered by GPs in our study when they thought depression was linked to the patient's symptoms or as a pain medicine to treat neuropathic pain. Although there is some evidence that antidepressants may be effective for those with chronic pain and comorbid depression and neuropathic pain,(20) the role of antidepressants for chronic LBP is limited and the evidence for sciatica is inconclusive.(18) Some GPs voiced concerns about stigma around prescribing antidepressants for pain that was not observed for any other pain medicine in our study. Stigma around the use of antidepressants is a well-recognised phenomenon in the mental health field, where studies have shown that people using antidepressants for depression have a perception that their condition is more severe and that they are weak or unable to cope with their problems.(22) GPs in our study had the perception that patients may not receive the idea of being prescribed an antidepressant for their pain well.

While our GPs were aware of the harms and potential for misuse and abuse of opioid analgesics, fewer mentioned concerns with other prescription pain medicines such as anticonvulsants, which also have potential for misuse, addiction, and death.(23) The apparent lack of awareness about the harms of these medicines appears to also be common amongst the public.(24) In contrast, public health entities and government regulatory agencies have recognised the concerning potential harms of these medicines and have taken action to reduce them. Such concerns have led regulatory agencies in the UK and Australia to up schedule and to add Boxed Warnings to the Product Information and Consumer Medicine Information for pregabalin, respectively.(23, 25)

Time constraints in general practice are linked to poor adherence to guidelines, inappropriate prescription, and provision of worse care.(26-28) Furthermore, meeting guideline recommendations in primary care requires an unrealistic amount of time that GPs do not have.(29) In line with these findings, there was a perception amongst some GPs that the time required to implement guideline recommendations into practice, or to discuss prescription of certain pain medicines (e.g. antidepressants), was an important barrier to their prescription. Several studies have pointed out that GPs perceive it as nearly impossible to meet all guideline recommendations as there are too many recommendations and most are complex. For example, implementing and documenting all recommendations from guidelines for common problems seen in general practice would take up to 27 hours per day.(30) LBP guidelines involve a complex series of steps from triage to management, and may also take considerable time to be implemented.(31)

For peer review only - http://bmjopen.bmj.com/site/about/guidelines.xhtml

Our findings provide insights for future research to improve the quality use of medicines for LBP. For example, GPs in our study rarely made decisions based on guidelines or evidence. Rather, they strongly relied on previous personal experience with pain medications, and interactions with patients, colleagues, and opinion leaders. Our observations are in line with previous work that has shown that GPs make decisions mainly based on interaction with others rather than endorsed guidelines – a process known as '*mindlines*'.(32)

There are opportunities to develop educational interventions to address some of the factors that influenced decision-making that do not rely on guideline dissemination and consider the resources and sources of information most valued by GPs. One such factor is the inaccurate belief that certain pathoanatomic diagnoses on their own should guide the decision to prescribe certain pain medicines. Pathoanatomic findings are often incidental and have been shown to be mostly unrelated to the development, or severity of symptoms.(33) Interventions could also be developed to increase GPs' awareness of their own biases and heuristics as identified by our study.

The Australian Commission on Safety and Quality in Health Care has recently launched a Clinical Care Standard for LBP to improve care for LBP. One of its core messages is around the quality use of medicines. Specifically, they recommend against anticonvulsants, benzodiazepines, and antidepressants, and that clinicians only consider opioid analgesics for carefully selected patients at the lowest dose for the shortest duration possible.(34) Strategies to implement the Clinical Care Standard may need to account for the perception amongst GPs that following guidelines may lead to patients being undertreated. Our study showed that GPs value opinion leaders as a source of information, which suggests that implementation strategies to improve the quality use of medicines for LBP could involve opinion leaders, an approach that has been shown to improve compliance with evidence-based care.(35)

A strength of our study is the use of vignettes, a widely used methodology for assessing decision-making.(36) Vignettes provided a concrete starting point so that GPs could base their decisions on a detailed patient case, rather than answering abstract questions about attitudes and perceptions. However, the decision-making processes described in our study were anchored to the information provided in the vignettes and may not necessarily be generalisable to all LBP cases or truly reflect objective prescription data.(37) For example, while our study did show that GPs would most commonly prescribe or conditionally prescribe opioids for an acute exacerbation of chronic LBP (vignette 1) or subacute sciatica (vignette 2), which agrees with Australian data on prescription medicines for LBP,[3] our finding that GPs would more commonly prescribe antidepressants for the patient with chronic LBP in vignette 3 differs from existing data. It is unknown whether this difference may be due to the characteristics of the vignette and reflective of practice for patients with the characteristics described in that vignette. Our sample was composed mostly of very experienced GPs practising in metropolitan areas. Their views may differ from those in earlier stages of their careers. For example, the youngest GP in our sample mentioned the role of University and GP training in teaching GPs about the lack of effectiveness and harms of

For peer review only - http://bmjopen.bmj.com/site/about/guidelines.xhtml

opioids for LBP. That view was not shared by any other experienced GP, which could indicate recent changes in teaching curricula. GPs practising in rural areas are more likely to prescribe opioid analgesics for LBP.(3) However, our sample lacked GPs practising in those areas, limiting our ability to contrast potential differences in drivers of decision-making between those areas.

CONCLUSIONS

We identified several aspects of decision-making that help explain the current profile of pain medicines prescribed for LBP in by GPs. Themes identified by our study could inform future implementation strategies to improve the quality use of medicines for LBP.

CONTRIBUTORS

Conceptualisation: GF, JA, CWC, BR, CN, CAS, AM, RD, CM; Methodology: GF, JA; Validation: GF, JZ; Formal analysis: GF, JZ; Investigation: GF, JZ, CJ; Resources: GF; Data curation: GF; Supervision: CM; Project administration: GF; Funding acquisition: GF, CWC, CAS, BR, AM, RD, CM; Writing – Original draft: GF; Writing – Review & editing: JZ, JA, CJ, CWC, BR, CN, CAS, AM, RD, CM

TRANSPARENCY

The lead author (GF) affirms that this manuscript is an honest, accurate, and transparent account of the study being reported; that no important aspects of the study have been omitted; and that any discrepancies from the study as planned (and, if relevant, registered) have been explained.

FUNDING

This study was partially funded by a seed grant from the Australian New Zealand Musculoskeletal Clinical Trials Network (ANZMUSC). GEF, JZ, JA, C-WC L, and CM are supported by National Health and Medical Research Council (NHMRC) fellowships (APP2009808, APP1194105, APP2017278, APP1193939, and APP11094283, respectively). CM is also supported by Centre for Research Excellence grants (APP1134856, APP1171459, APP2006545).

COMPETING INTERESTS DECLARATION

The Sydney Pharmacy School receives funding from GlaxoSmithKline for a postgraduate scholarship supervised by AM. CM has received research grants from various government and not for profit agencies. Flexeze provided heat wraps at no cost for the SHaPED trial for which CM, BR, and CN are investigators. Other authors have no interests to declare.

ETHICAL APPROVAL

The University of Sydney Human Research Ethics Committee approved this research (approval number: 2022/170).

DATA AVAILABILITY

Data are available upon request.

DATA SOURCE

Data collected by the investigators.

LICENSE STATEMENT

For peer review only

The corresponding author (GF) has the right to grant and does grant on behalf of all authors of the Work (as defined in the below author licence), an exclusive licence and/or a non-exclusive licence for contributions from authors who are: i) UK Crown employees; ii) where BMJ has agreed a CC-BY licence shall apply, and/or iii) in accordance with the terms applicable for US Federal Government officers or employees acting as part of their official duties; on a worldwide, perpetual, irrevocable, royalty-free basis to BMJ Publishing Group Ltd ("BMJ") its licensees and where the relevant Journal is co-owned by BMJ to the co-owners of the Journal, to publish the Work in BMJ Quality & Safety and any other BMJ products and to exploit all rights, as set out in our licence.

The corresponding author (GF) accepts and understands that any supply made under these terms is made by BMJ to the Submitting Author unless you are acting as an employee on behalf of your employer or a postgraduate student of an affiliated institution which is paying any applicable article publishing charge ("APC") for Open Access articles. Where the Submitting Author wishes to make the Work available on an Open Access basis (and intends to pay the relevant APC), the terms of reuse of such Open Access shall be governed by a Creative Commons licence – details of these licences and which Creative Commons licence will apply to this Work are set out in our licence referred to above.

REFERENCES

1. Australian Institute of Health and Welfare. Back problems. Canberra: AIHW; 2020.
2. Williams CM, Maher CG, Hancock MJ, McAuley JH, McLachlan AJ, Britt H, et al. Low back pain and best practice care: A survey of general practice physicians. Arch Intern Med. 2010;170(3):271-7.
3. Mathieson S, Valenti L, Maher CG, Britt H, Li Q, McLachlan AJ, et al. Worsening trends in analgesics recommended for spinal pain in primary care. Eur Spine J. 2018;27(5):1136-45.
4. NICE. Low back pain and sciatica in over 16s: assessment and management 2020 [Available from: https://www.nice.org.uk/guidance/ng59.
5. Enke O, New HA, New CH, Mathieson S, McLachlan AJ, Latimer J, et al. Anticonvulsants in the treatment of low back pain and lumbar radicular pain: a systematic review and meta-analysis. Cmaj. 2018;190(26):E786-e93.
6. Mathieson S, Maher CG, McLachlan AJ, Latimer J, Koes BW, Hancock MJ, et al. Trial of Pregabalin for Acute and Chronic Sciatica. New England Journal of Medicine. 2017;376(12):1111-20.
7. Ivanova JI, Birnbaum HG, Schiller M, Kantor E, Johnstone BM, Swindle RW. Real-world practice patterns, health-care utilization, and costs in patients with low back pain: the long road to guideline-concordant care. Spine J. 2011;11(7):622-32.
8. Gouveia N, Rodrigues A, Ramiro S, Eusébio M, Machado PM, Canhão H, et al. The Use of Analgesic and Other Pain-Relief Drugs to Manage Chronic Low Back Pain: Results from a National Survey. Pain Practice. 2017;17(3):353-65.
9. Britt H MG, Bayram C, Henderson J, Valenti L, Harrison C, Pan Y, Charles J,, Pollack AJ CT, Gordon J, Wong C. A decade of Australian general practice activity 2006–07 to 2015–16. Sydney: Sydney University Press, 2016; 2016.
10. Bishop FL, Dima AL, Ngui J, Little P, Moss-Morris R, Foster NE, et al. "Lovely Pie in the Sky Plans": A Qualitative Study of Clinicians' Perspectives on Guidelines for Managing Low Back Pain in Primary Care in England. Spine (Phila Pa 1976). 2015;40(23):1842-50.
11. Tong A, Sainsbury P, Craig J. Consolidated criteria for reporting qualitative research (COREQ): a 32-item checklist for interviews and focus groups. Int J Qual Health Care. 2007;19(6):349-57.
12. Jensen RK, Kongsted A, Kjaer P, Koes B. Diagnosis and treatment of sciatica. BMJ. 2019;367:l6273.
13. Lundgren-Laine H, Salantera S. Think-aloud technique and protocol analysis in clinical decision-making research. Qual Health Res. 2010;20(4):565-75.
14. O'Keeffe M, Michaleff ZA, Harris IA, Buchbinder R, Ferreira GE, Zadro JR, et al. Public and patient perceptions of diagnostic labels for non-specific low back pain: a content analysis. European Spine Journal. 2022;31(12):3627-39.

For peer review only - http://bmjopen.bmj.com/site/about/guidelines.xhtml

15. Ferreira GE, Zadro J, O'Keeffe M, Chen M, Gao Y, Hu Z, et al. A content analysis of online information about the benefits and harms of spine surgery. Braz J Phys Ther. 2022;26(2):100398.
16. Gale NK, Heath G, Cameron E, Rashid S, Redwood S. Using the framework method for the analysis of qualitative data in multi-disciplinary health research. BMC Medical Research Methodology. 2013;13(1):117.
17. Ayre J, McCaffery KJ. Research Note: Thematic analysis in qualitative research. J Physiother. 2022;68(1):76-9.
18. Ferreira GE, McLachlan AJ, Lin CC, Zadro JR, Abdel-Shaheed C, O'Keeffe M, et al. Efficacy and safety of antidepressants for the treatment of back pain and osteoarthritis: systematic review and meta-analysis. BMJ. 2021;372:m4825.
19. Ferreira GE, Machado GC, Abdel Shaheed C, Lin CC, Needs C, Edwards J, et al. Management of low back pain in Australian emergency departments. BMJ Qual Saf. 2019;28(10):826-34.
20. Ferreira GE, Abdel-Shaheed C, Underwood M, Finnerup NB, Day RO, McLachlan A, et al. Efficacy, safety, and tolerability of antidepressants for pain in adults: overview of systematic reviews. BMJ. 2023;380:e072415.
21. Therapeutic Guidelines. Melbourne: Therapeutic Guidelines Limited; [Available from: https://tg.org.au.
22. Interian A, Martinez IE, Guarnaccia PJ, Vega WA, Escobar JI. A qualitative analysis of the perception of stigma among Latinos receiving antidepressants. Psychiatr Serv. 2007;58(12):1591-4.
23. Mathieson S, Lin CC, Underwood M, Eldabe S. Pregabalin and gabapentin for pain. BMJ. 2020;369:m1315.
24. Mathieson S, O'Keeffe M, Traeger AC, Ferreira GE, Abdel Shaheed C. Content and sentiment analysis of gabapentinoid-related tweets: An infodemiology study. Drug and Alcohol Review. 2022;n/a(n/a).
25. Therapeutic Goods Administration. Pregabalin and gabapentin. Safety advisory - enhanced warnings relating to abuse and dependence 2021 [Available from: https://www.tga.gov.au/news/safety-alerts/pregabalin-and-gabapentin.
26. Tamblyn R, Berkson L, Dauphinee WD, Gayton D, Grad R, Huang A, et al. Unnecessary prescribing of NSAIDs and the management of NSAID-related gastropathy in medical practice. Ann Intern Med. 1997;127(6):429-38.
27. Campbell SM, Hann M, Hacker J, Burns C, Oliver D, Thapar A, et al. Identifying predictors of high quality care in English general practice: observational study. BMJ. 2001;323(7316):784.
28. Tsiga E, Panagopoulou E, Sevdalis N, Montgomery A, Benos A. The influence of time pressure on adherence to guidelines in primary care: an experimental study. BMJ Open. 2013;3(4):e002700.
29. Privett N, Guerrier S. Estimation of the Time Needed to Deliver the 2020 USPSTF Preventive Care Recommendations in Primary Care. Am J Public Health. 2021;111(1):145-9.
30. Porter J, Boyd C, Skandari MR, Laiteerapong N. Revisiting the Time Needed to Provide Adult Primary Care. J Gen Intern Med. 2023;38(1):147-55.
31. Bardin LD, King P, Maher CG. Diagnostic triage for low back pain: a practical approach for primary care. Med J Aust. 2017;206(6):268-73.
32. Gabbay J, le May A. Evidence based guidelines or collectively constructed "mindlines?" Ethnographic study of knowledge management in primary care. Bmj. 2004;329(7473):1013.
33. Kasch R, Truthmann J, Hancock MJ, Maher CG, Otto M, Nell C, et al. Association of Lumbar MRI Findings with Current and Future Back Pain in a Population-based Cohort Study. Spine. 2022;47(3).
34. Australian Commission on Safety and Quality in Health Care.Low Back Pain Clinical Care Standard. Sydney: ACSQHC; 2022.
35. Flodgren G, O'Brien MA, Parmelli E, Grimshaw JM. Local opinion leaders: effects on professional practice and healthcare outcomes. Cochrane Database Syst Rev. 2019;6(6):Cd000125.
36. Evans SC, Roberts MC, Keeley JW, Blossom JB, Amaro CM, Garcia AM, et al. Vignette methodologies for studying clinicians' decision-making: Validity, utility, and application in ICD-11 field studies. Int J Clin Health Psychol. 2015;15(2):160-70.
37. Alexander CS, Becker HJ. The Use of Vignettes in Survey Research. Public Opinion Quarterly. 1978;42(1):93-104.

For peer review only - http://bmjopen.bmj.com/site/about/guidelines.xhtml

Table 1. Features of each clinical vignette.

	Vignette 1	Vignette 2	Vignette 3
**Patient characteristics**			
Female	X		X
Male		X	
Middle-aged	X	X	X
Physical activity			
No physical activity	X		
Insufficient physical activity			X
Physically active		X	
**Social aspects**			
Children			X
Primary carer for older person	X		
**Type of low back pain**			
Atraumatic back pain	X	X	X
Back pain initiated after a well-defined event		X	
Back pain with a clear radicular component		X	
Back pain with referred leg pain			X
**Pain characteristics**			
Night pain	X	X	
Sleep disturbance	X		
Back pain exacerbated by movements/postures		X	X
Acute-on-chronic pain	X		
Sub-acute (pain between 6 and 12 weeks)		X	
Chronic (pain > 12 weeks)			X
Movement restriction	X	X	
Limitations to work		X	
**Comorbidities**			
Obesity	X		
Gastro-oesophageal reflux disease	X		
High cholesterol	X		
Other musculoskeletal conditions	X		
Diabetes			X
Depression (mild)			X
**Healthcare use**			
General practitioner for low back pain		X	
Physiotherapy for low back pain			X
Acupuncture for low back pain			X
Psychologist for mild depression			X
Previous imaging	X		
Previous/current use of pain medicines	X	X	X
Paracetamol, NSAIDs	X		X
Opioids		X	

For peer review only - http://bmjopen.bmj.com/site/about/guidelines.xhtml

Table 2. Characteristics of participants (n = 25)

Age, mean (SD)	53.4 (9.1)
Sex, n (%)	
Female	9 (36%)
Male	16 (64%)
State/territory	
New South Wales	9 (36%)
Queensland	6 (24%)
Victoria	5 (20%)
South Australia	2 (8%)
Western Australia	2 (8%)
Tasmania	1 (4%)
Remoteness[1]	
Metropolitan	22 (88%)
Rural/remote	3 (12%)
Number of patients with low back pain seen per year, n (%)	
5-25	2 (8%)
26-50	4 (16%)
51-100	3 (12%)
>100	16 (64%)
Years of experience, n (SD) [min – max]	24.6 (9.3) [5 – 40]
Weekly clinical workload (hours), mean (SD) [min – max]	38 (7.7) [14 – 50]

[1]Defined per the Australian Statistical Geography Standard; GPs working in metropolitan areas are those whose practice is located in a Greater Capital City Statistical Area.

For peer review only

Table 3. Pain medicines (n, %) that GPs would prescribe, would conditionally prescribe, were against prescribing, or that were not mentioned for each vignette.

	Paracetamol	NSAIDs	Opioids	Antidepressants	Anticonvulsants	Oral steroids	Benzodiazepines
**Vignette 1 (n = 25)**							
Would prescribe	13 (52)	17 (68)	8 (32)	6 (24)	1 (4)	0 (0)	0 (0)
Would conditionally prescribe	0 (0)	3 (12)	14 (56)	3 (12)	0 (0)	1 (4)	3 (12)
Against prescribing	0 (0)	2 (8)	2 (8)	6 (24)	9 (36)	1 (4)	1 (4)
Not mentioned	12 (48)	3 (12)	1 (4)	10 (40)	15 (60)	23 (92)	21 (84)
**Vignette 2 (n = 25)**							
Would prescribe	8 (32)	16 (64)	7 (28)	6 (24)	11 (44)	3 (12)	0 (0)
Would conditionally prescribe	0 (0)	0 (0)	12 (48)	7 (28)	5 (20)	2 (8)	0 (0)
Against prescribing	0 (0)	2 (8)	6 (24)	3 (12)	5 (20)	3 (12)	1 (4)
Not mentioned	17 (68)	7 (28)	0 (0)	9 (36)	4 (16)	17 (68)	24 (96)
**Vignette 3 (n = 24)***							
Would prescribe	8 (33)	8 (33)	8 (33)	10 (42)	5 (21)	0 (0)	1 (4)
Would conditionally prescribe	1 (4)	1 (4)	3 (12)	5 (21)	2 (8)	0 (0)	0 (0)
Against prescribing	0 (0)	2 (8)	4 (17)	0 (0)	1 (4)	2 (8)	0 (0)
Not mentioned	15 (62)	13 (54)	9 (37)	9 (37)	16 (67)	22 (92)	23 (96)

GP, General practitioner; NSAIDs, non-steroidal anti-inflammatories

*Data from 24 GPs shown. The interview with one GP discussing vignette 3 was not recorded due to technical issues.

For peer review only - http://bmjopen.bmj.com/site/about/guidelines.xhtml

Table 4. Themes, subthemes, and supporting quotes.

Themes	Subthemes	Supporting quotes
Prioritising personal information sources	Previous experience	• As an experienced GP of just over 20 years, you have scripts in your mind that bring you to a sort of automatic pilot approach. (GP #20, male, 21 years of experience)<br>• I'd probably just give her the options of weather she wants some anti-inflammatory, something with codeine, and antidepressant. (GP #3, female, 23 years of experience)<br>• I've had radicular pain myself, and the only thing that fixed me was Lyrica. I actually had little faith in Lyrica before I used it, but nothing else touched my pain. A lot of my approach to radicular pain is based on my own experience. (GP #1, female, 30 years of experience)
	Guidelines are unhelpful	• Sometimes I find guidelines are not actually practical. If they say 'oh tell the patient they need to keep fit and tell them that pain is not a bad sign and that they should keep active and a little bit of pain is ok', it can be hard to convince patients of that. (GP #1, female, 30 years of experience)
	Clinical training	• Probably what I've heard at specialist meetings when the specialists have spoken. I went to one last night and the neurosurgeon was basically saying you try and avoid going on the path of long-acting opioids, and you can use a quick acting opioid. (GP #6, female, 35 years of experience).<br>• Part of it is what we what we've learned through GP registrar training and medical school, where there's a strong emphasis on not using opioid painkillers in for the management of back pain (GP #17, male, 5 years of experience)
Strong reliance on a biomedical model to guide prescription	Pathoanatomical diagnosis	• When we do the imaging, it's going to support our treatment more. If you find one of two nerves there affecting him, we can start him on pregabalin 25 mg (GP #23, female, 28 years of experience)<br>• If we found multiple levels of a minor disc bulge or something, I would be happier to give her something stronger, but if the MRI just showed that she had a disc bulge at one level and everything else looks fine, there's no nerve impingement, I don't think that would warrant it. I'd be happy to try amitriptyline than other medications as a first line. (GP #9, female, 25 years of experience).
	Sleep patterns	• I'd be happy to give her some panadeine [NB: codeine + paracetamol] straight away because it's keeping her awake at night, and that's always a bit of a problem. She wakes up because of the pain… I mean you don't want people waking up with pain, so give her a bit of mild pain relief. (GP #22, male, 39 years of experience)
	Pain severity	• No because she might still respond to what I've decided to use, it might just be temporary that she's eight or nine [NB: pain out of 10] (GP #4, female, 30 years of experience)
	Comorbidities	• There are some other antidepressants like Cymbalta which can act as a neuropathic agent as well as an antidepressant. So there are antidepressants which we can use which help patients manage their depression as well as well as their pain. (GP #19, male, 30 years of experience)
Social factors as root causes	pain medicines do not address the root cause of the problem	• We don't want her having a fall with her mum and we don't want her experiencing side effects that may affect her ability to care for her mother. (GP #7, male, 18 years of experience)
	Psychosocial-based prescribing	• Since she's getting relief with paracetamol and ibuprofen, I think the next step is to find if there are any associated issues in terms of looking after her mum. Can we get some home help… refer mum to aged care services? I mean she might be able to get NDIS help [NB: government support] as well (GP #11, male, 20 years of experience)

For peer review only - http://bmjopen.bmj.com/site/about/guidelines.xhtml

Judicious use of pain medicines	Keep it simple or make it complex?	• They get flare ups here and there and I think you don't jump up and down as long as you know exactly the origin of the pain and you know you excluded that there is more to it than just what's presenting at this stage, and I think let's just keep it simple. And you can always go back and increase the medication or order further investigations if things don't subside, but I don't hit them harder on the 1st presentation. (GP #15, male, 30 years of experience)<br><br>• I tend to like to use a smaller dose of a lot of things rather than a bigger dose of one or two things. So I'd write a little plan and it would have a sliding scale so it would start off with the Panadol three times a day plus the Nurofen. That's the foundations of the house, and then I would add into that tramadol sustained release 150 to 100 twice a day, and then I would usually add in amitriptyline 10 to 20 milligrams before bed. (GP #25, female, 30 years of experience)
Concerns about prescription	drug seeking behaviour and risk of long-term misuse	• It's a chronic, insidious, ongoing bit of depression. And it just typically… It's just super clean. She gets started on endone and then get stuck on endone and that becomes their whole life. And they just slowly over a course of years dose-escalate. And they feel better. Because of the euphoria associated with the oxycodone. Terrible. (GP #16, male, 34 years of clinical experience)
	Side effects of prescription pain medicines	• Opioids induce hyperalgesia, which comes with a lot of opioids such as codeine, which is a poor analgesic. I don't use that either. I basically use 3 analgesics in my practice: tramadol, buprenorphine, and tapentadol. (GP #19, Male, 30 years of experience)
	Stigma around prescription	• It could be done, but it would involve a very lengthy consultation and we as GPs don't have half an hour to 45 minutes to discuss something a little bit more wayward. (GP #7, male, 18 years of experience)
	Pressure from regulators	• The guidelines at the moment for non-opioid medication are clear as far as we can all see. I don't know how much that'll go the wrong way and we'll end up not treating anybody pains eventually. We get sued for not treating anybody pain and the pain and suffering and next thing you know the guidelines will change, you know? But you can't win, can you? (GP #14, male, 40 years of experience)<br><br>• Department of Health want us not to give pain medicine at all for low back pain but sometimes it's not realistic. Sometimes we need to help the patient even though I mean, potentially they can be additive of course, but, realistically, the patient is in so much pain, they can't move. This is cruel. Not giving opioid medicines, at least for a few days. I'm not the type of person that gives everybody strong pain medicines, but I think there is a role in some patients. (GP #18, male, 27 years of experience)

For peer review only

General practitioners' decision-making process to prescribe pain medicines for low back pain: a qualitative study

Contents

Supplementary file 1. Vignettes 2
Supplementary file 2. Interview guide. 5

For peer review only

Supplementary file 1. Vignettes

Prompt: I will now present to you three clinical vignettes describing different low back pain presentations. After each vignette I am going to ask you a series of questions. Please draw on your prior experience to answer the questions about how you would manage these back pain patients. There are no right or wrong answers. We are interested in honest opinions, not idealised ones (for example, what you should do). Remember that your data will be anonymised.

VIGNETTE 1

- Patient info: Female patient, 57 years old, BMI 33 (height: 1.60m; weight: 85kg)
- Complaint: atraumatic onset low back pain that started 2 weeks ago. Average pain 5/10; worse in the evening/at night with pain 7/10.
- History:
    - Low back pain for 20+ years (ongoing mild pain 2/10) with occasional moderate flares (pain up to 5/10) that typically resolve in a few days. Previous MRIs (last one 3 years ago) showed the following: At L5/S1 there is vertebral endplate bone marrow oedema. The distinction between the nucleus and annulus is lost with a with loss of disc height.
    - She has sought care for back pain a few times before, but typically self-manages the pain with simple painkillers (Paracetamol and occasionally ibuprofen as needed). She is seeking care for this episode because it's taking longer to resolve, and the pain is more intense in the evening/at night
    - Patient is having difficulty sleeping – she wakes up because of pain when turning in bed.
    - Patient is sedentary and is the primary carer of her 88-year-old mother who is bed-bound due to a stroke 12 months ago.
    - Comorbidities: Obesity, GORD, high cholesterol (daily atorvastatin), medial compartment R knee osteoarthritis (also treats with simple analgesics as needed).
- Physical examination:
    - No evidence of radicular symptoms: straight leg raise, sensation, reflexes, and lower limb strength are normal. No signs of infection or midline tenderness in the lumbar spine. Tenderness over the paraspinal muscles is present.
    - Movements in every direction are slightly more painful (6/10)

For peer review only

VIGNETTE 2

- Patient info: 45-year-old male, BMI 26 (height: 1.83m; weight: 87kg)

- Complaint: Acute onset low back pain with shooting stabbing pain radiating down the back of the right leg to the foot and associated with pins and needles in the same area. This commenced 8 weeks ago after trying to lift a heavy box while visiting family in rural NSW. Main complaint is leg pain (5/10), but he also has pain in the lower back (3/10). Sitting for >1h aggravates leg pain (7/10), sit-to-standing and vice-versa are quite painful (7/10).

- History:
  - No history of low back pain or any other musculoskeletal condition prior to this episode. Patient is physically active, swims and runs twice a week. Sitting for more than 1 hour aggravates pain and is severely limiting his ability to work. He has not taken any time off work.
  - Patient went to the local general practitioner the day after pain onset who prescribed codeine 30mg QID for 1 week. No improvement was noted. No significant side effects were noted either besides constipation.
  - Patient has not reported any significant sleep disturbance

- Physical examination:
  - Pain follows a dermatomal distribution (S1), with altered sensation to light touch. Muscle strength and reflexes are preserved.
  - No signs of infection or midline tenderness in the lumbar spine.
  - Straight leg raise positive at ~30deg; crossed straight leg raise test is negative
  - Flexion and extension movements are limited and increase leg pain (7/10). Rotations and inclinations do not change symptoms.
  - Cauda equina syndrome and Other red flags were excluded

For peer review only

VIGNETTE 3

- Patient info: Female patient, 52 years old, BMI 30 (height: 1.63m; weight: 79kg)

- Complaint: Insidious onset low back pain radiating to the L thigh that started 6 months ago. Patient cannot recall an event that triggered the pain. Pain is bilateral in the lower back and radiates to the L posterior thigh with the same average intensity (5/10). Standing up/walking for too long (>1h) increases pain (6-7/10). Sitting/lying down reduces pain. She does not have trouble sleeping.

- History:

  - No history of low back pain or any other musculoskeletal condition prior to this episode. Patient is married with 3 children (youngest is 20 years-old),
  - Patient has recently been diagnosed with type II diabetes (she takes metformin daily and has been going to the gym 3 times/week for about 45 minutes where she does low-moderate intensity resistance training since the diagnosis – 2 years ago. Her sugar levels are currently controlled – around 5mmol/L before meals. Kidney function is normal). She was diagnosed with mild depression two years ago and has been supported by a psychologist with training in cognitive behavioural therapy. No other pharmacological treatments are used for depression.
  - She uses over-the-counter analgesics (Paracetamol and Ibuprofen) regularly on an as-needed basis to manage the pain. She has seen a physiotherapist (core strengthening exercises) and an acupuncturist but has not responded to either treatment.

- Physical examination:
  - No abnormalities on neurological exam (sensation, strength, and reflexes are all normal).
  - No signs of infection or midline tenderness in the lumbar spine.
  - Straight leg raise and crossed Straight leg raise negative.
  - Lumbar spine movements are limited mostly by lack of flexibility. End-range lumbar extension increases pain momentarily (7/10), which reduces as soon as patient returns to neutral position.

For peer review only

Supplementary file 2. Interview guide.

Questions - Choosing between different types of pharmacological treatments

Prompt: Answer the following questions based on the patient vignette you just read. Using the information from the vignette, I would like you to think aloud your clinical reasoning.

- Would you typically prescribe a pain medicine for the patient described in the vignette?

- Could you elaborate on which pain medicines you would prescribe and why you would prescribe them *[remind the participant of the medicines they mentioned]*?

Prompt participant to talk about their rationale to prescribe/recommend certain medicines; what kind of thought process they follow, sources of information that they use to make their decision:

- *Clinical presentation (e.g. affected sleep in vignette 1; neuropathic pain features in vignette 2; depression in vignette 3, etc.)*
- *Severity of symptoms*
- *Known efficacy from trials and systematic reviews*
- *Guideline recommendation*
- *Safety/side effects*
- *Previous use by patient*
- *Previous success with other patients*
- *Demographic factors*
- *Mechanisms of action of the drug*
- *Intuition*

Questions – influence of factors not mentioned by the participant

- How do aspects like (eg age, gender, comorbidities, description of pain, etc.) factor in your decision to prescribe 'ABC' *[the pain medicines mentioned by the participant]*?

Questions - Alternatives

- What about pain medicines 'XYZ'? Would you prescribe any of these for the vignette? *[mention pain medicines that the participant did not mention eg strong opioids [oxycodone endone], weak opioids [codeine], gabapentin, pregabalin, amitriptyline, oral steroids, etc.)]*

- Are there any circumstances where you would prescribe XYZ instead or/in addition to ABC for the patient in this vignette?

Questions – external factors influencing decision-making

- Would your decision-making change if you were pressed for time? *[eg do they report using any heuristic/rules of thumb when they are time-limited?]*
- Would your decision-making change depending on patients' opinions on the treatments you typically prescribe?

For peer review only

COREQ (COnsolidated criteria for REporting Qualitative research) Checklist

A checklist of items that should be included in reports of qualitative research. You must report the page number in your manuscript where you consider each of the items listed in this checklist. If you have not included this information, either revise your manuscript accordingly before submitting or note N/A.

Topic	Item No.	Guide Questions/Description	Reported on Page No.
**Domain 1: Research team and reflexivity**			
Personal characteristics			
Interviewer/facilitator	1	Which author/s conducted the interview or focus group?	
Credentials	2	What were the researcher's credentials? E.g. PhD, MD	
Occupation	3	What was their occupation at the time of the study?	
Gender	4	Was the researcher male or female?	
Experience and training	5	What experience or training did the researcher have?	
Relationship with participants			
Relationship established	6	Was a relationship established prior to study commencement?	
Participant knowledge of the interviewer	7	What did the participants know about the researcher? e.g. personal goals, reasons for doing the research	
Interviewer characteristics	8	What characteristics were reported about the inter viewer/facilitator? e.g. Bias, assumptions, reasons and interests in the research topic	
**Domain 2: Study design**			
Theoretical framework			
Methodological orientation and Theory	9	What methodological orientation was stated to underpin the study? e.g. grounded theory, discourse analysis, ethnography, phenomenology, content analysis	
Participant selection			
Sampling	10	How were participants selected? e.g. purposive, convenience, consecutive, snowball	
Method of approach	11	How were participants approached? e.g. face-to-face, telephone, mail, email	
Sample size	12	How many participants were in the study?	
Non-participation	13	How many people refused to participate or dropped out? Reasons?	
Setting			
Setting of data collection	14	Where was the data collected? e.g. home, clinic, workplace	
Presence of non-participants	15	Was anyone else present besides the participants and researchers?	
Description of sample	16	What are the important characteristics of the sample? e.g. demographic data, date	
Data collection			
Interview guide	17	Were questions, prompts, guides provided by the authors? Was it pilot tested?	
Repeat interviews	18	Were repeat inter views carried out? If yes, how many?	
Audio/visual recording	19	Did the research use audio or visual recording to collect the data?	
Field notes	20	Were field notes made during and/or after the inter view or focus group?	
Duration	21	What was the duration of the inter views or focus group?	
Data saturation	22	Was data saturation discussed?	
Transcripts returned	23	Were transcripts returned to participants for comment and/or	

For peer review only

Topic	Item No.	Guide Questions/Description	Reported on Page No.
		correction?	
**Domain 3: analysis and findings**			
Data analysis			
Number of data coders	24	How many data coders coded the data?	
Description of the coding tree	25	Did authors provide a description of the coding tree?	
Derivation of themes	26	Were themes identified in advance or derived from the data?	
Software	27	What software, if applicable, was used to manage the data?	
Participant checking	28	Did participants provide feedback on the findings?	
Reporting			
Quotations presented	29	Were participant quotations presented to illustrate the themes/findings? Was each quotation identified? e.g. participant number	
Data and findings consistent	30	Was there consistency between the data presented and the findings?	
Clarity of major themes	31	Were major themes clearly presented in the findings?	
Clarity of minor themes	32	Is there a description of diverse cases or discussion of minor themes?	

Developed from: Tong A, Sainsbury P, Craig J. Consolidated criteria for reporting qualitative research (COREQ): a 32-item checklist for interviews and focus groups. *International Journal for Quality in Health Care*. 2007. Volume 19, Number 6: pp. 349 – 357

Once you have completed this checklist, please save a copy and upload it as part of your submission. DO NOT include this checklist as part of the main manuscript document. It must be uploaded as a separate file.

For peer review only

BMJ Open

General practitioners’ decision-making process to prescribe pain medicines for low back pain: a qualitative study

Journal:	*BMJ Open*
Manuscript ID	bmjopen-2023-074380.R3
Article Type:	Original research
Date Submitted by the Author:	24-Sep-2023
Complete List of Authors:	Ferreira, Giovanni; University of Sydney School of Public Health, Institute for Musculoskeletal Health<br>Zadro, Joshua; University of Sydney, Institute for Musculoskeletal Health, School of Public Health<br>Jones, Caitlin; The University of Sydney Institute for Musculoskeletal Health, School of Public Health<br>Ayre, Julie; The University of Sydney, Sydney Health Literacy Lab<br>Lin, Christine; University of Sydney School of Public Health, Institute for Musculoskeletal Health<br>Richards, Bethan; The University of Sydney Institute for Musculoskeletal Health<br>Needs, Christopher; Institute of Rheumatology & Orthopaedics, Rheumatology Department<br>Abdel Shaheed, Christina; University of Sydney School of Public Health, Institute for Musculoskeletal Health<br>McLachlan, Andrew; University of Sydney, Faculty of Pharmacy<br>Day, Richard; St Vincent's Hospital, Department of Clinical Pharmacology & Toxicology; University of New South Wales, St Vincent's Clinical School, Faculty of Medicine<br>Maher, Christopher; University of Sydney, Institute for Musculoskeletal Health, Sydney School of Public Health, Faculty of Medicine and Health
**Primary Subject Heading**:	General practice / Family practice
Secondary Subject Heading:	Qualitative research
Keywords:	Decision Making, Back pain < ORTHOPAEDIC & TRAUMA SURGERY, QUALITATIVE RESEARCH

SCHOLARONE™
Manuscripts

I, the Submitting Author has the right to grant and does grant on behalf of all authors of the Work (as defined in the below author licence), an exclusive licence and/or a non-exclusive licence for contributions from authors who are: i) UK Crown employees; ii) where BMJ has agreed a CC-BY licence shall apply, and/or iii) in accordance with the terms applicable for US Federal Government officers or employees acting as part of their official duties; on a worldwide, perpetual, irrevocable, royalty-free basis to BMJ Publishing Group Ltd ("BMJ") its licensees and where the relevant Journal is co-owned by BMJ to the co-owners of the Journal, to publish the Work in this journal and any other BMJ products and to exploit all rights, as set out in our licence.

The Submitting Author accepts and understands that any supply made under these terms is made by BMJ to the Submitting Author unless you are acting as an employee on behalf of your employer or a postgraduate student of an affiliated institution which is paying any applicable article publishing charge ("APC") for Open Access articles. Where the Submitting Author wishes to make the Work available on an Open Access basis (and intends to pay the relevant APC), the terms of reuse of such Open Access shall be governed by a Creative Commons licence – details of these licences and which Creative Commons licence will apply to this Work are set out in our licence referred to above.

Other than as permitted in any relevant BMJ Author's Self Archiving Policies, I confirm this Work has not been accepted for publication elsewhere, is not being considered for publication elsewhere and does not duplicate material already published. I confirm all authors consent to publication of this Work and authorise the granting of this licence.

For peer review only

General practitioners' decision-making process to prescribe pain medicines for low back pain: a qualitative study

Giovanni E Ferreira[1,2], Joshua R Zadro[1,2], Caitlin Jones[1,2], Julie Ayre[1,2], Chung-Wei Christine Lin[1,2], Bethan Richards[2,4], Christopher Needs[4], Christina Abdel Shaheed[1,2], Andrew McLachlan[5], Richard Day[6], Chris G Maher[1,2]

1. Sydney Musculoskeletal Health, Faculty of Medicine and Health, The University of Sydney
2. Institute for Musculoskeletal Health, The University of Sydney and Sydney Local Health District
3. Sydney Health Literacy Lab, School of Public Health, Faculty of Medicine and Health, The University of Sydney, Sydney, Australia
4. Department of Rheumatology, Royal Prince Alfred Hospital, Sydney, Australia
5. Sydney Pharmacy School, Faculty of Medicine and Health, The University of Sydney, Sydney, Australia
6. St Vincent's Clinical School, Department of Clinical Pharmacology & Toxicology, University of New South Wales, Sydney, Australia

Abstract word count: 291
Manuscript word count: 3989

Address all correspondence to:

Giovanni E. Ferreira
School of Public Health, Faculty of Medicine and Health, The University of Sydney, Sydney, New South Wales, Australia
PO Box M179, Missenden Road, Camperdown | NSW | 2050, Australia
E-mail: giovanni.ferreira@sydney.edu.au

For peer review only - http://bmjopen.bmj.com/site/about/guidelines.xhtml

ABSTRACT

Background: Pain medicines are widely prescribed by general practitioners (GPs) when managing people with low back pain (LBP), but little is known about what drives decisions to prescribe these medicines.

Objectives: The aim of this study was to investigate what influences GPs' decision to prescribe pain medicines for LBP.

Design: Qualitative study with in-depth interviews.

Setting: Australian primary care.

Participants: We interviewed 25 GPs practising in Australia experienced in managing LBP (mean [SD] age 53.4 [9.1] years, mean [SD] years of experience: 24.6 [9.3], 36% female). GPs were provided three vignettes describing common LBP presentations (acute exacerbation of chronic LBP, subacute sciatica, and chronic LBP) and were asked to think aloud how they would manage the cases described in the vignettes.

Data analysis: We summarised GP's choices of pain medicines for each vignette using content analysis and used framework analysis to investigate factors that affected GP's decision-making.

Results: GPs more commonly prescribed opioid analgesics. Anticonvulsants and antidepressants were also commonly prescribed depending on the presentation described in the vignette. GP participants made decisions about what pain medicines to prescribe for LBP largely based on previous experiences, including their own personal experiences of LBP, rather than guidelines. The choice of pain medicine was influenced by a range of clinical factors, more commonly the patient's pathoanatomical diagnosis. While many adhered to principles of judicious use of pain medicines, polypharmacy scenarios were also common. Concerns about drug seeking behaviour, adverse effects, stigma around opioid analgesics, and pressure from regulators also shaped their decision-making process.

Conclusions: We identified several aspects of decision-making that help explain the current profile of pain medicines prescribed for LBP by GPs. Themes identified by our study could inform future implementation strategies to improve the quality use of medicines for LBP.

Keywords: decision-making, opioids, low back pain, prescription, sciatica

For peer review only - http://bmjopen.bmj.com/site/about/guidelines.xhtml

Strengths and limitations of the study

- We used clinical vignettes resembling common low back pain cases to provide clinicians with a concrete starting point where they could base their decisions on
- Voices and perspectives of younger general practitioners or general practitioners working in rural and remote areas may have been underrepresented.

For peer review only

For peer review only - http://bmjopen.bmj.com/site/about/guidelines.xhtml

INTRODUCTION

Low back pain (LBP) affects over 4 million Australians every year and pain medicines are widely used in its management.(1) Australian data shows that 2 in every 3 patients who seek primary care are prescribed or recommended at least one pain medicine.(2) Examples include over-the-counter analgesics (e.g. paracetamol, and non-steroidal anti-inflammatories (NSAIDs)) and prescription medicines such as opioid analgesics, gabapentinoids, antidepressants, and muscle relaxants.(3)

There is a mismatch between the pharmacological therapies recommended in clinical practice guidelines for LBP and what is prescribed or recommended to patients in primary care. For example, opioid analgesics are one of the most commonly prescribed medicines for people presenting with LBP across various settings and their use has increased 39% in the last decade.(3) This is despite guidelines discouraging their use for LBP when pain is not severe or as first-line care before trialling other treatments.(3, 4) Similarly, gabapentinoids are ineffective and potentially harmful for the management of LBP; however their use is increasing.(4-6) On the other hand, the use of pharmacological therapies that are recommended as first line care such as NSAIDs has reduced 33% from 2004 to 2014.(3) In the United States, antidepressants are prescribed to about a quarter of patients with LBP.(7) In Portugal, 1 in 7 people with chronic LBP report using antidepressants and benzodiazepines for pain management.(8) However, LBP guidelines recommend against both medicines.(4)

In Australia, LBP is the sixth most commonly managed condition overall and the most common musculoskeletal condition managed by general practitioners (GPs).(9) A UK-based study provided some insight into aspects that shape the decision-making process of health professionals, including GPs. That study described how clinicians select treatments based upon knowing the individual patient, the patient’s LBP, and common treatment options.(10) However, this previous study did not explore the decision-making processes that influence a GP’s decision to prescribe pain medicines for LBP in-depth, particularly in relation to the processes that underpin the choice for different pain medicines. Therefore, the aim of this qualitative study was to investigate what influences GPs’ decision-making process for prescribing pain medicines for people with LBP.

For peer review only

METHODS

Design

This qualitative study was approved by The University of Sydney Human Research Ethics Committee (approval number: 2022/170) and is reported per the COREQ statement.(11)

Participant selection and recruitment

We recruited 25 GPs registered to practise in Australia who reported having provided care for at least five patients with LBP with or without sciatica in the last 12 months. Four or less is what our team thought would be representative of little to no experience in managing LBP. We defined sciatica as a back problem with radiating pain below the knee with or without neurological symptoms (e.g. reduced muscle strength, sensation, or reflexes).(12)

For peer review only - http://bmjopen.bmj.com/site/about/guidelines.xhtml

GPs were recruited via a market research company (TKW Research Group, Australia) with a large database of healthcare professionals across Australia and New Zealand. Health professionals opt into this database and therefore have already indicated a willingness to participate in research. None of the participants had a prior relationship with the study investigators. The company sent out emails to GPs in their database and scheduled interviews with those who expressed interest in the study.

Interviews and data collection

We conducted in-depth, one-on-one semi-structured interviews. Interviews were conducted by videoconference between May and June 2022, audio recorded, and transcribed verbatim. A researcher with a background in occupational therapy and experience in qualitative interviews and pain management research conducted the interviews.

An interview guide and three clinical vignettes to guide the interviews were developed by the lead author, a physiotherapist with experience in LBP research and pain management, and two experienced rheumatologists who provide care for patients with LBP (supplementary file 1). The vignettes were designed to represent three common LBP presentations: a patient with an acute exacerbation of chronic LBP, a patient with subacute sciatica (pain duration between 6-12 weeks), and a patient with chronic non-specific LBP (pain for longer than 12 weeks). Collectively, the vignettes were modelled to describe a range of factors that could play a role in a doctors' decision-making process to manage LBP (Table 1). GPs were asked to think aloud how they would manage the cases described in the vignettes.(13) For each clinical decision made by the participant, the researcher facilitating the interview asked participants to elaborate on a point, or to obtain clarification if needed (Supplementary file 2).

Analysis

Interview data were analysed using two approaches. We used content analysis to create a summary of GP's choices of pain medicines prescribed for each vignette. Content analysis combines both qualitative and quantitative methods, allowing both the content and frequency of categories to be reported.(14, 15) Two researchers initially reviewed and familiarised themselves with the transcripts, and coded all pain medicines using the following framework: medicines that would be prescribed as first, second, third, or fourth-line care, medicines that GPs would conditionally prescribe (e.g. depending on a patient characteristic), and those that GPs were against prescribing.

We used framework analysis to investigate factors that influence GP's decision-making process.(16) Two researchers coded 10 interview transcripts inductively, developed a library of codes in an iterative process, decided on a coding framework (comprising the themes) and applied it to all interviews. Codes, sub-themes, and themes were reviewed by us, and changes were made as stronger patterns linking codes to themes emerged from the data.(17) Both researchers have a background in physiotherapy and have published on the quality use of pain medicines.(18-20) Data saturation was reached (i.e. no new themes emerged).

Patient and public involvement

For peer review only - http://bmjopen.bmj.com/site/about/guidelines.xhtml

It was not appropriate or possible to involve patients or the public in the design, or conduct, or reporting, or dissemination plans of our research.

RESULTS

We interviewed 25 GPs from April to June, 2022. Most were males (64%) practising in metropolitan areas across six states of Australia. Their mean (SD) age was 53.4 (9.1), and they had a mean of 24.5 (9.3) years of practice as GPs. All but three worked full-time in private practices (Table 2). The recoding of one interview was mistakenly interrupted just before vignette #3 started and therefore there are only data from 24 GPs for that vignette.

Content analysis

Paracetamol and NSAIDs were commonly prescribed for all three vignettes. However, opioid analgesics were the pain medicines most prescribed or conditionally prescribed for the acute exacerbation of chronic LBP and sciatica vignettes, and antidepressants for the chronic LBP vignette. Codeine was the most prescribed opioid analgesic for the acute exacerbation vignette, whereas tramadol was the most common choice for the sciatica vignette. Concerns over the safety of opioids, particularly codeine, were commonly reported by GPs across all vignettes. Those concerns were often specific to a type of opioid, as many who expressed concerns over one type of opioid would still prescribe a different opioid analgesic or conditionally prescribe the same opioid. For example, this was observed in the interviews of 11 (44%) GPs in the acute exacerbation vignette (Table 3).

Anticonvulsants were the medicine class with the highest number of GPs indicating they would not prescribe for the acute exacerbation vignette: nine (36%) were against prescribing them because the patient did not have signs of neuropathic pain that would justify their use. In contrast, 16 (64%) of GPs recommended an anticonvulsant for the sciatica vignette, mostly pregabalin. GPs prescribed antidepressants for all three vignettes, more commonly for the chronic LBP vignette (n=15, 63%). Of those, most chose duloxetine (n=10, 42%) and/or amitriptyline (n=7, 29%). GPs prescribed antidepressants for different reasons for each vignette: co-morbid depression influenced decision-making for the chronic LBP vignette, whereas neuropathic pain was the key reason leading to antidepressants being prescribed for the sciatica vignette.

Factors influencing GP's decision-making to prescribe pain medicines for LBP

We identified 5 key themes that influenced GP's decision-making. Themes, subthemes, and supporting quotes are presented in table 4.

Theme 1: Prioritising personal information sources

Subtheme 1 - Previous experiences: Past clinical and personal experiences were recognised by most GPs as the most important source of information to help them make decisions. Some acknowledged that accumulated clinical experience contributed to the formulation of shortcuts that guided decision-making. Meaningful past experiences were often centred

For peer review only - http://bmjopen.bmj.com/site/about/guidelines.xhtml

around success using medicines with patients, but also personal success when they experienced pain themselves.

GPs considered patients' previous treatment experience an important source of information to assist with their decision-making. Giving patients options and discussing those options with them was often mentioned. However, in those circumstances, patient preferences and values appeared to be the key drivers of decision-making. Discussion about the evidence-base for each option was less prominent.

Subtheme 2 - Guidelines are unhelpful: Many GPs reported that guidelines were not helpful. Guidelines were noted to be particularly unhelpful when patients required more than minimal intervention such as advice and simple analgesics. Recommended interventions such as advice to stay active and education about the benign nature of most LBP were seen as problematic as it could be hard to convince patients in pain of their usefulness. One GP noted that the problem with guidelines was not necessarily their recommendations, but the time required to implement them in a context of GPs pressured for time. Clinical practice guidelines for LBP were not mentioned. Only the *Therapeutic Guidelines(21)* were mentioned by a limited number of GPs.

Subtheme 3 - Clinical training: While the applicability of guidelines was questioned, continuing education activities run by specialists and opinion leaders were highly valued. By comparison, training programs were rarely mentioned; only one GP with five years of experience mentioned the role of medical school and GP registrar training in addressing the current overuse use of opioids for LBP management.

Theme 2: Strong reliance on a biomedical model to guide prescription

Subtheme 1 - Pathoanatomical diagnosis: There was a strong sense amongst GPs that a pathoanatomical diagnosis was key to inform the choice of pain medicine to be prescribed. This was more pronounced for the chronic LBP vignette where, according to GPs, there was a more uncertain clinical presentation due to the description and duration of symptoms as well as comorbidities. Many GPs reported they would have referred the patient for imaging, and many considered the number and nature of imaging findings a key indicator of severity of the problem, which they linked to the prescription of stronger analgesics.

Subtheme 2 - Sleep patterns: Sleep influenced decision-making across the three vignettes, even though the patient in the chronic LBP vignette had no night pain or sleep disturbance. Improving sleep was an important treatment goal in the acute exacerbation vignette. For some, improving sleep was seen as the primary goal of treatment, and often led GPs to prescribe an opioid analgesics or antidepressants, or both. Advice on sleep hygiene was not mentioned.

Subtheme 3 - Pain severity: There were opposing views on the role of pain severity as a factor that influenced decision-making. While some were happy to consider stronger analgesics when pain was more severe, for others pain severity would not prompt immediate escalation to stronger analgesics. GPs were more inclined to consider escalating to stronger analgesics when discussing the acute exacerbation vignette and less inclined to do so in the

For peer review only - http://bmjopen.bmj.com/site/about/guidelines.xhtml

chronic LBP vignette (the average pain intensity was the same in both). For some GPs who would not consider escalating to stronger analgesics, their initial choice of treatment was typically a weak opioid analgesic such as codeine. Those preferring codeine mentioned being more used to the prescribing it than other opioid analgesics.

Subtheme 4 – Comorbidities: Comorbidities described in the vignettes were noted by most and influenced decision-making for some. Losing weight was often mentioned as a treatment goal in all three vignettes and led some GPs to preferring non-pharmacological approaches first or avoiding pharmacological treatments known to cause weight gain (e.g. antidepressants), despite obesity only being mentioned in the acute exacerbation vignette. The history of mild depression described in the chronic LBP vignette led many GPs to consider an antidepressant. The absence of depression in the subacute sciatica vignette was an important factor for GPs when deciding between different pharmacological options for neuropathic pain symptoms. Many acknowledged the role of antidepressants (eg duloxetine, amitriptyline) as potentially effective for symptoms described in the sciatica vignette, but most preferred anticonvulsants as the patient was young, otherwise healthy, physically active, and did not have chronic pain. In contrast, most GPs preferred to prescribe duloxetine for patients with comorbid depression and chronic pain such as the patient described in chronic LBP vignette.

Theme 3: Social factors as root causes

Subtheme 1 - Pain medicines do not address the root cause of the problem: Many GPs reported that prescribing medicines for pain for the acute exacerbation vignette would not be likely to address the root cause of the problem. Some suggested non-pharmacological treatments such as lifestyle interventions, but most GPs expressed concern about the level of social and family support that the patient had and its potential negative impact on her LBP.

Subtheme 2 - Psychosocial-based prescribing: Some GPs considered the patient's social circumstances when deciding whether to prescribe a pain medicine and what to prescribe. In the acute exacerbation vignette, many thought that the patient's social circumstances were important contributors to her pain but from different perspectives. While some emphasised the physical strain related to her carer role as a contributor to the pain, others recognised the psychological consequences of the patient's social circumstances. Those who acknowledged physical factors preferred recommending non-pharmacological options such as ergonomic devices, whereas those who recognised the psychological toll of the patient's carer role more commonly prescribed opioid analgesics or antidepressants. Other GPs were wary about whether side effects from opioid analgesics would hinder the patient's ability to care for her mother due to potential side effects, such as sedation.

Theme 4: Judicious use of pain medicines

Subtheme 1 - Keep it simple or make it complex? There were opposing views about judicious use of pain medicines. Many GPs followed the principles of the WHO analgesic ladder and avoided polypharmacy, particularly for the acute exacerbation vignette, and, to a lesser extent, the sciatica vignette. For GPs following that approach, there was a perception that the clinical course of both vignettes would be favourable and keeping prescription of pain

For peer review only - http://bmjopen.bmj.com/site/about/guidelines.xhtml

medicines to a minimum, combined with non-pharmacological measures, would be effective. Chronic pain and the history of depression were seen as barriers to improvement in the chronic LBP vignette – and therefore to a perception that the patient with chronic LBP would have been more complex to manage.

Other GPs reported that they preferred prescribing multiple pain medicines in combination, at least in the short-term, and most commonly in the sciatica and chronic LBP vignettes. In the sciatica vignette, this was driven by two factors: the notion that there was a well-established mechanism of injury (as opposed to a degenerative process with no known trigger as in the acute exacerbation vignette), and the perception that the clinical presentation was more serious as the patient had radicular symptoms. Both factors led GPs to consider stronger pain medicines such as opioid analgesics and anticonvulsants. In the chronic LBP vignette, polypharmacy often included the prescription of simple analgesics with antidepressants, most commonly duloxetine, which was driven mainly by the history of mild depression.

Theme 5: Concerns about prescription

Subtheme 1 - Drug seeking behaviour and risk of long-term misuse: A key concern for many GPs was drug seeking behaviour. GPs used multiple strategies to identify drug seeking behaviours, such as a review of medical history and intuition. Some were reluctant to prescribe opioid analgesics to patients who were not their regular patients. Some GPs mentioned being able to identify certain stereotypes more likely to be drug seekers. Although opioids were commonly considered by GPs across the three vignettes, most had concerns about their potential for misuse and addiction, particularly in relation to the chronic LBP vignette. Chronic pain and a history of depression were seen as risk factors to long-term misuse and addiction to opioid analgesics.

Subtheme 2 - Side effects of prescription pain medicines: Similarly, most concerns about side effects were related to opioid analgesics. Many GPs expressed concerns about the safety profile of specific opioid analgesics, most commonly codeine, and made decisions about which opioid analgesic to use based on their knowledge about the safety profile of opioid analgesics (e.g., sedation, constipation, nausea). In contrast, very few GPs expressed concerns about other pain medicines with potential for misuse and abuse, such as pregabalin.

Subtheme 3 - Stigma around prescription: Some GPs were aware of the stigma around prescribing pain medicines such as antidepressants. Although frequently mentioned as a treatment option for the sciatica and chronic LBP vignettes, some had concerns that prescribing antidepressants had potential for misinterpretation by patients – for example, that they were implying that the patient's pain was psychological. Lack of time during a regular consultation was seen as important barrier for prescribing antidepressants. There was a perception that longer consultations would be required to explain why the medication was being prescribed to mitigate any concerns from patients.

Subtheme 4 - Pressure from regulators: Some GPs were concerned about being pressured by regulators to not prescribe pain medicines such as opioid analgesics. Their concerns included the potential consequences of undertreating pain. No GP mentioned that pressure from regulators had changed their prescribing behaviour.

For peer review only - http://bmjopen.bmj.com/site/about/guidelines.xhtml

DISCUSSION

GPs in this study made decisions about what pain medicines to prescribe for LBP largely based on previous experiences, including their own clinical and personal experience of LBP management, not guidelines. The choice of pain medicine was influenced by a range of clinical factors, more commonly the patient's pathoanatomical diagnosis. While many adhered to principles of pain medicines prescribing, polypharmacy scenarios were also common. Concerns about drug seeking behaviour, side effects, and stigma around prescription also shaped GPs' decision-making process.

Antidepressants were considered by GPs in our study when they thought depression was linked to the patient's symptoms or as a pain medicine to treat neuropathic pain. Although there is some evidence that antidepressants may be effective for those with chronic pain and comorbid depression and neuropathic pain,(20) the role of antidepressants for chronic LBP is limited and the evidence for sciatica is inconclusive.(18) Some GPs voiced concerns about stigma around prescribing antidepressants for pain that was not observed for any other pain medicine in our study. Stigma around the use of antidepressants is a well-recognised phenomenon in the mental health field, where studies have shown that people using antidepressants for depression have a perception that their condition is more severe and that they are weak or unable to cope with their problems.(22) GPs in our study had the perception that patients may not receive the idea of being prescribed an antidepressant for their pain well.

While our GPs were aware of the harms and potential for misuse and abuse of opioid analgesics, fewer mentioned concerns with other prescription pain medicines such as anticonvulsants, which also have potential for misuse, addiction, and death.(23) The apparent lack of awareness about the harms of these medicines appears to also be common amongst the public.(24) In contrast, public health entities and government regulatory agencies have recognised the concerning potential harms of these medicines and have taken action to reduce them. Such concerns have led regulatory agencies in the UK and Australia to up schedule and to add Boxed Warnings to the Product Information and Consumer Medicine Information for pregabalin, respectively.(23, 25)

Time constraints in general practice are linked to poor adherence to guidelines, inappropriate prescription, and provision of worse care.(26-28) Furthermore, meeting guideline recommendations in primary care requires an unrealistic amount of time that GPs do not have.(29) In line with these findings, there was a perception amongst some GPs that the time required to implement guideline recommendations into practice, or to discuss prescription of certain pain medicines (e.g. antidepressants), was an important barrier to their prescription. Several studies have pointed out that GPs perceive it as nearly impossible to meet all guideline recommendations as there are too many recommendations and most are complex. For example, implementing and documenting all recommendations from guidelines for common problems seen in general practice would take up to 27 hours per day.(30) LBP guidelines involve a complex series of steps from triage to management, and may also take considerable time to be implemented.(31)

For peer review only - http://bmjopen.bmj.com/site/about/guidelines.xhtml

Our findings provide insights for future research to improve the quality use of medicines for LBP. For example, GPs in our study rarely made decisions based on guidelines or evidence. Rather, they strongly relied on previous personal experience with pain medications, and interactions with patients, colleagues, and opinion leaders. Our observations are in line with previous work that has shown that GPs make decisions mainly based on interaction with others rather than endorsed guidelines – a process known as '*mindlines*'.(32)

There are opportunities to develop educational interventions to address some of the factors that influenced decision-making that do not rely on guideline dissemination and consider the resources and sources of information most valued by GPs. One such factor is the inaccurate belief that certain pathoanatomic diagnoses on their own should guide the decision to prescribe certain pain medicines. Pathoanatomic findings are often incidental and have been shown to be mostly unrelated to the development, or severity of symptoms.(33) Interventions could also be developed to increase GPs' awareness of their own biases and heuristics as identified by our study.

The Australian Commission on Safety and Quality in Health Care has recently launched a Clinical Care Standard for LBP to improve care for LBP. One of its core messages is around the quality use of medicines. Specifically, they recommend against anticonvulsants, benzodiazepines, and antidepressants, and that clinicians only consider opioid analgesics for carefully selected patients at the lowest dose for the shortest duration possible.(34) Strategies to implement the Clinical Care Standard may need to account for the perception amongst GPs that following guidelines may lead to patients being undertreated. Our study showed that GPs value opinion leaders as a source of information, which suggests that implementation strategies to improve the quality use of medicines for LBP could involve opinion leaders, an approach that has been shown to improve compliance with evidence-based care.(35)

A strength of our study is the use of vignettes, a widely used methodology for assessing decision-making.(36) Vignettes provided a concrete starting point so that GPs could base their decisions on a detailed patient case, rather than answering abstract questions about attitudes and perceptions. However, the decision-making processes described in our study were anchored to the information provided in the vignettes and may not necessarily be generalisable to all LBP cases or truly reflect objective prescription data.(37) For example, while our study did show that GPs would most commonly prescribe or conditionally prescribe opioids for an acute exacerbation of chronic LBP (vignette 1) or subacute sciatica (vignette 2), which agrees with Australian data on prescription medicines for LBP,[3] our finding that GPs would more commonly prescribe antidepressants for the patient with chronic LBP in vignette 3 differs from existing data. It is unknown whether this difference may be due to the characteristics of the vignette and reflective of practice for patients with the characteristics described in that vignette. Our sample was composed mostly of very experienced GPs practising in metropolitan areas. Their views may differ from those in earlier stages of their careers. For example, the youngest GP in our sample mentioned the role of University and GP training in teaching GPs about the lack of effectiveness and harms of

For peer review only - http://bmjopen.bmj.com/site/about/guidelines.xhtml

opioids for LBP. That view was not shared by any other experienced GP, which could indicate recent changes in teaching curricula. GPs practising in rural areas are more likely to prescribe opioid analgesics for LBP.(3) However, our sample lacked GPs practising in those areas, limiting our ability to contrast potential differences in drivers of decision-making between those areas.

CONCLUSIONS

We identified several aspects of decision-making that help explain the current profile of pain medicines prescribed for LBP in by GPs. Themes identified by our study could inform future implementation strategies to improve the quality use of medicines for LBP.

CONTRIBUTORS

Conceptualisation: GF, JA, CWC, BR, CN, CAS, AM, RD, CM; Methodology: GF, JA; Validation: GF, JZ; Formal analysis: GF, JZ; Investigation: GF, JZ, CJ; Resources: GF; Data curation: GF; Supervision: CM; Project administration: GF; Funding acquisition: GF, CWC, CAS, BR, AM, RD, CM; Writing – Original draft: GF; Writing – Review & editing: JZ, JA, CJ, CWC, BR, CN, CAS, AM, RD, CM

TRANSPARENCY

The lead author (GF) affirms that this manuscript is an honest, accurate, and transparent account of the study being reported; that no important aspects of the study have been omitted; and that any discrepancies from the study as planned (and, if relevant, registered) have been explained.

FUNDING

This study was partially funded by a seed grant from the Australian New Zealand Musculoskeletal Clinical Trials Network (ANZMUSC). GEF, JZ, JA, C-WC L, and CM are supported by National Health and Medical Research Council (NHMRC) fellowships (APP2009808, APP1194105, APP2017278, APP1193939, and APP11094283, respectively). CM is also supported by Centre for Research Excellence grants (APP1134856, APP1171459, APP2006545).

COMPETING INTERESTS DECLARATION

The Sydney Pharmacy School receives funding from GlaxoSmithKline for a postgraduate scholarship supervised by AM. CM has received research grants from various government and not for profit agencies. Flexeze provided heat wraps at no cost for the SHaPED trial for which CM, BR, and CN are investigators. Other authors have no interests to declare.

ETHICAL APPROVAL

The University of Sydney Human Research Ethics Committee approved this research (approval number: 2022/170).

DATA AVAILABILITY

Data are available upon request.

DATA SOURCE

Data collected by the investigators.

LICENSE STATEMENT

For peer review only

The corresponding author (GF) has the right to grant and does grant on behalf of all authors of the Work (as defined in the below author licence), an exclusive licence and/or a non-exclusive licence for contributions from authors who are: i) UK Crown employees; ii) where BMJ has agreed a CC-BY licence shall apply, and/or iii) in accordance with the terms applicable for US Federal Government officers or employees acting as part of their official duties; on a worldwide, perpetual, irrevocable, royalty-free basis to BMJ Publishing Group Ltd ("BMJ") its licensees and where the relevant Journal is co-owned by BMJ to the co-owners of the Journal, to publish the Work in BMJ Quality & Safety and any other BMJ products and to exploit all rights, as set out in our licence.

The corresponding author (GF) accepts and understands that any supply made under these terms is made by BMJ to the Submitting Author unless you are acting as an employee on behalf of your employer or a postgraduate student of an affiliated institution which is paying any applicable article publishing charge ("APC") for Open Access articles. Where the Submitting Author wishes to make the Work available on an Open Access basis (and intends to pay the relevant APC), the terms of reuse of such Open Access shall be governed by a Creative Commons licence – details of these licences and which Creative Commons licence will apply to this Work are set out in our licence referred to above.

REFERENCES

1. Australian Institute of Health and Welfare. Back problems. Canberra: AIHW; 2020.
2. Williams CM, Maher CG, Hancock MJ, McAuley JH, McLachlan AJ, Britt H, et al. Low back pain and best practice care: A survey of general practice physicians. Arch Intern Med. 2010;170(3):271-7.
3. Mathieson S, Valenti L, Maher CG, Britt H, Li Q, McLachlan AJ, et al. Worsening trends in analgesics recommended for spinal pain in primary care. Eur Spine J. 2018;27(5):1136-45.
4. NICE. Low back pain and sciatica in over 16s: assessment and management 2020 [Available from: https://www.nice.org.uk/guidance/ng59.
5. Enke O, New HA, New CH, Mathieson S, McLachlan AJ, Latimer J, et al. Anticonvulsants in the treatment of low back pain and lumbar radicular pain: a systematic review and meta-analysis. Cmaj. 2018;190(26):E786-e93.
6. Mathieson S, Maher CG, McLachlan AJ, Latimer J, Koes BW, Hancock MJ, et al. Trial of Pregabalin for Acute and Chronic Sciatica. New England Journal of Medicine. 2017;376(12):1111-20.
7. Ivanova JI, Birnbaum HG, Schiller M, Kantor E, Johnstone BM, Swindle RW. Real-world practice patterns, health-care utilization, and costs in patients with low back pain: the long road to guideline-concordant care. Spine J. 2011;11(7):622-32.
8. Gouveia N, Rodrigues A, Ramiro S, Eusébio M, Machado PM, Canhão H, et al. The Use of Analgesic and Other Pain-Relief Drugs to Manage Chronic Low Back Pain: Results from a National Survey. Pain Practice. 2017;17(3):353-65.
9. Britt H MG, Bayram C, Henderson J, Valenti L, Harrison C, Pan Y, Charles J,, Pollack AJ CT, Gordon J, Wong C. A decade of Australian general practice activity 2006–07 to 2015–16. Sydney: Sydney University Press, 2016; 2016.
10. Bishop FL, Dima AL, Ngui J, Little P, Moss-Morris R, Foster NE, et al. "Lovely Pie in the Sky Plans": A Qualitative Study of Clinicians' Perspectives on Guidelines for Managing Low Back Pain in Primary Care in England. Spine (Phila Pa 1976). 2015;40(23):1842-50.
11. Tong A, Sainsbury P, Craig J. Consolidated criteria for reporting qualitative research (COREQ): a 32-item checklist for interviews and focus groups. Int J Qual Health Care. 2007;19(6):349-57.
12. Jensen RK, Kongsted A, Kjaer P, Koes B. Diagnosis and treatment of sciatica. BMJ. 2019;367:l6273.
13. Lundgren-Laine H, Salantera S. Think-aloud technique and protocol analysis in clinical decision-making research. Qual Health Res. 2010;20(4):565-75.
14. O'Keeffe M, Michaleff ZA, Harris IA, Buchbinder R, Ferreira GE, Zadro JR, et al. Public and patient perceptions of diagnostic labels for non-specific low back pain: a content analysis. European Spine Journal. 2022;31(12):3627-39.

For peer review only - http://bmjopen.bmj.com/site/about/guidelines.xhtml

15. Ferreira GE, Zadro J, O'Keeffe M, Chen M, Gao Y, Hu Z, et al. A content analysis of online information about the benefits and harms of spine surgery. Braz J Phys Ther. 2022;26(2):100398.
16. Gale NK, Heath G, Cameron E, Rashid S, Redwood S. Using the framework method for the analysis of qualitative data in multi-disciplinary health research. BMC Medical Research Methodology. 2013;13(1):117.
17. Ayre J, McCaffery KJ. Research Note: Thematic analysis in qualitative research. J Physiother. 2022;68(1):76-9.
18. Ferreira GE, McLachlan AJ, Lin CC, Zadro JR, Abdel-Shaheed C, O'Keeffe M, et al. Efficacy and safety of antidepressants for the treatment of back pain and osteoarthritis: systematic review and meta-analysis. BMJ. 2021;372:m4825.
19. Ferreira GE, Machado GC, Abdel Shaheed C, Lin CC, Needs C, Edwards J, et al. Management of low back pain in Australian emergency departments. BMJ Qual Saf. 2019;28(10):826-34.
20. Ferreira GE, Abdel-Shaheed C, Underwood M, Finnerup NB, Day RO, McLachlan A, et al. Efficacy, safety, and tolerability of antidepressants for pain in adults: overview of systematic reviews. BMJ. 2023;380:e072415.
21. Therapeutic Guidelines. Melbourne: Therapeutic Guidelines Limited; [Available from: https://tg.org.au.
22. Interian A, Martinez IE, Guarnaccia PJ, Vega WA, Escobar JI. A qualitative analysis of the perception of stigma among Latinos receiving antidepressants. Psychiatr Serv. 2007;58(12):1591-4.
23. Mathieson S, Lin CC, Underwood M, Eldabe S. Pregabalin and gabapentin for pain. BMJ. 2020;369:m1315.
24. Mathieson S, O'Keeffe M, Traeger AC, Ferreira GE, Abdel Shaheed C. Content and sentiment analysis of gabapentinoid-related tweets: An infodemiology study. Drug and Alcohol Review. 2022;n/a(n/a).
25. Therapeutic Goods Administration. Pregabalin and gabapentin. Safety advisory - enhanced warnings relating to abuse and dependence 2021 [Available from: https://www.tga.gov.au/news/safety-alerts/pregabalin-and-gabapentin.
26. Tamblyn R, Berkson L, Dauphinee WD, Gayton D, Grad R, Huang A, et al. Unnecessary prescribing of NSAIDs and the management of NSAID-related gastropathy in medical practice. Ann Intern Med. 1997;127(6):429-38.
27. Campbell SM, Hann M, Hacker J, Burns C, Oliver D, Thapar A, et al. Identifying predictors of high quality care in English general practice: observational study. BMJ. 2001;323(7316):784.
28. Tsiga E, Panagopoulou E, Sevdalis N, Montgomery A, Benos A. The influence of time pressure on adherence to guidelines in primary care: an experimental study. BMJ Open. 2013;3(4):e002700.
29. Privett N, Guerrier S. Estimation of the Time Needed to Deliver the 2020 USPSTF Preventive Care Recommendations in Primary Care. Am J Public Health. 2021;111(1):145-9.
30. Porter J, Boyd C, Skandari MR, Laiteerapong N. Revisiting the Time Needed to Provide Adult Primary Care. J Gen Intern Med. 2023;38(1):147-55.
31. Bardin LD, King P, Maher CG. Diagnostic triage for low back pain: a practical approach for primary care. Med J Aust. 2017;206(6):268-73.
32. Gabbay J, le May A. Evidence based guidelines or collectively constructed "mindlines?" Ethnographic study of knowledge management in primary care. Bmj. 2004;329(7473):1013.
33. Kasch R, Truthmann J, Hancock MJ, Maher CG, Otto M, Nell C, et al. Association of Lumbar MRI Findings with Current and Future Back Pain in a Population-based Cohort Study. Spine. 2022;47(3).
34. Australian Commission on Safety and Quality in Health Care.Low Back Pain Clinical Care Standard. Sydney: ACSQHC; 2022.
35. Flodgren G, O'Brien MA, Parmelli E, Grimshaw JM. Local opinion leaders: effects on professional practice and healthcare outcomes. Cochrane Database Syst Rev. 2019;6(6):Cd000125.
36. Evans SC, Roberts MC, Keeley JW, Blossom JB, Amaro CM, Garcia AM, et al. Vignette methodologies for studying clinicians' decision-making: Validity, utility, and application in ICD-11 field studies. Int J Clin Health Psychol. 2015;15(2):160-70.
37. Alexander CS, Becker HJ. The Use of Vignettes in Survey Research. Public Opinion Quarterly. 1978;42(1):93-104.

For peer review only - http://bmjopen.bmj.com/site/about/guidelines.xhtml

Table 1. Features of each clinical vignette.

	Vignette 1	Vignette 2	Vignette 3
**Patient characteristics**			
Female	X		X
Male		X	
Middle-aged	X	X	X
Physical activity			
No physical activity	X		
Insufficient physical activity			X
Physically active		X	
**Social aspects**			
Children			X
Primary carer for older person	X		
**Type of low back pain**			
Atraumatic back pain	X	X	X
Back pain initiated after a well-defined event		X	
Back pain with a clear radicular component		X	
Back pain with referred leg pain			X
**Pain characteristics**			
Night pain	X	X	
Sleep disturbance	X		
Back pain exacerbated by movements/postures		X	X
Acute-on-chronic pain	X		
Sub-acute (pain between 6 and 12 weeks)		X	
Chronic (pain > 12 weeks)			X
Movement restriction	X	X	
Limitations to work		X	
**Comorbidities**			
Obesity	X		
Gastro-oesophageal reflux disease	X		
High cholesterol	X		
Other musculoskeletal conditions	X		
Diabetes			X
Depression (mild)			X
**Healthcare use**			
General practitioner for low back pain		X	
Physiotherapy for low back pain			X
Acupuncture for low back pain			X
Psychologist for mild depression			X
Previous imaging	X		
Previous/current use of pain medicines	X	X	X
Paracetamol, NSAIDs	X		X
Opioids		X	

For peer review only

Table 2. Characteristics of participants (n = 25)

Age, mean (SD)	53.4 (9.1)
Sex, n (%)	
Female	9 (36%)
Male	16 (64%)
State/territory	
New South Wales	9 (36%)
Queensland	6 (24%)
Victoria	5 (20%)
South Australia	2 (8%)
Western Australia	2 (8%)
Tasmania	1 (4%)
Remoteness[1]	
Metropolitan	22 (88%)
Rural/remote	3 (12%)
Number of patients with low back pain seen per year, n (%)	
5-25	2 (8%)
26-50	4 (16%)
51-100	3 (12%)
>100	16 (64%)
Years of experience, n (SD) [min – max]	24.6 (9.3) [5 – 40]
Weekly clinical workload (hours), mean (SD) [min – max]	38 (7.7) [14 – 50]

[1]Defined per the Australian Statistical Geography Standard; GPs working in metropolitan areas are those whose practice is located in a Greater Capital City Statistical Area.

For peer review only

For peer review only - http://bmjopen.bmj.com/site/about/guidelines.xhtml

Table 3. Frequency of pain medicines that GPs would prescribe, would conditionally prescribe, were against prescribing, or that were not mentioned for each vignette.

	Paracetamol	NSAIDs	Opioids	Antidepressants	Anticonvulsants	Oral steroids	Benzodiazepines
**Vignette 1 (n = 25)**							
Would prescribe	13	17	8	6	1	0	0
Would conditionally prescribe	0	3	14	3	0	1	3
Against prescribing	0	2	2	6	9	1	1
Not mentioned	12	3	1	10	15	23	21
**Vignette 2 (n = 25)**							
Would prescribe	8	16	7	6	11	3	0
Would conditionally prescribe	0	0	12	7	5	2	0
Against prescribing	0	2	6	3	5	3	1
Not mentioned	17	7	0	9	4	17	24
**Vignette 3 (n = 24)***							
Would prescribe	8	8	8	10	5	0	1
Would conditionally prescribe	1	1	3	5	2	0	0
Against prescribing	0	2	4	0	1	2	0
Not mentioned	15	13	9	9	16	22	23

GP, General practitioner; NSAIDs, non-steroidal anti-inflammatories
*Data from 24 GPs shown. The interview with one GP discussing vignette 3 was not recorded due to technical issues.

For peer review only - http://bmjopen.bmj.com/site/about/guidelines.xhtml

Table 4. Themes, subthemes, and supporting quotes.

Themes	Subthemes	Supporting quotes
Prioritising personal information sources	Previous experience	• As an experienced GP of just over 20 years, you have scripts in your mind that bring you to a sort of automatic pilot approach. (GP #20, male, 21 years of experience)<br>• I'd probably just give her the options of weather she wants some anti-inflammatory, something with codeine, and antidepressant. (GP #3, female, 23 years of experience)<br>• I've had radicular pain myself, and the only thing that fixed me was Lyrica. I actually had little faith in Lyrica before I used it, but nothing else touched my pain. A lot of my approach to radicular pain is based on my own experience. (GP #1, female, 30 years of experience)
	Guidelines are unhelpful	• Sometimes I find guidelines are not actually practical. If they say 'oh tell the patient they need to keep fit and tell them that pain is not a bad sign and that they should keep active and a little bit of pain is ok', it can be hard to convince patients of that. (GP #1, female, 30 years of experience)
	Clinical training	• Probably what I've heard at specialist meetings when the specialists have spoken. I went to one last night and the neurosurgeon was basically saying you try and avoid going on the path of long-acting opioids, and you can use a quick acting opioid. (GP #6, female, 35 years of experience).<br>• Part of it is what we what we've learned through GP registrar training and medical school, where there's a strong emphasis on not using opioid painkillers in for the management of back pain (GP #17, male, 5 years of experience)
Strong reliance on a biomedical model to guide prescription	Pathoanatomical diagnosis	• When we do the imaging, it's going to support our treatment more. If you find one of two nerves there affecting him, we can start him on pregabalin 25 mg (GP #23, female, 28 years of experience)<br>• If we found multiple levels of a minor disc bulge or something, I would be happier to give her something stronger, but if the MRI just showed that she had a disc bulge at one level and everything else looks fine, there's no nerve impingement, I don't think that would warrant it. I'd be happy to try amitriptyline than other medications as a first line. (GP #9, female, 25 years of experience).
	Sleep patterns	• I'd be happy to give her some panadeine [NB: codeine + paracetamol] straight away because it's keeping her awake at night, and that's always a bit of a problem. She wakes up because of the pain… I mean you don't want people waking up with pain, so give her a bit of mild pain relief. (GP #22, male, 39 years of experience)
	Pain severity	• No because she might still respond to what I've decided to use, it might just be temporary that she's eight or nine [NB: pain out of 10] (GP #4, female, 30 years of experience)
	Comorbidities	• There are some other antidepressants like Cymbalta which can act as a neuropathic agent as well as an antidepressant. So there are antidepressants which we can use which help patients manage their depression as well as well as their pain. (GP #19, male, 30 years of experience)
Social factors as root causes	pain medicines do not address the root cause of the problem	• We don't want her having a fall with her mum and we don't want her experiencing side effects that may affect her ability to care for her mother. (GP #7, male, 18 years of experience)
	Psychosocial-based prescribing	• Since she's getting relief with paracetamol and ibuprofen, I think the next step is to find if there are any associated issues in terms of looking after her mum. Can we get some home help… refer mum to aged care services? I mean she might be able to get NDIS help [NB: government support] as well (GP #11, male, 20 years of experience)

For peer review only - http://bmjopen.bmj.com/site/about/guidelines.xhtml

Judicious use of pain medicines	Keep it simple or make it complex?	• They get flare ups here and there and I think you don't jump up and down as long as you know exactly the origin of the pain and you know you excluded that there is more to it than just what's presenting at this stage, and I think let's just keep it simple. And you can always go back and increase the medication or order further investigations if things don't subside, but I don't hit them harder on the 1st presentation. (GP #15, male, 30 years of experience)<br><br>• I tend to like to use a smaller dose of a lot of things rather than a bigger dose of one or two things. So I'd write a little plan and it would have a sliding scale so it would start off with the Panadol three times a day plus the Nurofen. That's the foundations of the house, and then I would add into that tramadol sustained release 150 to 100 twice a day, and then I would usually add in amitriptyline 10 to 20 milligrams before bed. (GP #25, female, 30 years of experience)
Concerns about prescription	drug seeking behaviour and risk of long-term misuse	• It's a chronic, insidious, ongoing bit of depression. And it just typically… It's just super clean. She gets started on endone and then get stuck on endone and that becomes their whole life. And they just slowly over a course of years dose-escalate. And they feel better. Because of the euphoria associated with the oxycodone. Terrible. (GP #16, male, 34 years of clinical experience)
	Side effects of prescription pain medicines	• Opioids induce hyperalgesia, which comes with a lot of opioids such as codeine, which is a poor analgesic. I don't use that either. I basically use 3 analgesics in my practice: tramadol, buprenorphine, and tapentadol. (GP #19, Male, 30 years of experience)
	Stigma around prescription	• It could be done, but it would involve a very lengthy consultation and we as GPs don't have half an hour to 45 minutes to discuss something a little bit more wayward. (GP #7, male, 18 years of experience)
	Pressure from regulators	• The guidelines at the moment for non-opioid medication are clear as far as we can all see. I don't know how much that'll go the wrong way and we'll end up not treating anybody pains eventually. We get sued for not treating anybody pain and the pain and suffering and next thing you know the guidelines will change, you know? But you can't win, can you? (GP #14, male, 40 years of experience)<br><br>• Department of Health want us not to give pain medicine at all for low back pain but sometimes it's not realistic. Sometimes we need to help the patient even though I mean, potentially they can be additive of course, but, realistically, the patient is in so much pain, they can't move. This is cruel. Not giving opioid medicines, at least for a few days. I'm not the type of person that gives everybody strong pain medicines, but I think there is a role in some patients. (GP #18, male, 27 years of experience)

For peer review only - http://bmjopen.bmj.com/site/about/guidelines.xhtml

General practitioners' decision-making process to prescribe pain medicines for low back pain: a qualitative study

Contents

Supplementary file 1. Vignettes 2

Supplementary file 2. Interview guide. 5

For peer review only

Supplementary file 1. Vignettes

Prompt: I will now present to you three clinical vignettes describing different low back pain presentations. After each vignette I am going to ask you a series of questions. Please draw on your prior experience to answer the questions about how you would manage these back pain patients. There are no right or wrong answers. We are interested in honest opinions, not idealised ones (for example, what you should do). Remember that your data will be anonymised.

VIGNETTE 1

- Patient info: Female patient, 57 years old, BMI 33 (height: 1.60m; weight: 85kg)

- Complaint: atraumatic onset low back pain that started 2 weeks ago. Average pain 5/10; worse in the evening/at night with pain 7/10.

- History:
  - Low back pain for 20+ years (ongoing mild pain 2/10) with occasional moderate flares (pain up to 5/10) that typically resolve in a few days. Previous MRIs (last one 3 years ago) showed the following: At L5/S1 there is vertebral endplate bone marrow oedema. The distinction between the nucleus and annulus is lost with a with loss of disc height.
  - She has sought care for back pain a few times before, but typically self-manages the pain with simple painkillers (Paracetamol and occasionally ibuprofen as needed). She is seeking care for this episode because it's taking longer to resolve, and the pain is more intense in the evening/at night
  - Patient is having difficulty sleeping – she wakes up because of pain when turning in bed.
  - Patient is sedentary and is the primary carer of her 88-year-old mother who is bed-bound due to a stroke 12 months ago.
  - Comorbidities: Obesity, GORD, high cholesterol (daily atorvastatin), medial compartment R knee osteoarthritis (also treats with simple analgesics as needed).

- Physical examination:
  - No evidence of radicular symptoms: straight leg raise, sensation, reflexes, and lower limb strength are normal. No signs of infection or midline tenderness in the lumbar spine. Tenderness over the paraspinal muscles is present.
  - Movements in every direction are slightly more painful (6/10)

For peer review only

VIGNETTE 2

- Patient info: 45-year-old male, BMI 26 (height: 1.83m; weight: 87kg)

- Complaint: Acute onset low back pain with shooting stabbing pain radiating down the back of the right leg to the foot and associated with pins and needles in the same area. This commenced 8 weeks ago after trying to lift a heavy box while visiting family in rural NSW. Main complaint is leg pain (5/10), but he also has pain in the lower back (3/10). Sitting for >1h aggravates leg pain (7/10), sit-to-standing and vice-versa are quite painful (7/10).

- History:
  - No history of low back pain or any other musculoskeletal condition prior to this episode. Patient is physically active, swims and runs twice a week. Sitting for more than 1 hour aggravates pain and is severely limiting his ability to work. He has not taken any time off work.
  - Patient went to the local general practitioner the day after pain onset who prescribed codeine 30mg QID for 1 week. No improvement was noted. No significant side effects were noted either besides constipation.
  - Patient has not reported any significant sleep disturbance

- Physical examination:
  - Pain follows a dermatomal distribution (S1), with altered sensation to light touch. Muscle strength and reflexes are preserved.
  - No signs of infection or midline tenderness in the lumbar spine.
  - Straight leg raise positive at ~30deg; crossed straight leg raise test is negative
  - Flexion and extension movements are limited and increase leg pain (7/10). Rotations and inclinations do not change symptoms.
  - Cauda equina syndrome and Other red flags were excluded

For peer review only

VIGNETTE 3

- Patient info: Female patient, 52 years old, BMI 30 (height: 1.63m; weight: 79kg)

- Complaint: Insidious onset low back pain radiating to the L thigh that started 6 months ago. Patient cannot recall an event that triggered the pain. Pain is bilateral in the lower back and radiates to the L posterior thigh with the same average intensity (5/10). Standing up/walking for too long (>1h) increases pain (6-7/10). Sitting/lying down reduces pain. She does not have trouble sleeping.

- History:

    - No history of low back pain or any other musculoskeletal condition prior to this episode. Patient is married with 3 children (youngest is 20 years-old),
    - Patient has recently been diagnosed with type II diabetes (she takes metformin daily and has been going to the gym 3 times/week for about 45 minutes where she does low-moderate intensity resistance training since the diagnosis – 2 years ago. Her sugar levels are currently controlled – around 5mmol/L before meals. Kidney function is normal). She was diagnosed with mild depression two years ago and has been supported by a psychologist with training in cognitive behavioural therapy. No other pharmacological treatments are used for depression.
    - She uses over-the-counter analgesics (Paracetamol and Ibuprofen) regularly on an as-needed basis to manage the pain. She has seen a physiotherapist (core strengthening exercises) and an acupuncturist but has not responded to either treatment.

- Physical examination:
    - No abnormalities on neurological exam (sensation, strength, and reflexes are all normal).
    - No signs of infection or midline tenderness in the lumbar spine.
    - Straight leg raise and crossed Straight leg raise negative.
    - Lumbar spine movements are limited mostly by lack of flexibility. End-range lumbar extension increases pain momentarily (7/10), which reduces as soon as patient returns to neutral position.

For peer review only

Supplementary file 2. Interview guide.

Questions - Choosing between different types of pharmacological treatments

Prompt: Answer the following questions based on the patient vignette you just read. Using the information from the vignette, I would like you to think aloud your clinical reasoning.

- Would you typically prescribe a pain medicine for the patient described in the vignette?

- Could you elaborate on which pain medicines you would prescribe and why you would prescribe them *[remind the participant of the medicines they mentioned]*?

Prompt participant to talk about their rationale to prescribe/recommend certain medicines; what kind of thought process they follow, sources of information that they use to make their decision:

- *Clinical presentation (e.g. affected sleep in vignette 1; neuropathic pain features in vignette 2; depression in vignette 3, etc.)*
- *Severity of symptoms*
- *Known efficacy from trials and systematic reviews*
- *Guideline recommendation*
- *Safety/side effects*
- *Previous use by patient*
- *Previous success with other patients*
- *Demographic factors*
- *Mechanisms of action of the drug*
- *Intuition*

Questions – influence of factors not mentioned by the participant

- How do aspects like (eg age, gender, comorbidities, description of pain, etc.) factor in your decision to prescribe 'ABC' *[the pain medicines mentioned by the participant]*?

Questions - Alternatives

- What about pain medicines 'XYZ'? Would you prescribe any of these for the vignette? *[mention pain medicines that the participant did not mention eg strong opioids [oxycodone endone], weak opioids [codeine], gabapentin, pregabalin, amitriptyline, oral steroids, etc.)]*

- Are there any circumstances where you would prescribe XYZ instead or/in addition to ABC for the patient in this vignette?

Questions – external factors influencing decision-making

- Would your decision-making change if you were pressed for time? *[eg do they report using any heuristic/rules of thumb when they are time-limited?]*
- Would your decision-making change depending on patients' opinions on the treatments you typically prescribe?

For peer review only

COREQ (COnsolidated criteria for REporting Qualitative research) Checklist

A checklist of items that should be included in reports of qualitative research. You must report the page number in your manuscript where you consider each of the items listed in this checklist. If you have not included this information, either revise your manuscript accordingly before submitting or note N/A.

Topic	Item No.	Guide Questions/Description	Reported on Page No.
**Domain 1: Research team and reflexivity**			
Personal characteristics			
Interviewer/facilitator	1	Which author/s conducted the interview or focus group?	
Credentials	2	What were the researcher's credentials? E.g. PhD, MD	
Occupation	3	What was their occupation at the time of the study?	
Gender	4	Was the researcher male or female?	
Experience and training	5	What experience or training did the researcher have?	
Relationship with participants			
Relationship established	6	Was a relationship established prior to study commencement?	
Participant knowledge of the interviewer	7	What did the participants know about the researcher? e.g. personal goals, reasons for doing the research	
Interviewer characteristics	8	What characteristics were reported about the inter viewer/facilitator? e.g. Bias, assumptions, reasons and interests in the research topic	
**Domain 2: Study design**			
Theoretical framework			
Methodological orientation and Theory	9	What methodological orientation was stated to underpin the study? e.g. grounded theory, discourse analysis, ethnography, phenomenology, content analysis	
Participant selection			
Sampling	10	How were participants selected? e.g. purposive, convenience, consecutive, snowball	
Method of approach	11	How were participants approached? e.g. face-to-face, telephone, mail, email	
Sample size	12	How many participants were in the study?	
Non-participation	13	How many people refused to participate or dropped out? Reasons?	
Setting			
Setting of data collection	14	Where was the data collected? e.g. home, clinic, workplace	
Presence of non-participants	15	Was anyone else present besides the participants and researchers?	
Description of sample	16	What are the important characteristics of the sample? e.g. demographic data, date	
Data collection			
Interview guide	17	Were questions, prompts, guides provided by the authors? Was it pilot tested?	
Repeat interviews	18	Were repeat inter views carried out? If yes, how many?	
Audio/visual recording	19	Did the research use audio or visual recording to collect the data?	
Field notes	20	Were field notes made during and/or after the inter view or focus group?	
Duration	21	What was the duration of the inter views or focus group?	
Data saturation	22	Was data saturation discussed?	
Transcripts returned	23	Were transcripts returned to participants for comment and/or	

For peer review only

Topic	Item No.	Guide Questions/Description	Reported on Page No.
		correction?	
**Domain 3: analysis and findings**			
Data analysis			
Number of data coders	24	How many data coders coded the data?	
Description of the coding tree	25	Did authors provide a description of the coding tree?	
Derivation of themes	26	Were themes identified in advance or derived from the data?	
Software	27	What software, if applicable, was used to manage the data?	
Participant checking	28	Did participants provide feedback on the findings?	
Reporting			
Quotations presented	29	Were participant quotations presented to illustrate the themes/findings? Was each quotation identified? e.g. participant number	
Data and findings consistent	30	Was there consistency between the data presented and the findings?	
Clarity of major themes	31	Were major themes clearly presented in the findings?	
Clarity of minor themes	32	Is there a description of diverse cases or discussion of minor themes?	

Developed from: Tong A, Sainsbury P, Craig J. Consolidated criteria for reporting qualitative research (COREQ): a 32-item checklist for interviews and focus groups. *International Journal for Quality in Health Care*. 2007. Volume 19, Number 6: pp. 349 – 357

Once you have completed this checklist, please save a copy and upload it as part of your submission. DO NOT include this checklist as part of the main manuscript document. It must be uploaded as a separate file.

For peer review only
